# Epidermal growth factor dampens pro-inflammatory gene expression induced by interferon-gamma in global transcriptome analysis of keratinocytes

**DOI:** 10.1186/s12864-025-11237-1

**Published:** 2025-02-10

**Authors:** David C. Gibbs, Myles R. McCrary, Carlos S. Moreno, Lindsey Seldin, Chaoran Li, Nourine A. H. Kamili, Brian P. Pollack

**Affiliations:** 1 Atlanta Veterans Affairs Medical Center, Decatur, GA 30033 USA; 2 Department of Dermatology, Emory University School of Medicine, Atlanta, GA 30322 USA; 3 Dermatology and Cutaneous Surgery, Morsani College of Medicine, University of South Florida, Tampa, FL 33612 USA; 4 Department of Anatomic and Clinical Pathology, University of South Florida, Tampa, FL 33612 USA; 5 Department of Pathology and Laboratory Medicine, Emory University School of Medicine, Atlanta, GA 30322 USA; 6 Department of Cell Biology, Emory University, Atlanta, GA 30322 USA; 7 Department of Microbiology and Immunology, Emory University School of Medicine, Atlanta, GA 30322 USA; 8 Winship Cancer Institute, Emory University School of Medicine, Atlanta, GA 30322 USA

**Keywords:** Epidermal growth factor, Interferon, Skin, Keratinocyte, Epidermal growth factor receptor (EGFR), RNA-seq, Transcriptomics, Cancer, Immunomodulation

## Abstract

**Background:**

Epidermal growth factor receptor inhibitors (EGFRIs) are used to treat certain cancers but frequently cause cutaneous inflammation that can hinder treatment. This is due in part to the effects of EGFRIs on pro-inflammatory signaling by interferon-γ (IFN-γ). However, the impact of EGFR ligands (i.e. EGF) on interferon signaling is unclear. The purpose of this study was to investigate the impact of EGF on IFN-γ transcriptional responses on a genome-wide scale in keratinocytes.

**Methods:**

RNA-seq was performed in human keratinocyte (HaCaT) cells treated with IFN-γ, EGF, both, or neither (control). Differentially expressed genes in each treatment group, relative to control, were identified using DESeq2 with a false discovery rate (FDR) threshold of 0.01. Associated biologic processes and gene pathways were examined in gene-set enrichment analyses. Correlations between gene expression were investigated in vivo using RNA-seq data from biopsies of psoriatic and matched normal skin, which were collected from 116 individuals with psoriasis enrolled in the AMAGINE randomized clinical trials.

**Results:**

Of the 2,792 differentially expressed genes following IFN-γ treatment, 2,083 (75%) were no longer differentially expressed when EGF was added. IFN-γ-induced genes with significantly lower expression in the presence of EGF included *CXCL10*, *IL-6*,* IL-1 A*,* HLA-DMA*, and *GBP5* (activator of the NLRP3 inflammasome); the top enriched biologic processes and pathways were related to MHC-class II antigen presentation (GO:0019886) and cytokine signaling (KEGG:04060). Consistent with our in vitro findings, the expression of *CXCL10* and *GBP5*, as well as the combined expression z-scores of genes in the enriched MHC-class II and cytokine signaling pathways, were significantly lower in skin biopsies with higher EGF expression compared to those with lower EGF expression among individuals with psoriasis.

**Conclusions:**

Our findings suggest that the pro-inflammatory IFN-γ-induced transcriptome may be globally attenuated by EGF in keratinocytes, supporting an immunomodulatory role of EGF in the skin. These studies provide insights for the non-canonical immunomodulatory role of EGF signaling and why blocking EGFR signaling (e.g., with EGFRIs) can cause cutaneous inflammation.

**Supplementary Information:**

The online version contains supplementary material available at 10.1186/s12864-025-11237-1.

## Introduction

The epidermal growth factor (EGF) has a well-established role in cellular proliferation, with aberrant expression and/or activating mutations of the EGF receptor (EGFR) linked to the development of several cancers [1]. As such, EGFR inhibitors (EGFRIs) are increasingly used in cancer precision therapies. The most common side effect of these agents are inflammatory skin reactions, suggesting an immunomodulatory role of EGFR-mediated signaling that is incompletely understood [1, 2]. These side effects are notable since they: (i) are associated with patient survival, (ii) may predict treatment responses, and (iii) when severe, can disrupt potentially life-saving treatments [3, 4]. Thus, a better understanding of how EGFR-mediated signaling influences cutaneous inflammatory pathways is needed.

Prior studies have shown that the EGFR ligand EGF dampens the interferon-γ (IFN-γ)-mediated induction of major histocompatibility complex (MHC) class II molecules, which is essential in adaptive immune responses, including anti-tumor immune responses [5]. Additionally, EGFR inhibitors promote the expression of MHC molecules induced by IFN-γ in multiple in vitro keratinocyte models and in skin biopsies of patients after initiating EGFR inhibitor therapy [6]. However, it is unknown how EGFR ligands, including EGF, influence pro-inflammatory gene expression induced by IFN-γ in keratinocytes. This is particularly relevant since the crosstalk between EGFR and IFN-γ signaling may be mediated by the shedding of membrane-bound EGFR ligands (i.e., EGF) by surface proteases following IFN-γ receptor complex activation [7].

Here, we investigated how EGF modulates immune-related gene expression induced by IFN-γ using global transcriptomic analysis in human keratinocytes. Given that EGFRIs enhance the induction of IFN-induced genes, including those of the MHC, we hypothesized that EGF may have the opposite effect and repress the induction of genes induced by IFN-γ. We found that the transcriptional response to IFN-γ is profoundly altered in the presence of EGF on a genome-wide level in keratinocytes and correlated with gene expression findings from skin samples collected from individuals with psoriasis.

## Methods

### Cells

HaCaT cells (Addex Biological) were grown in DMEM with 1 g/L -glucose and L-glutamine (Thermo Fisher Scientific) with 10% fetal bovine serum (FBS) at 5% CO_2_ without antibiotics. HaCaT cells were validated by short tandem repeat analysis at the Emory Integrated Genomics Core.

### Reagents and treatment of cells

Human IFN-γ (Peprotech, NJ) and EGF (Promega, WI) were resuspended in DMEM (200 mg/mL and 2000 ng/ml respectively) and stored at −80 °C. For the treatment of cells, IFN-γ (1 ng/ml), EGF (1000 ng/ml), both, or neither was added to cells and cellular lysates were prepared 24 h later (Qiagen, CA).

### RNA isolation and sequencing

RNA was extracted from keratinocyte samples using RNeasy Mini kit (Qiagen, CA) with DNase digest and QIAcube automation stations. The RNA was quantified, and the quality was assessed by Bioanalyzer analysis (Agilent Technologies, Santa Clara, CA). Libraries were prepared using the Clontech Smart-Seq V4 Ultra Low Input RNA kit (Takara Bio, Shiga, Japan) in combination with the Illumina NexteraXT kit (Illumina, San Diego, CA, USA), as per manufacturer’s instructions, with 10 ng of total RNA as input. The amplified libraries were validated by capillary electrophoresis on the Agilent 4200 TapeStation. The libraries were normalized, pooled, and sequenced at the Emory Integrated Genomic Core on the Illumina HiSeq3000 system employing a single-end 101 cycles run at average read depths of 20 million reads/sample. The RNA-Seq data is publicly available in the Gene Expression Omnibus database (accession number GSE279122).

### Bioinformatics analyses

Sequencing data was demultiplexed using Illumina bcl2fastq version 2.20.0.422 BCL to FastQ file converter. The quality of raw reads was assessed with FastQC version 0.11.8 [8]. Reads were mapped to the GRCh38 human genome assembly using STAR version 2.5.2b with default alignment [9]. Abundance estimation of raw read counts per transcript was done internally with STAR using the htseq-count algorithm [10].

DESeq2 version 1.22.1 R package was used to produce normalized read counts and a regularized log expression table [11]. DESeq2 was also used for computing the differential expression estimation between the treatment groups. Corresponding *P*-values were calculated using the Wald test and adjusted for multiple comparisons using the Benjamini-Hochberg method [12] to provide the false-discovery rate (FDR). Significantly differentially expressed genes (DEGs).

were identified using an FDR cutoff of 0.01 to account for the multiple treatment comparisons tested (IFN-γ vs. control, EGF vs. control, IFN-γ + EGF vs. control). Interaction *P*-values corresponding to modification of IFN-γ-induced gene expression by EGF were similarly calculated using a Wald test and adjusted for multiple comparisons; an FDR for interaction cutoff of 0.05 was used to signify statistically significant genes since only one interaction term was tested. Log-normalized expression data for a selected set of genes were visualized using heatmaps. Expression data for each gene for each sample were further normalized according to the mean expression value across all 12 samples for that gene.

Gene set enrichment analysis (GSEA) and Network Topology-based Analysis (NTA) were performed using Web Gestalt (WEB-based Gene SeT AnaLysis Toolkit, 2019) [13]. Enrichment in Gene Ontology (GO) biologic processes and Kyoto Encyclopedia of Genes and Genomes (KEGG) pathways were tested [13] using default settings and parameters in WebGestalt. Significantly enriched gene pathways were identified using an FDR cutoff of 0.05.

### In vivo RNA-seq data

Based on the in vitro RNA-seq findings, exploratory in vivo analyses were performed using publicly available, de-identified RNA-seq data (GSE117468) of psoriatic and matched normal skin punch biopsies from individuals with psoriasis enrolled in the ‘AMAGINE’ Phase III randomized clinical trials (AMAGINE-1 [Efficacy, Safety, and Withdrawal and Retreatment With Brodalumab in Moderate to Severe Plaque Psoriasis Subjects], AMAGINE-2 [P3 Study Brodalumab in Treatment of Moderate to Severe Plaque Psoriasis], and AMAGINE-3 [Efficacy and Safety of Brodalumab Compared With Placebo and Ustekinumab in Moderate to Severe Plaque Psoriasis in Subjects]). Details of the trial and subsequent RNA-seq experiments were published previously [14]. Briefly, eligible participants were adults 18 to 75 years of age with stable moderate-to-severe plaque psoriasis of at least 6 months duration. Participants were recruited between 2012 and 2015 across 155 study centers in the United States. To investigate the potential mechanistic, histologic, and transcriptional effects of treatment, punch biopsies from psoriatic (lesional) and matched normal (non-lesional) skin were obtained from a subset of participants (*n* = 116) at baseline and at 12-week follow-up and analyzed using RNA-seq. For our analyses, we used only the RNA-seq data from baseline biopsies prior to immunomodulatory treatment.

Raw RNA-seq expression data were downloaded from the NCBI Gene Expression Omnibus (GEO) database using the ‘GEOquery’ R package [15] and normalized with log2 transformation. The correlation between *EGF* expression levels (highest vs. lowest tertile expression values chosen a priori) and the expression of *IFN-γ *-regulated gene expression was investigated by quantifying the expression of individual genes and the combined expression z-scores of genes in the top enriched GO and KEGG pathways identified from our GSEA analyses. Z-scores were calculated such that all expression values were standardized to the same scale and could be combined. A positive z-score indicates above average gene expression (either for an individual gene or a combined set of genes) whereas a negative z-score indicates below average gene expression. The gene set identified as enriched for the GO:0019886 biologic process related to MHC-class II antigen presentation included *HLA-DOB*,* HLA-DMB*,* HLA-DOA*,* HLA-DPA1*,* HLA-DPA2*,* HLA-DPB1*,* HLA-DPB2*,* HLA-DQB1*,* and HLA-DQB2*. The gene set identified as enriched for the cytokine signaling KEGG:04060 pathway included *IL-6*,* GDF15*,* INHBE*,* IL-1 A*,* IL1RL1*, and *CXCL10.* All analyses, including tertile and z-score calculations, were performed separately on the normal and psoriatic skin biopsies.

All statistical tests were two-sided with a *P*-value < 0.05 considered statistically significant. Analyses were performed in R version 4.2.1 (R Foundation, Vienna, Austria).

## Results

### Transcriptomic analyses of cultured keratinocytes

Global transcriptomic data were generated using high-throughput RNA-seq of HaCaT cells 48 h after treatment with EGF, IFN-γ, both, or neither (control). On average, RNA-seq generated 19.1 million reads per sample, of which 15.0 million mapped uniquely to the human genome (Ensemble GRCh38). On average, 5% of bases mapped to intronic, 79% to exonic (51% coding, 28% UTR) and 16% to intergenic regions. Quality control (FastQC) information on the raw sequencing data for each sample is provided in Supplementary Table 1. Of the 58,050 DEGs across treatment groups, 15,311 genes had more than 10 read counts in at least one sample and were retained for further analysis.

**Fig. 1:**
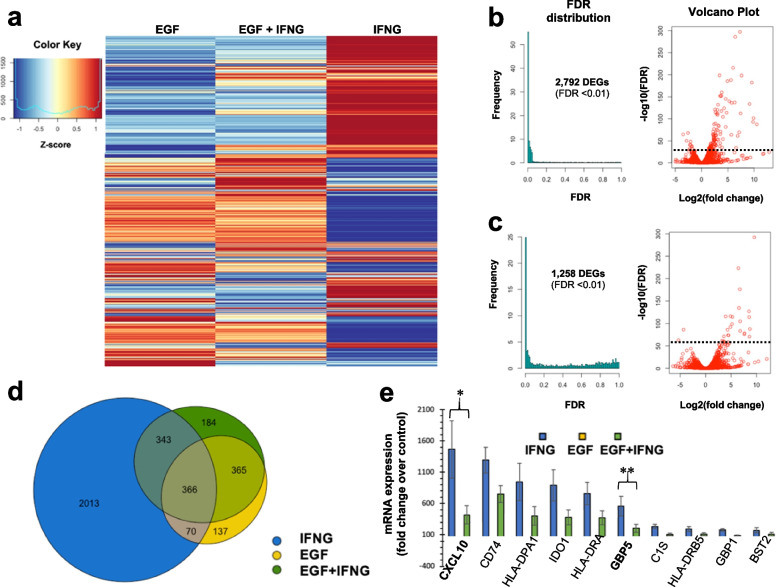
EGF modifies the IFN-g-regulated transcriptome and globally attenuates IFN-g-induced gene expression in human keratinocytes. **a** Heatmap showing differentially expressed genes (FDR < 0.01) induced by IFN-g alone, EGF alone, or IFN-g plus EGF (excluding genes that were not differentially expressed in any treatment group relative to control). FDR distributions and volcano plots (with dashed line corresponding to FDR cut-off of 0.01) showing log2 fold change in gene expression, relative to control, in keratinocytes following treatment with (**b**) IFN-g alone, and (**c**) IFN-g plus EGF. **d** Venn-diagram showing differentially expressed genes (FDR < 0.01) induced by IFN-g and/or EGF. **e** Fold change of the top 10 genes induced after treatment with IFN-g alone; asterisks above *CXCL10* and *GBP5* correspond to statistically significant interaction FDR values (*FDR < 0.05; ** FDR < 0.01) and indicate that EGF modified (attenuated) the differential gene expression induced by IFN-g (detailed information in Supplementary Table 3)

We identified 2,792 differentially expressed genes (DEGs) with IFN-γ treatment, 938 DEGs with EGF treatment, and 1,258 genes with combined EGF and IFN-γ treatment using an a priori FDR cutoff of 0.01. The transcriptomic signature of cells treated with EGF and IFN-γ together was more like that induced by EGF alone than by IFN-γ alone (Fig. 1a). A heatmap of all DEGs in each treatment group is shown in Supplementary Fig. 1. Of the 2,792 genes induced by IFN-γ treatment alone, only 709 (25%) were still induced when cells were treated with IFN-γ and EGF together (Fig. 1b-d). In contrast, 731 (78%) of the 938 genes induced by EGF alone were still significantly differentially expressed when treated with IFN-γ and EGF together. The top 40 DEGs for each treatment group are shown in Supplementary Fig. 2. The top 40 DEGs by IFN-γ included 7 HLA genes (DPA1, DRA, DRB5, DRB1, DMA, B, DQB1, and C) and all were dampened, particularly HLA-DMA, when EGF was combined with IFN-γ. *CXCL10* was the most strongly induced IFN-γ gene (1,490-fold change in expression after IFN-γ treatment) to be significantly dampened (by > 50%) after treatment with IFN-γ plus EGF (FDR for interaction = 0.01) (Fig. 1e).

### Gene-set enrichment analyses (GSEA) and gene network analyses

We identified three significantly enriched KEGG pathways (FDR < 0.05) among the DEGs with FDR values less than 0.01 when treated with IFN-γ alone but with FDR values greater than 0.10 when treated with EGF and IFN-γ (Table 1). These FDR cutoffs were chosen a priori to represent genes that may induced by IFN-γ alone but not in the presence of EGF. The complete list of DEGs is provided in Supplementary Table 2. No significantly enriched pathways were identified among the 81 EGF-induced genes (i.e., FDR < 0.01 with EGF alone) that were no longer differentially expressed in the presence of IFN-γ (i.e., FDR > 0.10 when treated with EGF and IFN-γ).

**Table 1:** Differentially expressed genes modified by IFNG alone but not in the presence of EGF clustered according to significantly enriched KEGG pathways

Gene Set	Pathway	NES	*P*	FDR	Differentially expressed genes^a^
hsa04151	*“PI3K-Akt signaling pathway”*	2.25	< 0.001	0.02	*FGF21*, ***IL6***, *ANGPT4*,* DDIT4*,* PCK2*,* IL2RG*,* CREB5*,* IRS1*,* ATF4*,* NR4A1*,* F2R*
hsa05202	*“Transcriptional misregulation in cancer”*	2.23	< 0.001	0.01	***IL6***, *DDIT3*,* GADD45A*, ***TRAF1***, ***BIRC3***, *MLLT3*,* BCL6*,* RUNX1*,* ETV6*,* GADD45B*,* KLF3*,* TFE3*,* HOXA11*,* PER2*
hs04668	*“TNF signaling pathway”*	2.08	< 0.001	0.04	***IL6***, *CXCL2*,* CREB5*, ***TRAF1***, ***BIRC3***, *ATF4*,* IL15*,* JAG1*,* RELA*,* Table 2*,* JUN*,* TRAF5*,* RPS6KA5*,* EDN1*

***Bolded*** type indicates common genes in multiple enriched pathways. Log2fold changes in gene expression and corresponding *P*-values for all IFNG-regulated genes (FDR < 0.01) that were not differentially expressed in the presence of EGF (FDR > 0.10) are presented in Supplementary Table 2

^a^Listed in order of their score within the gene set enrichment analyses

**Fig. 2:**
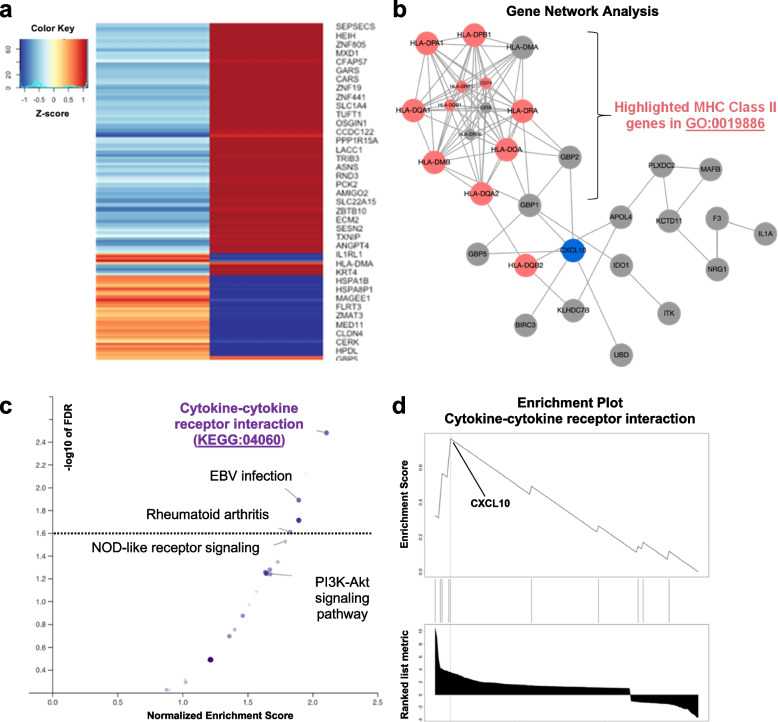
IFN-g-regulated genes and associated pathways most affected by EGF in keratinocytes. **a** Heatmap showing log2 fold change in gene expression of IFN-g-regulated genes whose expression was either significantly dampened or augmented by EGF (*N* = 160, interaction FDR < 0.05). Note that not all genes fit on the axis to the right of the heatmap but are shown in Supplementary Table 3 with detailed data. **b** Gene network from Network Topology Analysis (NTA) of IFN-g-regulated genes whose expression was either 2-fold higher or lower when EGF was added; genes in the top enriched GO Biological Process category are shaded in red (‘Antigen processing and presentation of exogenous peptide antigen via MHC class II’ [GO:0019886]; FDR = 2.65 × 10^−7^); blue shading of *CXCL10* denoting it as the most strongly upregulated gene by IFN-g to be dampened by EGF treatment. **c** Volcano plot showing the enriched KEGG pathways within the list of IFN-g-induced genes to be significantly dampened or augmented (interaction FDR < 0.05) by EGF treatment. Dashed line represents statistical significance cutoff of FDR 0.05. **d** Enrichment plot of the most significantly enriched KEGG pathway (KEGG:04060): ‘Cytokine-cytokine receptor interaction’ (NES = 2.11, *P* < 0.0001, FDR = 0.003); *CXCL10* had the highest ranked list metric (10.5) identifying it as the top gene driving this enrichment signal

We identified 160 IFN-γ-induced DEGs that were significantly modified by EGF (FDR for interaction < 0.05), as shown in the heatmap in Fig. 2a and in Supplementary Table 3. The differential gene expression after treatment with IFN-γ plus EGF was either significantly higher (*n* = 119) or significantly lower (*n* = 41) than that expected based on their differential expression after treatment with IFN-γ or EGF alone (Fig. 2). The most significantly enriched KEGG pathway within this list of DEGs was ‘Cytokine-cytokine receptor interaction’ (NES = 2.11, *P* < 0.0001, FDR = 0.003). In the top enriched cytokine KEGG pathway, *CXCL10* had the highest ranked list metric (10.52), identifying it as the top gene within the lead-edge subset of genes responsible for driving the enrichment signal (Fig. 2d). The enriched KEGG pathways (as shown in Fig. 2c) and their associated NES and FDR values are presented in Supplementary Table 4. For IFN-γ-induced genes with expression that was either 2-fold lower or 2-fold higher with EGF, the top enriched GO biologic process involved MHC class II antigen presentation (GO:0019886; FDR = 2.65 × 10^−7^) driven by expression changes in several HLA-D genes (Fig. 2b).

EGF-induced DEGs (*n* = 938) were enriched in biologic processes including “tissue development”, “cell migration”, and “epithelium development”, consistent with established roles of EGF in the skin (Supplementary Table 5). There were no enriched GO biologic processes or KEGG pathways identified among the small subset EGF-induced DEGs whose expression was 2-fold lower in the presence of IFN-γ (*n* = 26). However, among the small number of EGF-induced DEGs whose expression was 2-fold higher in the presence of IFN-γ (*n* = 45), enriched biologic processes included “interspecies interaction between organisms” (GO:0044419), “immune response” (GO:0006955), and “response to external stimulus” (GO:0009605) (Supplementary Table 5). The gene network from Network Topology Analysis (NTA) for these EGF-induced genes in the top enriched GO process (GO:0044419) is presented in Supplementary Fig. 6 (analogous to that which is presented for IFN-γ induced genes in Fig. 2).

### EGF expression modifies IFN-γ target genes in human skin

Based on our in vitro results in keratinocytes, we sought to investigate whether higher *EGF* expression modified the association of IFN-γ*-*regulated gene expression in the skin in vivo. We used RNA-seq data of biopsies of psoriatic and matched normal skin collected from 116 individuals with psoriasis enrolled in the AMAGINE Phase III randomized clinical trials. We chose to investigate the correlation of IFN-γ with: (i) CXCL10 and GBP5, as they were the most strongly IFN-γ-induced DEGs to be modified by EGF in vitro (as shown in Fig. 1e) and (ii) the combined expression z-score of genes identified in the top enriched GO and KEGG pathways related to MHC-class II antigen presentation (GO:0019886) and cytokine signaling (KEGG:04060) (as shown in Fig. 2).

**Fig. 3:**
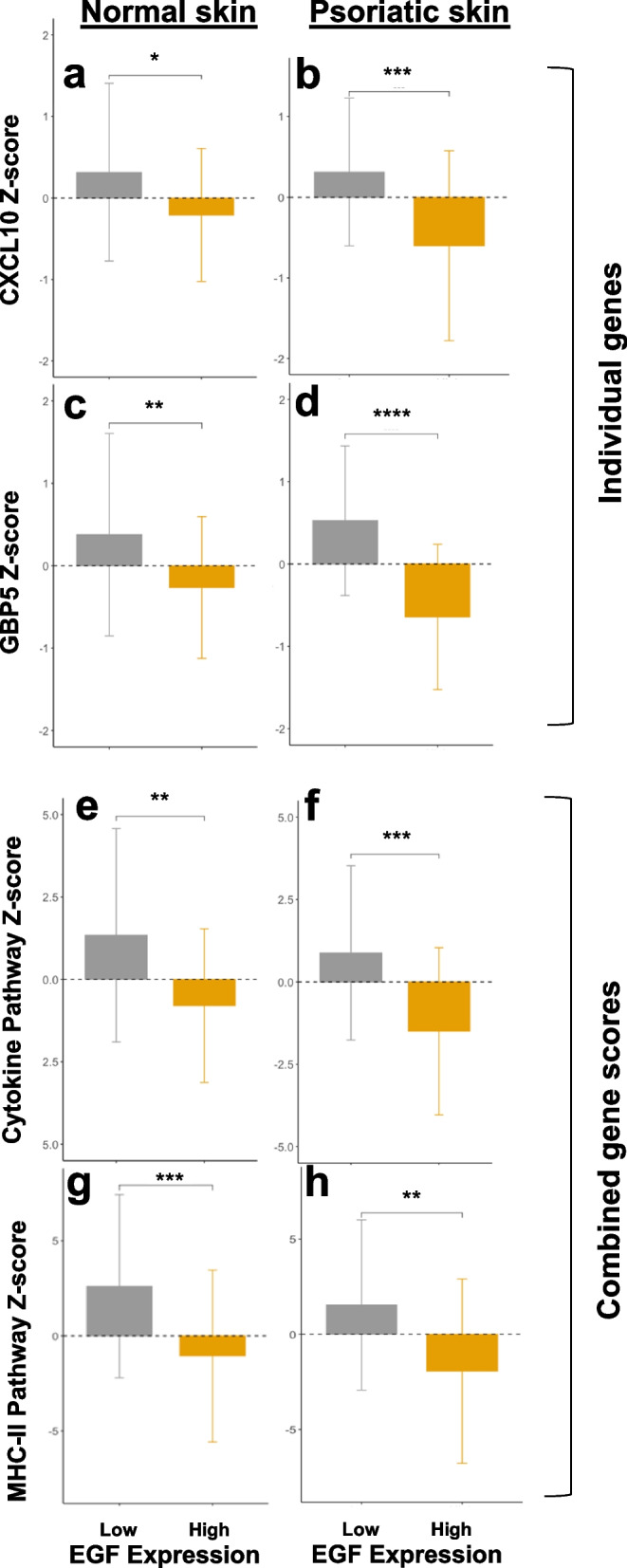
*EGF* expression associated with lower *CXCL10*,* GBP5*, MHC-class II and cytokine signaling pathway gene expression in psoriatic and matched normal skin. Gene expression Z-scores were calculated from RNA-seq data of skin biopsies collected from 116 individuals with psoriasis enrolled in the AMAGINE Phase III randomized clinical trials. Gene expression Z-scores of psoriatic lesions and matched normal (non-lesional) skin at low and high EGF expression tertile for (**a-b**) CXCL10, (**c-d**) GBP5, (**e-f**) combined expression of genes in cytokine signaling pathway (KEGG:04060), and (**g-h**) combined expression of genes in MHC-class II antigen presentation (GO:0019886). (Bonferroni-adjusted *P*-values: * < 0.05; ** < 0.01; *** <0.001; ****<0.0001)

**Fig. 4:**
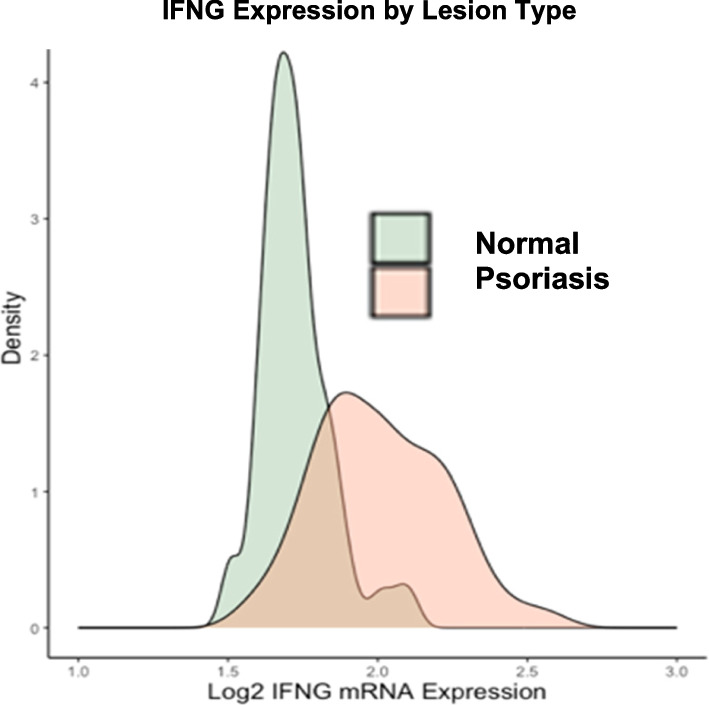
Psoriatic skin exhibits overall higher *IFNG* gene expression compared to matched normal skin. Density plot of log2 *IFNG* mRNA expression distribution in skin biopsies from psoriatic lesions and matched normal (non-lesional) skin biopsies collected from 116 individuals with psoriasis enrolled in the AMAGINE Phase III randomized clinical trials

In both normal and psoriatic skin biopsies, higher *EGF* expression tertile was significantly associated with lower *CXCL10* and *GBP5* gene expression (Fig. 3a-d). Similarly, the combined expression scores of MHC-class II and cytokine signaling pathway genes were lower in skin samples with higher *EGF* expression (Fig. 3e-h). These associations tended to be stronger in psoriatic skin biopsies, which, as expected, had higher overall *IFNG* expression compared to matched normal skin (Fig. 4). The Pearson correlation between *IFNG* gene expression and target *CXCL10*,* GBP5*, MHC-class II, and cytokine signaling pathway gene expression scores according to *EGF* expression are shown in Supplementary Fig. 3. Higher *IFNG* gene expression was significantly associated with *CXCL10*,* GBP5*, MHC-class II, and cytokine signaling pathway gene expression scores in samples with lower *EGF* expression; however, the corresponding associations tended to be weaker in samples with higher *EGF* expression. This pattern of effect modification was more prominent in normal skin biopsies, which may correspond more closely with our in vitro model of normal keratinocytes (Supplementary Fig. 3).

## Discussion

Our findings suggest that EGF may globally attenuate pro-inflammatory gene expression induced by IFN-γ in human keratinocyte skin cells. IFN-γ can drive cutaneous inflammation and immune responses by regulating the expression of MHC molecules involved in antigen presentation and adaptive immune responses, including anti-tumor immune responses [16]. We found that EGF attenuated the IFN-γ-induced upregulation of several MHC-class II genes encoding HLA-DP, HLA-DM, HLA-DOA, HLA-DOB, and HLA-DQ. Using gene set and network enrichment analyses, we identified several IFN-γ-induced biologic pathways that may be dampened by EGF in keratinocytes. As subsequently discussed, our findings are impactful as they: (i) further elucidate the transcriptional crosstalk between EGF and IFN-γ in keratinocytes using a genome-wide approach, (ii) extend previous work of immune-related genes dysregulated by EGFR inhibitors, and (iii) suggest that EGF may impact gene expression and pathways associated with cutaneous inflammation.

Prior studies suggest that EGF may have downstream immunomodulatory effects by reducing the expression of specific genes, including HLA-DR and ICAM-1 [5]. While previous studies focused on a handful of pre-specified genes, this is the first study, to our knowledge, to investigate EGF effects on interferon transcriptional regulation on a genome-wide scale. This approach may help identify novel genes regulated by EGF and/or IFN-γ as well as elucidate broader transcriptional networks by using gene set and bioinformatic analyses. In addition to the attenuation of specific HLA genes by EGF in our in vitro keratinocyte model, our results suggest that EGF may globally attenuate the IFN-γ-induced transcriptome since most genes differentially expressed after IFN-γ treatment alone were no longer differentially expressed when treated with both IFN-γ and EGF. Additionally, we identified 160 IFN-γ-induced DEGs that were significantly modified by EGF, including IL-6, IL-1 A, HLA-DMA, and CXCL10, which are enriched in cytokine signaling and antigen presenting pathways consistent with known pro-inflammatory IFN-γ functions. Interestingly, other IFN-γ-induced KEGG pathways significantly attenuated by EGF included rheumatoid arthritis’ (FDR = 0.02) and ‘Epstein-Barr virus infection’ (FDR = 0.01). These findings support the notion that EGF’s immunomodulatory properties may be a “double-edged sword” — dampening both harmful autoimmune inflammation as well as helpful anti-pathogen immune responses.

Our findings also indicate that IFN-g may impact EGFR-mediated gene expression. However, the impact of IFN-g on the EGF-induced transcriptome appeared to be less than that of EGF on the IFN-g-induced transcriptome. For example, while 75% of the IFN-g-induced genes were no longer differentially expressed in the presence of EGF, only 22% of EGF-induced genes were no longer differentially expressed in the presence of IFN-g. Interestingly, IFN-g did significantly increase the expression of a small subset (*n* = 45) of EGF-induced genes, which were enriched in several GO biologic processes including “immune response”, “response to external stimulus”, and “defense response to other organisms” (Supplementary Table 5). This corroborates prior studies which indicate a complex crosstalk between the IFN-g receptor complex and EGFR-mediated signaling through direct and indirect mechanisms [6, 16]. Briefly, the IFN-g complex can induce shedding of membrane-bound EGFR ligands leading to receptor phosphorylation and EGFR activation [6, 16]. This in turn can down-regulate key immunomodulatory genes (e.g.,, MHC class I and II expression) induced by interferon in the skin (as reviewed in [16]). Also, IFN-g STAT1 activation in keratinocyte cell lines may partially depend on EGFR kinase activity [7]. Our findings suggest that the crosstalk between IFN-g and EGF/EGFR may be one-sided, at least on the genome-wide transcriptomic level, in the sense that EGF modified the IFN-g transcriptome more than IFN-g modified the EGF transcriptome in our keratinocyte model. Additional studies are needed to elucidate the molecular mechanisms involved in this crosstalk which we hypothesize may act as an important counterbalancing system between pro and anti-inflammatory gene expression in the skin.

These findings expand upon previous work investigating the immunomodulatory properties of EGFR inhibitors (EGFRIs). We previously reported that MHC class I and II induction by IFN-γ was increased by EGFRI in keratinocytes [6]. EGFRIs may also upregulate several inflammatory cytokines/chemokines including CCL2, CCL5, TNF-alpha, IL1-b, and CXCL10 [17–20]. These findings support the increased immune cell recruitment and inflammatory signatures observed in skin biopsies of EGFRI-treated cancer patients [6]. However, it is unclear whether this is causative or reactive, since some hypothesize that EGFR blockade results in dysregulation of homeostatic functions in keratinocytes, precipitating a reactive inflammatory response [4, 18, 21]. Understanding the immunomodulatory effects of EGF is also important since it may be used as a topical agent to reduce acneiform eruptions in patients taking EGFRIs [22, 23]. Topical EGF improved inflammatory acne in a randomized clinical trial [24] and enhanced barrier function to improve dermatitis phenotypes in mice [25]. The potential therapeutic benefits of EGF must be weighed against its theoretical pro-carcinogenic effects, although there is no clear evidence suggesting that topical EGF increases cancer risk [26].

Based on our in vitro results, we further investigated whether EGF may modify IFN-γ target gene expression in vivo using RNA-seq data of skin samples from individuals with psoriasis. Our findings suggest that the expression of certain IFN-γ target genes, such as *CXCL10* and *GBP5*, may be dampened by higher EGF in the skin. CXCL10 (interferon-γ-inducible protein 10, previously called IP-10) is an IFN-γ-induced chemokine that has a well-established role in the recruitment of various immune cells, including natural killer cells, monocytes, neutrophils, and T lymphocytes [27–29]. CXCL10 appears to be important for leukocyte trafficking that leads to increased tissue inflammation and may be overexpressed in CD4 + T helper 1 (Th1)-mediated inflammatory diseases like multiple sclerosis and psoriasis [27–29]. GBP5 (guanylate binding protein 5) plays an important role in the assembly of the NLRP3 inflammasome in response to infection and a variety of inflammatory disease processes [15]. We suspect that the upregulation of these genes as well as other genes involved in MHC-class II antigen presentation and inflammatory cytokine signaling upregulated by IFN-γ may be attenuated by higher EGF expression in the skin. This is particularly relevant since there is growing interest in topical EGF as a potential treatment for EGFRI-induced skin inflammation [30] and other inflammatory skin diseases like acne vulgaris [24]. The extent to which these effects are driven by dampening of the pro-inflammatory IFN-γ-induced transcriptome deserve further study.

Our study has several limitations. First, the in vitro keratinocyte model does not reflect the multicellular immunological interactions in the skin influenced by EGF and IFN-γ signaling. We recognize that changes in mRNA regulation do not necessarily translate to biologically meaningful alternations in protein levels and/or cellular phenotypes, which could be a direction for future studies. Additionally, the DEGs identified using RNA-seq were unverified by additional analysis such as real-time PCR. However, we chose to use a more stringent FDR threshold to define DEGs, and our gene set enrichment analyses of DEGs induced by IFN-γ or EGF alone were consistent with known IFN-γ and EGF functions, supporting the validity of our transcriptome findings. Lastly, the analyses of RNA-seq data from skin samples taken from psoriasis patients in the AMAGINE trials were not adjusted for multiple comparisons; these analyses were considered exploratory and should be interpreted cautiously.

In summary, our findings suggest that EGF may significantly dampen the IFN-γ-induced transcriptome in keratinocytes, including genes involved in leukocyte trafficking, antigen presentation, and tissue inflammation upregulated in autoimmune diseases like psoriasis and by EGFR inhibitors. These findings complement the work by others to expand our understanding of the non-canonical immunomodulatory role of EGF signaling in the skin. Additional studies are needed to define how EGF signaling influences downstream antigen presentation and immune cell recruitment in normal and inflamed skin and among individuals treated with EGFRIs who are commonly affected by inflammatory skin reactions.

## Supplementary Information

Supplementary Material 1.

## Data Availability

The dataset(s) supporting the conclusions of this article is (are) available in the Gene Expression Omnibus (GEO) repository (GSE279122, GSE117468) and upon reasonable request to the Corresponding Author.

## Abbreviations

EGF: Epidermal Growth Factor
EGFR: Epidermal Growth Factor Receptor
EGFR-I: Epidermal Growth Factor Receptor Inhibitor
IGN-γ: Interferon-γ
HaCaT: Human keratinocyte
FDR: False discovery rate
MHC: Major Histocompatibility Complex
FBS: Fetal Bovine Serum
DEG: Differentially expressed genes
GSEA: Gene set enrichment analysis
NTA: Network Topology-based Analysis
GO: Gene Ontology
KEGG: Kyoto Encyclopedia of Genes and Genomes
