# Correction: Epidermal growth factor dampens pro-inflammatory gene expression induced by interferon-gamma in global transcriptome analysis of keratinocytes

**DOI:** 10.1186/s12864-025-11457-5

**Published:** 2025-03-24

**Authors:** David C. Gibbs, Myles R. McCrary, Carlos S. Moreno, Lindsey Seldin, Chaoran Li, Nourine A. H. Kamili, Brian P. Pollack

**Affiliations:** 1 Atlanta Veterans Affairs Medical Center, Decatur, GA 30033 USA; 2 Department of Dermatology, Emory University School of Medicine, Atlanta, GA 30322 USA; 3 Dermatology and Cutaneous Surgery, Morsani College of Medicine, University of South Florida, Tampa, FL 33612 USA; 4 Department of Anatomic and Clinical Pathology, University of South Florida, Tampa, FL 33612 USA; 5 Department of Pathology and Laboratory Medicine, Emory University School of Medicine, Atlanta, GA 30322 USA; 6 Department of Cell Biology, Emory University, Atlanta, GA 30322 USA; 7 Department of Microbiology and Immunology, Emory University School of Medicine, Atlanta, GA 30322 USA; 8 Winship Cancer Institute, Emory University School of Medicine, Atlanta, GA 30322 USA

**Correction**: ***BMC Genomics*26**,** 122 (2025).**10.1186/s12864-025-11237-1

Following publication of the original article, it was reported that there was an error in the affiliation assignment in the authorship panel. The incorrect and correct affiliations are given below.

The incorrect affiliations were:

David C. Gibbs^5^, Myles R. McCrary^2,6^, Carlos S. Moreno^3^, Lindsey Seldin^1,4,5,8^, Chaoran Li^5,7,8^, Nourine A. H. Kamili^5^ and Brian P. Pollack^1,5,8^.

1 Atlanta Veterans Afairs Medical Center, Decatur, GA 30,033, USA.

2 Department of Dermatology, Emory University School of Medicine, Atlanta, GA 30,322, USA.

3 Dermatology and Cutaneous Surgery, Morsani College of Medicine, University of South Florida, Tampa, FL 33,612, USA.

4 Department of Anatomic and Clinical Pathology, University of South Florida, Tampa, FL 33,612, USA.

5 Department of Pathology and Laboratory Medicine, Emory University School of Medicine, Atlanta, GA 30,322, USA.

6 Department of Cell Biology, Emory University, Atlanta, GA 30,322, USA.

7 Department of Microbiology and Immunology, Emory University School of Medicine, Atlanta, GA 30,322, USA.

8 Winship Cancer Institute, Emory University School of Medicine, Atlanta, GA 30,322, USA.

The correct affiliations are:

David C. Gibbs ^2^, Myles R. McCrary ^3,4^, Carlos S. Moreno ^5,8^, Lindsey Seldin ^1,2,6,8^, Chaoran Li ^2,7,8^, Nourine A.H. Kamili ^2^, Brian P. Pollack ^1,2,5,8^.

1 Atlanta Veterans Affairs Medical Center, Decatur GA 30,033, USA.

2 Department of Dermatology, Emory University School of Medicine, Atlanta, GA 30,322, USA.

3 Dermatology and Cutaneous Surgery, Morsani College of Medicine, University of South Florida, Tampa, FL 33,612, USA.

4 Department of Anatomic and Clinical Pathology, University of South Florida, Tampa, FL 33,612, USA.

5 Department of Pathology and Laboratory Medicine, Emory University School of Medicine, Atlanta, GA 30,322, USA.

6 Department of Cell Biology, Emory University, Atlanta, GA 30,322, USA.

7 Department of Microbiology and Immunology, Emory University School of Medicine, Atlanta, GA 30,322, USA.

8 Winship Cancer Institute, Emory University School of Medicine, Atlanta, GA 30,322, USA.

The original article has been updated.

