# Supplementary material for: Epidermal growth factor dampens pro-inflammatory gene expression induced by interferon-gamma in global transcriptome analysis of keratinocytes
Source: BMC Genomics. 2025 Feb 10;26:122. doi: 10.1186/s12864-025-11237-1 (PMC11809098; doi:10.1186/s12864-025-11237-1)
Supplement: Supplementary file 1 — Supplementary Material 1. [file 12864_2025_11237_MOESM1_ESM.docx]

| **Supplementary Table 1.** FastQC quality control results of raw sequence data for all samples. | | | | | |
| --- | --- | --- | --- | --- | --- |
| **Sample** | **Total_Sequences** | **Sequences>Q30** | **%Percent>Q30** | **Sequences>Q20** | **Percent>Q20** |
| 1_untreated_cells_R1_S174_L005_R1_001.fastq.gz | 14575508 | 14363505 | 98.5 | 14575318 | 100 |
| 1_untreated_cells_R1_S174_L006_R1_001.fastq.gz | 5236917 | 5155896 | 98.5 | 5236846 | 100 |
| 2_EGF_treated_cells_R1_S175_L005_R1_001.fastq.gz | 12167310 | 11994390 | 98.6 | 12167219 | 100 |
| 2_EGF_treated_cells_R1_S175_L006_R1_001.fastq.gz | 4430572 | 4363650 | 98.5 | 4430538 | 100 |
| 3_IFN-gamma_treated_cell_R1_S176_L005_R1_001.fastq.gz | 10546779 | 10393646 | 98.5 | 10546691 | 100 |
| 3_IFN-gamma_treated_cell_R1_S176_L006_R1_001.fastq.gz | 3770752 | 3712844 | 98.5 | 3770717 | 100 |
| 4_EGF_IFN-gamma_treated_cells_R1_S177_L005_R1_001.fastq.gz | 11376404 | 11210013 | 98.5 | 11376289 | 100 |
| 4_EGF_IFN-gamma_treated_cells_R1_S177_L006_R1_001.fastq.gz | 4239879 | 4174945 | 98.5 | 4239827 | 100 |
| 5_untreated_cells_R2_S178_L005_R1_001.fastq.gz | 14876108 | 14692930 | 98.8 | 14876018 | 100 |
| 5_untreated_cells_R2_S178_L006_R1_001.fastq.gz | 5347041 | 5276073 | 98.7 | 5347011 | 100 |
| 6_EGF_treated_cells_R2_S179_L005_R1_001.fastq.gz | 15165119 | 14981804 | 98.8 | 15165018 | 100 |
| 6_EGF_treated_cells_R2_S179_L006_R1_001.fastq.gz | 5469199 | 5398324 | 98.7 | 5469160 | 100 |
| 7_IFN-gamma_treated_cell_R2_S180_L005_R1_001.fastq.gz | 14545012 | 14351375 | 98.7 | 14544859 | 100 |
| 7_IFN-gamma_treated_cell_R2_S180_L006_R1_001.fastq.gz | 5149680 | 5075166 | 98.6 | 5149627 | 100 |
| 8_EGF_IFN-gamma_treated_cells_R2_S181_L005_R1_001.fastq.gz | 15384729 | 15180438 | 98.7 | 15384621 | 100 |
| 8_EGF_IFN-gamma_treated_cells_R2_S181_L006_R1_001.fastq.gz | 5459416 | 5380348 | 98.6 | 5459366 | 100 |
| 9_untreated_cells_R3_S182_L005_R1_001.fastq.gz | 16243097 | 16034242 | 98.7 | 16242936 | 100 |
| 9_untreated_cells_R3_S182_L006_R1_001.fastq.gz | 5795668 | 5714591 | 98.6 | 5795584 | 100 |
| 10_EGF_treated_cells_R3_S183_L005_R1_001.fastq.gz | 15367614 | 15165427 | 98.7 | 15367511 | 100 |
| 10_EGF_treated_cells_R3_S183_L006_R1_001.fastq.gz | 5480503 | 5401702 | 98.6 | 5480455 | 100 |
| 11_IFN-gamma_treated_cell_R3_S184_L005_R1_001.fastq.gz | 13435489 | 13270345 | 98.8 | 13435393 | 100 |
| 11_IFN-gamma_treated_cell_R3_S184_L006_R1_001.fastq.gz | 4739221 | 4676020 | 98.7 | 4739179 | 100 |
| 12_EGF_IFN-gamma_treated_cells_R3_S185_L005_R1_001.fastq.gz | 14741062 | 14588949 | 99 | 14741009 | 100 |
| 12_EGF_IFN-gamma_treated_cells_R3_S185_L006_R1_001.fastq.gz | 5358037 | 5297757 | 98.9 | 5358018 | 100 |

**Supplementary Figure 1.** Heatmap of differential gene expression of all genes according to treatment group


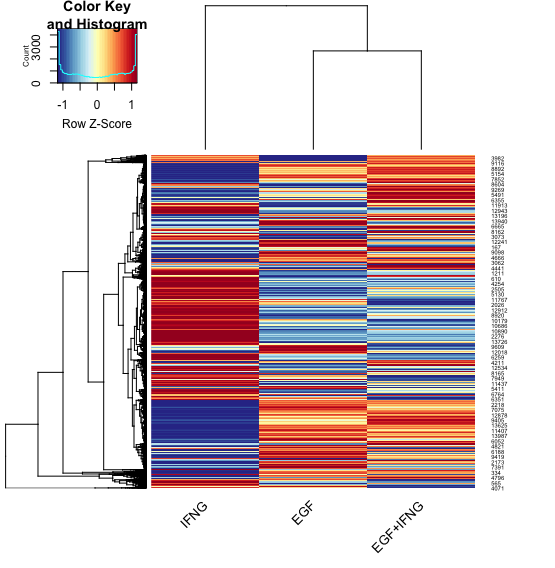


**Supplementary Figure 2**. Bar chart showing log2fold changes of the top 40 genes induced by IFN-γ, EGF, and IFN-γ plus EGF

**Supplementary Figure 4.** Correlation between *IFNG* and target gene expression according to *EGF* expression (lowest and highest tertiles) in psoriatic and matched normal skin


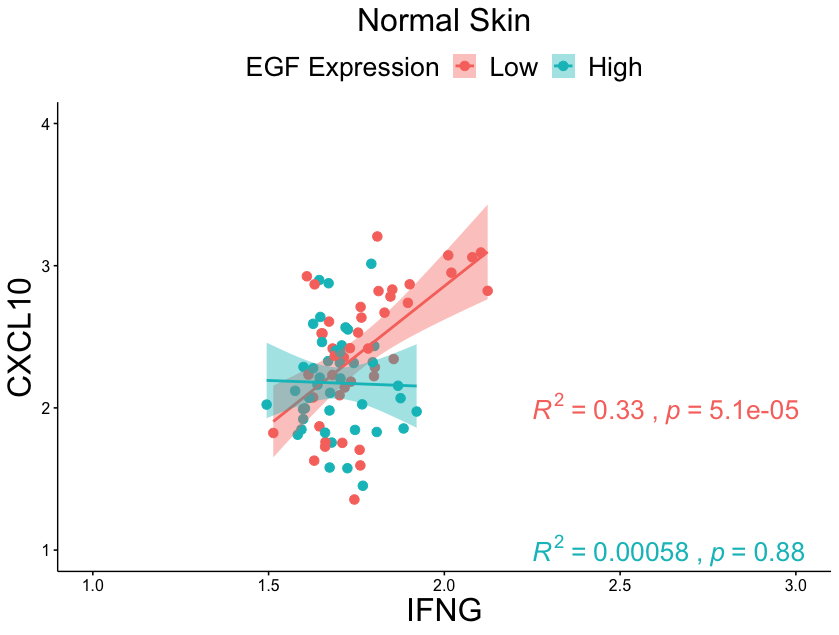

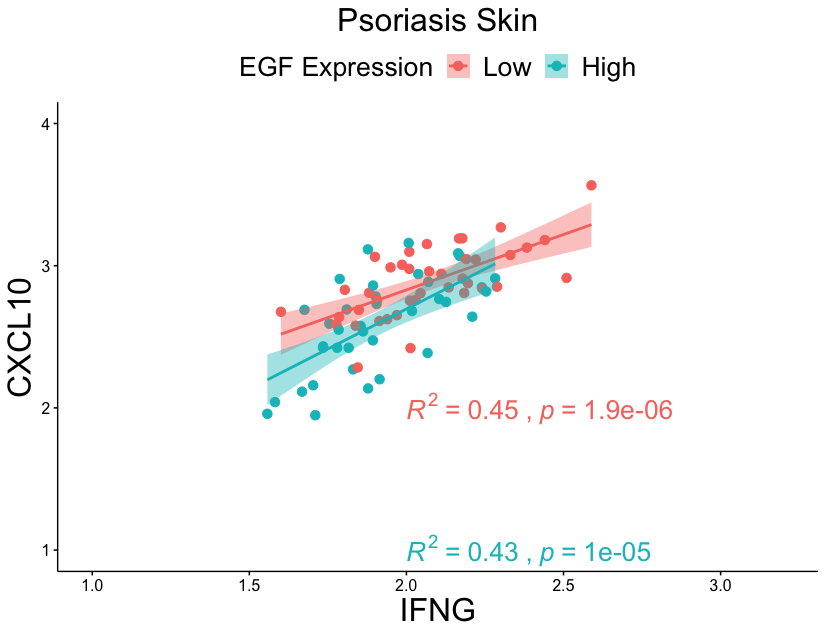


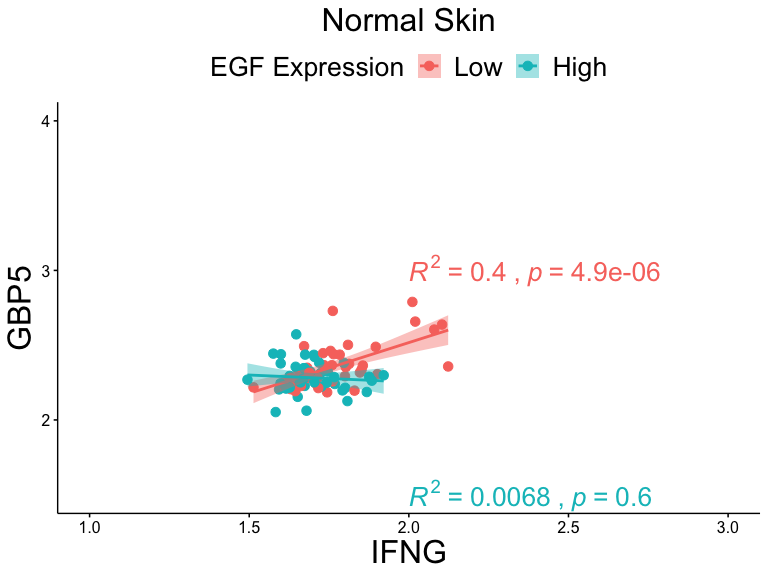

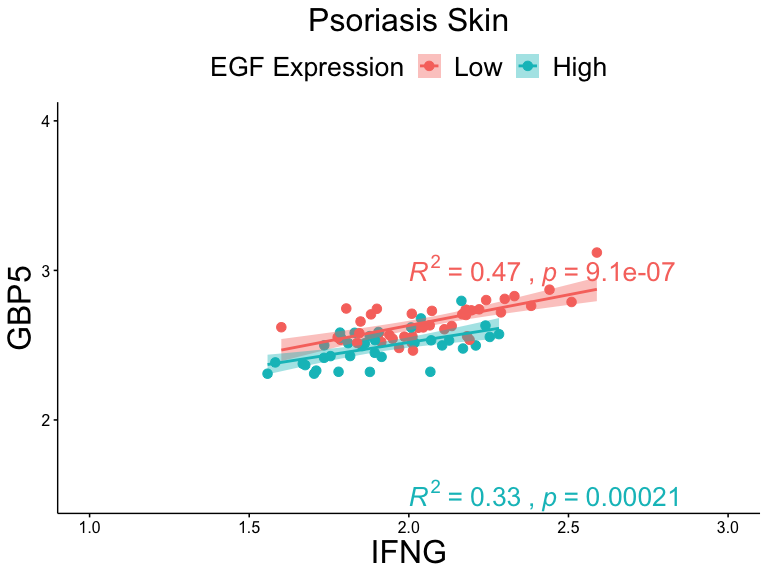


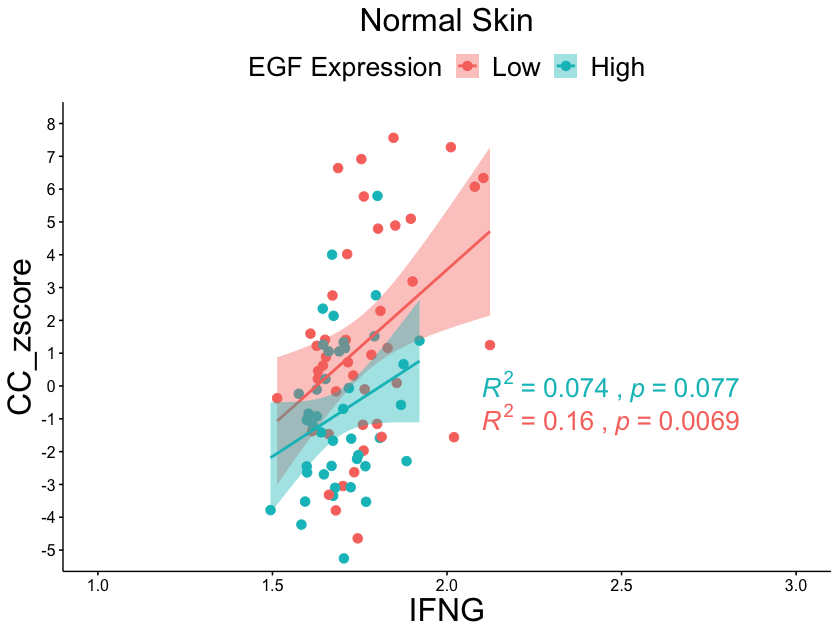

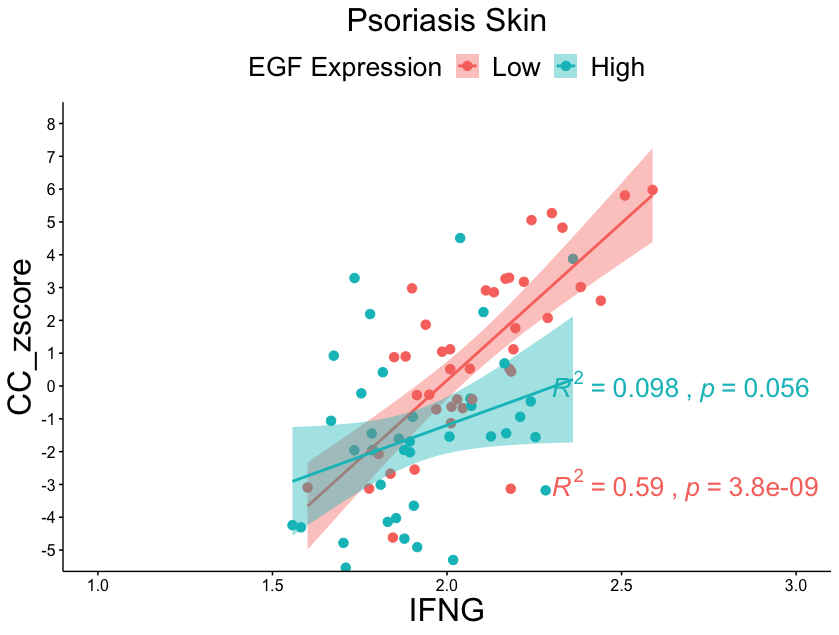


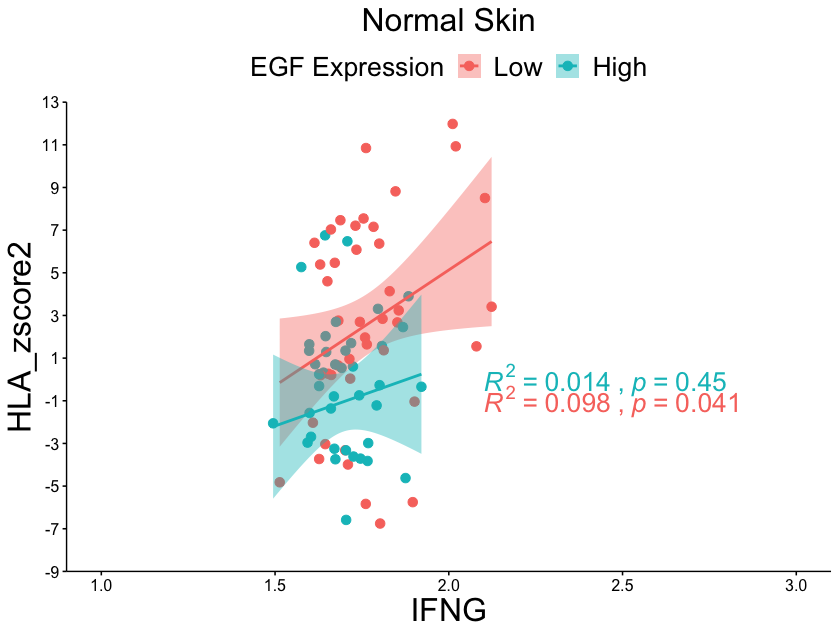

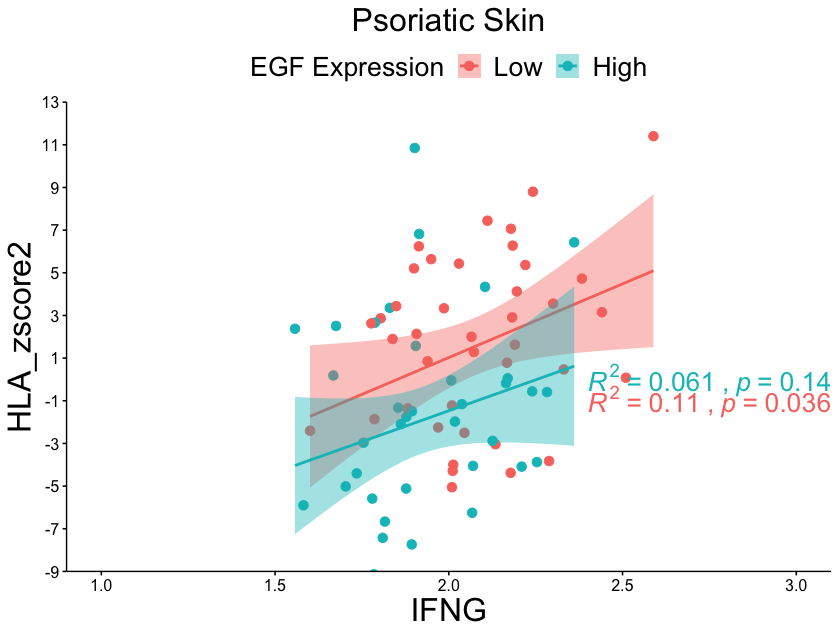


| **Supplementary Table 2.** List of 1,764 DEGs identified after IFN-γ treatment alone (FDR < 0.01) that were not significantly differentially expressed (FDR > 0.10) when treated with IFN-γ plus EGF | | | | | | | | | | | | | | | | | | | | | |
| --- | --- | --- | --- | --- | --- | --- | --- | --- | --- | --- | --- | --- | --- | --- | --- | --- | --- | --- | --- | --- | --- |
| **Gene** | **IFNG** | | **IFNG *P_adjusted_*** | **EGF** | **EGF *P_adjusted_*** | | **IFNG+EGF** | **IFNG+EGF *P_adjusted_*** | |  | | **Gene** | **IFNG** | **IFNG *P_adjusted_*** | | **EGF** | | **EGF *P_adjusted_*** | **IFNG+EGF** | **IFNG+EGF *P_adjusted_*** | |
| C4B | 6.83 | | 2.49E-06 | NA | NA | | 2.33 | 7.42E-01 | |  | | SMG5 | 0.52 | 3.17E-04 | | -0.07 | | 8.90E-01 | 0.13 | 7.75E-01 | |
| FGF21 | 5.21 | | 4.97E-03 | NA | NA | | 1.31 | 9.18E-01 | |  | | BBX | 0.52 | 2.48E-04 | | 0.10 | | 7.98E-01 | 0.21 | 5.54E-01 | |
| RAB39B | 4.81 | | 2.37E-07 | 0.74 | 8.82E-01 | | 1.63 | 5.47E-01 | |  | | KANSL3 | 0.51 | 1.58E-03 | | -0.08 | | 8.76E-01 | 0.18 | 6.49E-01 | |
| GDF15 | 4.17 | | 1.65E-21 | 1.29 | 1.37E-01 | | -0.07 | 9.76E-01 | |  | | GOSR1 | 0.51 | 1.02E-04 | | 0.14 | | 6.95E-01 | 0.12 | 7.80E-01 | |
| ITK | 4.16 | | 4.86E-03 | 0.19 | 9.82E-01 | | 2.42 | 4.33E-01 | |  | | MED13 | 0.51 | 6.16E-03 | | -0.10 | | 8.53E-01 | 0.08 | 9.05E-01 | |
| INHBE | 4.14 | | 2.46E-10 | -0.42 | 9.29E-01 | | -1.98 | 5.14E-01 | |  | | SIPA1L1 | 0.51 | 5.46E-03 | | 0.06 | | 9.29E-01 | 0.31 | 4.05E-01 | |
| ADM2 | 3.88 | | 8.47E-36 | 0.93 | 2.17E-01 | | 0.63 | 4.96E-01 | |  | | ZNF800 | 0.51 | 3.42E-03 | | 0.01 | | 9.83E-01 | 0.08 | 8.88E-01 | |
| CHAC1 | 3.82 | | 8.37E-161 | 0.00 | 9.98E-01 | | 0.49 | 1.82E-01 | |  | | TFAP2C | 0.51 | 2.46E-04 | | 0.14 | | 7.38E-01 | 0.14 | 7.46E-01 | |
| IL6 | 3.60 | | 1.27E-71 | -0.40 | 6.34E-01 | | 0.66 | 1.54E-01 | |  | | HARBI1 | 0.51 | 8.93E-03 | | 0.09 | | 8.85E-01 | 0.02 | 9.73E-01 | |
| ANGPT4 | 3.39 | | 4.12E-12 | 1.41 | 1.80E-01 | | 0.91 | 5.01E-01 | |  | | SAP30L | 0.51 | 3.45E-03 | | -0.03 | | 9.71E-01 | 0.14 | 7.70E-01 | |
| KIF21B | 3.37 | | 1.42E-12 | 1.68 | 1.91E-02 | | 1.10 | 2.75E-01 | |  | | TRMT5 | 0.51 | 1.79E-03 | | 0.06 | | 9.25E-01 | 0.12 | 7.91E-01 | |
| TCIM | 3.26 | | 1.34E-04 | -0.64 | 8.96E-01 | | -0.19 | 9.63E-01 | |  | | MAPK6 | 0.51 | 7.48E-03 | | -0.40 | | 1.21E-01 | -0.36 | 4.04E-01 | |
| ADGRA2 | 3.25 | | 9.24E-06 | 0.63 | 8.21E-01 | | 0.51 | 8.51E-01 | |  | | CKMT1B | 0.51 | 1.20E-03 | | 0.09 | | 8.61E-01 | 0.37 | 1.89E-01 | |
| VLDLR | 3.23 | | 1.78E-05 | 0.37 | 9.30E-01 | | 1.13 | 5.86E-01 | |  | | FTL | 0.51 | 1.35E-03 | | 0.16 | | 7.45E-01 | 0.48 | 1.36E-01 | |
| ATF3 | 3.21 | | 8.63E-10 | -0.70 | 2.50E-01 | | 0.33 | 7.01E-01 | |  | | NEPRO | 0.51 | 6.62E-04 | | -0.11 | | 8.48E-01 | -0.18 | 7.32E-01 | |
| ISCA1P4 | 3.08 | | 4.30E-03 | -1.21 | 8.32E-01 | | 0.26 | 9.57E-01 | |  | | IPPK | 0.50 | 6.03E-03 | | -0.03 | | 9.67E-01 | 0.10 | 8.65E-01 | |
| ULBP1 | 3.06 | | 3.00E-17 | -0.50 | 7.42E-01 | | -0.83 | 4.87E-01 | |  | | DCAF10 | 0.50 | 3.95E-03 | | -0.26 | | 4.16E-01 | -0.30 | 4.25E-01 | |
| SESN2 | 3.02 | | 9.19E-75 | -0.06 | 9.44E-01 | | 0.26 | 5.44E-01 | |  | | GOLGA5 | 0.50 | 1.90E-03 | | -0.04 | | 9.59E-01 | 0.17 | 7.25E-01 | |
| STC2 | 2.86 | | 1.16E-76 | 0.48 | 9.52E-03 | | 0.39 | 1.44E-01 | |  | | ZNF37A | 0.50 | 1.69E-03 | | 0.02 | | 9.71E-01 | -0.09 | 8.89E-01 | |
| UNC13A | 2.75 | | 5.66E-03 | 0.52 | 8.99E-01 | | 1.90 | 2.15E-01 | |  | | TRIM26 | 0.50 | 3.41E-03 | | -0.17 | | 7.04E-01 | 0.40 | 1.96E-01 | |
| CLIC2 | 2.68 | | 6.08E-03 | -0.33 | 9.47E-01 | | 1.18 | 5.96E-01 | |  | | NISCH | 0.50 | 8.27E-03 | | 0.06 | | 9.35E-01 | 0.18 | 7.18E-01 | |
| TREML2 | 2.63 | | 6.83E-05 | -0.35 | 9.26E-01 | | 1.63 | 1.17E-01 | |  | | REXO4 | 0.50 | 1.16E-03 | | 0.16 | | 6.60E-01 | 0.18 | 6.88E-01 | |
| DDIT4 | 2.49 | | 1.24E-51 | 0.17 | 6.77E-01 | | 0.15 | 8.00E-01 | |  | | RAD1 | 0.50 | 1.12E-03 | | 0.07 | | 8.91E-01 | -0.01 | 9.92E-01 | |
| LOC101927943 | 2.39 | | 9.10E-04 | 2.16 | 1.10E-02 | | 1.58 | 1.53E-01 | |  | | TADA2B | 0.50 | 9.69E-03 | | -0.04 | | 9.62E-01 | 0.18 | 6.99E-01 | |
| GGACT | 2.38 | | 6.01E-04 | 0.91 | 6.01E-01 | | 1.46 | 1.78E-01 | |  | | TNFSF15 | 0.50 | 3.77E-03 | | -0.29 | | 3.49E-01 | 0.06 | 9.24E-01 | |
| OTOP3 | 2.34 | | 7.95E-03 | -3.17 | 3.06E-01 | | -2.16 | 4.76E-01 | |  | | SMNDC1 | 0.50 | 7.96E-03 | | 0.26 | | 4.93E-01 | 0.28 | 5.29E-01 | |
| CTH | 2.32 | | 2.51E-52 | -0.36 | 3.23E-01 | | 0.15 | 7.76E-01 | |  | | C1orf226 | 0.50 | 2.45E-03 | | -0.06 | | 9.29E-01 | 0.38 | 2.20E-01 | |
| SMCO2 | 2.26 | | 9.74E-03 | 0.28 | 9.43E-01 | | 0.68 | 7.76E-01 | |  | | MSN | 0.50 | 2.15E-04 | | -0.08 | | 8.50E-01 | 0.24 | 4.50E-01 | |
| DDIT3 | 2.26 | | 3.64E-48 | 0.09 | 8.97E-01 | | -0.08 | 9.13E-01 | |  | | SLC38A1 | 0.50 | 3.79E-03 | | -0.02 | | 9.76E-01 | -0.09 | 8.92E-01 | |
| DDR2 | 2.24 | | 5.92E-03 | -0.65 | 8.51E-01 | | 0.76 | 7.12E-01 | |  | | ZNF408 | 0.50 | 1.77E-03 | | 0.09 | | 8.75E-01 | 0.23 | 6.05E-01 | |
| ASNS | 2.21 | | 3.08E-46 | -0.18 | 6.16E-01 | | -0.22 | 5.81E-01 | |  | | RFK | 0.50 | 4.86E-03 | | 0.31 | | 3.49E-01 | 0.47 | 1.82E-01 | |
| SGPP2 | 2.19 | | 7.74E-04 | -0.25 | 9.40E-01 | | 0.70 | 6.84E-01 | |  | | MYO1E | 0.49 | 4.80E-03 | | 0.30 | | 2.93E-01 | 0.29 | 3.90E-01 | |
| CFB | 2.15 | | 3.23E-04 | -0.36 | 8.96E-01 | | 0.95 | 4.38E-01 | |  | | DEPDC7 | 0.49 | 1.97E-03 | | -0.39 | | 9.17E-02 | -0.38 | 3.29E-01 | |
| TRIB3 | 2.13 | | 3.34E-32 | 0.04 | 9.54E-01 | | 0.11 | 8.49E-01 | |  | | RNF34 | 0.49 | 3.43E-04 | | 0.05 | | 9.29E-01 | 0.11 | 7.98E-01 | |
| GLI1 | 2.13 | | 1.40E-04 | -1.18 | 5.09E-01 | | -0.53 | 7.91E-01 | |  | | ST6GALNAC5 | 0.49 | 1.95E-03 | | -0.27 | | 3.30E-01 | -0.28 | 4.16E-01 | |
| SCN9A | 2.11 | | 1.18E-13 | -0.50 | 6.13E-01 | | 0.32 | 7.00E-01 | |  | | RSL1D1 | 0.49 | 1.79E-04 | | 0.26 | | 3.08E-01 | 0.14 | 7.58E-01 | |
| NMRAL2P | 2.11 | | 4.32E-31 | -0.12 | 8.52E-01 | | 0.46 | 2.36E-01 | |  | | SLC25A38 | 0.49 | 2.10E-03 | | -0.10 | | 8.43E-01 | 0.06 | 9.22E-01 | |
| CXCL2 | 2.05 | | 4.18E-03 | 0.66 | 7.42E-01 | | 1.18 | 3.32E-01 | |  | | NIPBL | 0.49 | 8.25E-04 | | -0.06 | | 9.15E-01 | -0.23 | 5.60E-01 | |
| GADD45A | 2.04 | | 2.85E-32 | -0.16 | 7.32E-01 | | 0.04 | 9.53E-01 | |  | | RAB11FIP5 | 0.49 | 7.07E-03 | | 0.30 | | 2.85E-01 | 0.46 | 1.73E-01 | |
| PLSCR4 | 2.02 | | 8.72E-06 | -1.38 | 2.31E-01 | | 0.45 | 7.02E-01 | |  | | STK39 | 0.49 | 4.85E-03 | | 0.20 | | 6.75E-01 | 0.50 | 1.43E-01 | |
| MSC | 1.94 | | 2.66E-06 | -0.83 | 4.85E-01 | | -0.84 | 4.83E-01 | |  | | TTC17 | 0.49 | 2.04E-03 | | 0.03 | | 9.56E-01 | 0.02 | 9.69E-01 | |
| SLC7A11 | 1.94 | | 2.74E-23 | -0.13 | 8.37E-01 | | -0.27 | 6.29E-01 | |  | | ZNHIT6 | 0.49 | 2.01E-03 | | 0.19 | | 6.65E-01 | 0.17 | 7.63E-01 | |
| ZNF391 | 1.92 | | 4.02E-08 | 0.31 | 7.71E-01 | | 0.03 | 9.84E-01 | |  | | ADNP | 0.49 | 6.24E-04 | | -0.19 | | 5.65E-01 | -0.14 | 7.57E-01 | |
| PCK2 | 1.90 | | 4.40E-34 | -0.02 | 9.70E-01 | | 0.11 | 8.48E-01 | |  | | ST6GALNAC2 | 0.49 | 1.78E-03 | | -0.49 | | 1.38E-02 | -0.08 | 8.81E-01 | |
| LINC00900 | 1.90 | | 1.50E-03 | -2.23 | 1.62E-01 | | -2.42 | 1.39E-01 | |  | | TNFAIP1 | 0.49 | 3.04E-03 | | 0.10 | | 8.19E-01 | 0.25 | 4.91E-01 | |
| PLAAT2 | 1.89 | | 1.66E-03 | 0.02 | 9.94E-01 | | 1.29 | 2.05E-01 | |  | | SLC50A1 | 0.49 | 1.21E-03 | | -0.06 | | 9.04E-01 | 0.19 | 6.56E-01 | |
| IL2RG | 1.88 | | 5.80E-03 | 0.21 | 9.43E-01 | | 0.78 | 6.05E-01 | |  | | SDAD1 | 0.49 | 4.86E-03 | | 0.22 | | 5.38E-01 | 0.28 | 4.68E-01 | |
| ARRDC4 | 1.88 | | 2.31E-26 | -0.49 | 1.82E-01 | | 0.18 | 7.52E-01 | |  | | EIF3J | 0.45 | 6.21E-03 | | 0.17 | | 6.98E-01 | -0.17 | 7.54E-01 | |
| PKN2-AS1 | 1.86 | | 2.82E-03 | 0.08 | 9.75E-01 | | 0.22 | 9.26E-01 | |  | | SLC35F2 | 0.45 | 6.63E-04 | | 0.12 | | 7.35E-01 | 0.12 | 7.66E-01 | |
| ZNF790 | 1.84 | | 4.83E-04 | 0.68 | 5.99E-01 | | 0.17 | 9.30E-01 | |  | | SREK1 | 0.45 | 1.06E-03 | | -0.08 | | 8.51E-01 | -0.27 | 4.66E-01 | |
| FAM107B | 1.81 | | 7.64E-30 | 0.43 | 1.63E-01 | | 0.22 | 6.14E-01 | |  | | ZKSCAN1 | 0.45 | 4.79E-03 | | 0.10 | | 8.22E-01 | 0.01 | 9.89E-01 | |
| KRT16 | 1.77 | | 3.17E-38 | -0.86 | 4.53E-06 | | 0.03 | 9.65E-01 | |  | | SQSTM1 | 0.45 | 8.60E-03 | | 0.03 | | 9.61E-01 | 0.53 | 1.26E-01 | |
| CREB5 | 1.76 | | 2.33E-03 | 0.11 | 9.65E-01 | | -0.94 | 6.05E-01 | |  | | SLC7A1 | 0.45 | 8.86E-03 | | -0.08 | | 8.80E-01 | 0.18 | 6.36E-01 | |
| ZNF750 | 1.74 | | 5.71E-05 | -0.05 | 9.78E-01 | | 0.99 | 1.44E-01 | |  | | THSD4 | 0.45 | 7.31E-03 | | 0.36 | | 1.44E-01 | 0.43 | 1.03E-01 | |
| ZNF311 | 1.73 | | 2.63E-03 | 0.58 | 6.77E-01 | | 0.49 | 7.26E-01 | |  | | RAD50 | 0.45 | 1.42E-03 | | 0.11 | | 7.89E-01 | 0.04 | 9.46E-01 | |
| MMP25 | 1.73 | | 4.60E-03 | -0.09 | 9.73E-01 | | 1.11 | 2.37E-01 | |  | | IGFBP7 | 0.45 | 2.40E-03 | | 0.04 | | 9.47E-01 | 0.11 | 8.16E-01 | |
| SNAI2 | 1.71 | | 1.55E-26 | -0.17 | 6.27E-01 | | -0.27 | 4.01E-01 | |  | | FAM177A1 | 0.45 | 8.73E-03 | | 0.03 | | 9.66E-01 | 0.19 | 6.89E-01 | |
| ZNF329 | 1.68 | | 3.86E-06 | 0.31 | 8.05E-01 | | 0.37 | 7.11E-01 | |  | | BAZ1A | 0.45 | 2.44E-03 | | 0.23 | | 4.22E-01 | 0.33 | 3.79E-01 | |
| HLA-DOB | 1.67 | | 5.29E-03 | 0.12 | 9.67E-01 | | 0.90 | 4.24E-01 | |  | | ATF6 | 0.45 | 9.01E-03 | | 0.03 | | 9.61E-01 | 0.02 | 9.75E-01 | |
| ERRFI1 | 1.67 | | 7.88E-20 | 0.02 | 9.81E-01 | | 0.04 | 9.52E-01 | |  | | ELMSAN1 | 0.45 | 1.97E-03 | | -0.09 | | 8.47E-01 | 0.13 | 7.57E-01 | |
| SLC6A9 | 1.65 | | 4.73E-32 | -0.40 | 9.03E-02 | | -0.06 | 9.20E-01 | |  | | NIFK | 0.44 | 3.92E-03 | | 0.09 | | 8.49E-01 | -0.10 | 8.54E-01 | |
| NRG1 | 1.65 | | 3.14E-34 | -0.05 | 9.26E-01 | | 0.17 | 6.36E-01 | |  | | MALT1 | 0.44 | 5.44E-03 | | 0.22 | | 5.70E-01 | 0.29 | 5.03E-01 | |
| KLF11 | 1.62 | | 3.02E-11 | 0.13 | 8.77E-01 | | 0.20 | 7.69E-01 | |  | | ZNF655 | 0.44 | 2.18E-03 | | -0.07 | | 9.00E-01 | -0.02 | 9.78E-01 | |
| ZNF501 | 1.62 | | 4.25E-04 | -0.46 | 7.78E-01 | | -0.45 | 7.44E-01 | |  | | IFIT5 | 0.44 | 6.18E-03 | | -0.22 | | 6.63E-01 | 0.28 | 5.10E-01 | |
| TSC22D1 | 1.62 | | 1.25E-35 | 0.39 | 1.40E-01 | | 0.40 | 1.80E-01 | |  | | NSD3 | 0.44 | 2.98E-03 | | -0.02 | | 9.70E-01 | -0.13 | 7.78E-01 | |
| ZBED3 | 1.62 | | 6.16E-09 | -0.22 | 8.29E-01 | | -0.02 | 9.86E-01 | |  | | PSEN1 | 0.44 | 4.57E-03 | | -0.09 | | 8.49E-01 | 0.02 | 9.67E-01 | |
| CEBPB-AS1 | 1.61 | | 8.28E-03 | 0.18 | 9.45E-01 | | -0.38 | 8.61E-01 | |  | | GNL3L | 0.44 | 5.94E-03 | | 0.32 | | 4.21E-01 | 0.41 | 1.19E-01 | |
| CFAP57 | 1.60 | | 2.10E-11 | -0.08 | 9.43E-01 | | 0.51 | 3.13E-01 | |  | | POLR3F | 0.44 | 8.79E-03 | | 0.20 | | 6.39E-01 | 0.20 | 6.98E-01 | |
| ZNF347 | 1.59 | | 8.60E-03 | 0.11 | 9.67E-01 | | -0.35 | 8.65E-01 | |  | | BRD2 | 0.44 | 6.45E-03 | | 0.18 | | 5.85E-01 | 0.37 | 1.48E-01 | |
| LACC1 | 1.59 | | 1.85E-14 | 0.42 | 3.20E-01 | | 0.59 | 2.37E-01 | |  | | CASP7 | 0.43 | 7.37E-03 | | -0.64 | | 1.22E-03 | 0.11 | 8.22E-01 | |
| HIST2H4A | 1.58 | | 7.77E-06 | 0.23 | 8.51E-01 | | 0.41 | 6.51E-01 | |  | | TRIM44 | 0.43 | 1.12E-03 | | -0.03 | | 9.46E-01 | 0.14 | 7.24E-01 | |
| LINC01589 | 1.57 | | 2.67E-03 | 0.57 | 6.70E-01 | | 0.56 | 6.33E-01 | |  | | HPS5 | 0.43 | 9.03E-03 | | -0.08 | | 9.19E-01 | 0.14 | 7.80E-01 | |
| BTG1 | 1.57 | | 3.48E-28 | 0.08 | 8.97E-01 | | 0.44 | 1.25E-01 | |  | | KCMF1 | 0.43 | 3.04E-03 | | -0.21 | | 5.53E-01 | -0.35 | 1.84E-01 | |
| ZNF805 | 1.56 | | 1.89E-10 | 0.03 | 9.79E-01 | | 0.23 | 7.69E-01 | |  | | ELP3 | 0.43 | 3.36E-03 | | -0.05 | | 9.39E-01 | 0.00 | 9.97E-01 | |
| TUBE1 | 1.53 | | 3.17E-20 | 0.15 | 7.59E-01 | | -0.07 | 9.20E-01 | |  | | ZBTB38 | 0.43 | 9.65E-03 | | 0.10 | | 8.23E-01 | 0.01 | 9.83E-01 | |
| TRAF1 | 1.53 | | 2.35E-08 | -0.18 | 8.71E-01 | | 0.65 | 1.72E-01 | |  | | SRSF7 | 0.43 | 9.90E-04 | | 0.04 | | 9.44E-01 | 0.01 | 9.78E-01 | |
| NDUFA4L2 | 1.50 | | 9.03E-04 | -0.53 | 7.25E-01 | | 0.06 | 9.71E-01 | |  | | EAPP | 0.43 | 3.88E-03 | | 0.01 | | 9.88E-01 | -0.04 | 9.36E-01 | |
| MXD1 | 1.50 | | 7.85E-11 | 0.24 | 6.07E-01 | | 0.44 | 2.48E-01 | |  | | PISD | 0.43 | 8.98E-03 | | -0.07 | | 8.99E-01 | 0.16 | 7.47E-01 | |
| RND3 | 1.49 | | 1.73E-20 | -0.36 | 1.55E-01 | | -0.31 | 3.39E-01 | |  | | NR1H2 | 0.43 | 5.25E-03 | | 0.25 | | 5.09E-01 | 0.51 | 1.51E-01 | |
| FAM241A | 1.48 | | 2.43E-11 | 0.52 | 1.37E-01 | | 0.43 | 3.74E-01 | |  | | ATG13 | 0.43 | 3.64E-03 | | 0.13 | | 7.43E-01 | 0.29 | 3.62E-01 | |
| FSD1L | 1.48 | | 3.44E-04 | 0.23 | 8.45E-01 | | 0.33 | 7.59E-01 | |  | | RNF115 | 0.42 | 9.04E-03 | | -0.31 | | 2.34E-01 | -0.23 | 5.36E-01 | |
| TTC39B | 1.47 | | 1.21E-10 | 0.20 | 7.80E-01 | | 0.39 | 4.84E-01 | |  | | STIL | 0.42 | 7.94E-03 | | -0.13 | | 7.69E-01 | 0.00 | 9.96E-01 | |
| BIRC3 | 1.44 | | 7.82E-10 | -0.43 | 2.54E-01 | | 0.19 | 8.11E-01 | |  | | CEP57 | 0.42 | 8.81E-03 | | -0.07 | | 8.99E-01 | -0.19 | 6.88E-01 | |
| PSAT1 | 1.43 | | 1.22E-29 | -0.25 | 3.91E-01 | | -0.33 | 2.74E-01 | |  | | WDR74 | 0.41 | 1.51E-03 | | 0.22 | | 5.08E-01 | 0.35 | 3.82E-01 | |
| TXNRD1 | 1.43 | | 1.93E-16 | -0.67 | 3.27E-03 | | -0.22 | 5.96E-01 | |  | | PRRC2C | 0.41 | 7.54E-03 | | 0.16 | | 6.53E-01 | 0.19 | 6.21E-01 | |
| ZNF628 | 1.41 | | 8.19E-03 | 0.73 | 5.29E-01 | | 0.23 | 8.88E-01 | |  | | KANSL2 | 0.41 | 3.53E-03 | | -0.08 | | 8.66E-01 | -0.07 | 8.85E-01 | |
| ZBTB10 | 1.40 | | 4.78E-03 | -0.13 | 9.49E-01 | | -1.08 | 3.60E-01 | |  | | USP15 | 0.40 | 3.54E-03 | | -0.16 | | 7.74E-01 | -0.03 | 9.66E-01 | |
| SNAPC1 | 1.40 | | 9.94E-13 | 0.37 | 2.86E-01 | | 0.27 | 6.30E-01 | |  | | ARHGAP12 | 0.40 | 9.88E-03 | | -0.05 | | 9.46E-01 | -0.07 | 9.18E-01 | |
| ZNF239 | 1.40 | | 7.77E-03 | 0.73 | 4.47E-01 | | 0.29 | 8.28E-01 | |  | | LSG1 | 0.40 | 7.34E-03 | | -0.02 | | 9.69E-01 | 0.04 | 9.36E-01 | |
| TXNL4B | 1.39 | | 1.35E-19 | 0.05 | 9.41E-01 | | 0.16 | 7.44E-01 | |  | | COQ5 | 0.40 | 5.40E-03 | | -0.26 | | 3.59E-01 | -0.30 | 3.04E-01 | |
| ZNF441 | 1.38 | | 5.21E-06 | -0.02 | 9.87E-01 | | -0.17 | 8.66E-01 | |  | | PSMA4 | 0.40 | 3.35E-03 | | -0.14 | | 6.46E-01 | 0.05 | 9.32E-01 | |
| SLC1A4 | 1.38 | | 3.47E-21 | -0.18 | 6.21E-01 | | -0.04 | 9.37E-01 | |  | | TRIP6 | 0.40 | 3.79E-03 | | 0.11 | | 8.02E-01 | 0.23 | 5.78E-01 | |
| HEIH | 1.38 | | 1.33E-12 | 0.03 | 9.71E-01 | | 0.15 | 8.10E-01 | |  | | CPSF4 | 0.40 | 9.45E-03 | | 0.13 | | 7.70E-01 | 0.13 | 7.94E-01 | |
| AVPI1 | 1.36 | | 5.27E-19 | 0.12 | 8.03E-01 | | 0.05 | 9.37E-01 | |  | | ARFRP1 | 0.40 | 5.17E-03 | | 0.17 | | 6.96E-01 | 0.20 | 6.70E-01 | |
| TGIF1 | 1.36 | | 4.76E-21 | 0.11 | 8.16E-01 | | 0.20 | 6.21E-01 | |  | | SMAD3 | 0.40 | 4.37E-03 | | 0.19 | | 5.47E-01 | 0.42 | 1.01E-01 | |
| F3 | 1.35 | | 5.27E-30 | -0.08 | 8.65E-01 | | 0.15 | 7.66E-01 | |  | | TRIM27 | 0.40 | 3.66E-03 | | 0.23 | | 3.55E-01 | 0.40 | 1.25E-01 | |
| GPT2 | 1.34 | | 7.81E-20 | -0.04 | 9.40E-01 | | 0.00 | 9.97E-01 | |  | | CIRBP | 0.40 | 9.32E-03 | | 0.02 | | 9.76E-01 | 0.15 | 7.25E-01 | |
| ZNF274 | 1.34 | | 2.74E-13 | 0.07 | 9.26E-01 | | 0.45 | 1.41E-01 | |  | | GCLC | 0.39 | 9.90E-03 | | -0.58 | | 2.41E-02 | -0.37 | 2.70E-01 | |
| MAP1B | 1.34 | | 2.00E-10 | -0.03 | 9.68E-01 | | -0.19 | 7.57E-01 | |  | | MAK16 | 0.39 | 8.92E-03 | | 0.07 | | 9.08E-01 | -0.07 | 9.28E-01 | |
| CEACAM5 | 1.34 | | 1.48E-12 | -1.39 | 2.58E-07 | | -0.16 | 8.02E-01 | |  | | PLEKHM2 | 0.39 | 7.48E-03 | | 0.13 | | 7.72E-01 | 0.27 | 5.18E-01 | |
| MLLT3 | 1.34 | | 3.64E-05 | -0.12 | 9.28E-01 | | 0.27 | 7.41E-01 | |  | | TIMM44 | 0.39 | 4.08E-03 | | -0.05 | | 9.34E-01 | -0.06 | 9.31E-01 | |
| LINC01881 | 1.33 | | 4.09E-03 | 0.63 | 5.19E-01 | | 0.34 | 7.69E-01 | |  | | GABPB1 | 0.39 | 5.29E-03 | | -0.16 | | 6.70E-01 | -0.19 | 6.63E-01 | |
| MTHFD2 | 1.32 | | 2.40E-25 | -0.03 | 9.61E-01 | | 0.10 | 8.59E-01 | |  | | BTF3L4 | 0.39 | 7.45E-03 | | -0.13 | | 7.60E-01 | -0.19 | 6.94E-01 | |
| ARNTL | 1.32 | | 1.08E-15 | 0.59 | 1.54E-02 | | 0.50 | 1.47E-01 | |  | | TMBIM1 | 0.39 | 4.82E-03 | | 0.13 | | 7.08E-01 | 0.24 | 4.42E-01 | |
| LINC00184 | 1.31 | | 4.21E-03 | 1.01 | 8.14E-02 | | 0.95 | 1.28E-01 | |  | | NOP56 | 0.39 | 4.63E-03 | | 0.25 | | 3.63E-01 | 0.31 | 3.61E-01 | |
| CYLD | 1.31 | | 2.57E-18 | -0.37 | 1.74E-01 | | 0.49 | 1.21E-01 | |  | | AGAP3 | 0.39 | 5.06E-03 | | -0.05 | | 9.29E-01 | 0.02 | 9.77E-01 | |
| SLC38A2 | 1.30 | | 2.42E-13 | -0.11 | 8.72E-01 | | 0.00 | 9.97E-01 | |  | | TRIM11 | 0.39 | 8.74E-03 | | 0.17 | | 6.24E-01 | 0.25 | 4.91E-01 | |
| CYP24A1 | 1.30 | | 1.52E-09 | -0.48 | 3.78E-01 | | -0.31 | 6.24E-01 | |  | | RAB18 | 0.39 | 4.91E-03 | | 0.07 | | 9.02E-01 | 0.03 | 9.65E-01 | |
| SNHG1 | 1.30 | | 4.69E-15 | 0.19 | 6.51E-01 | | 0.09 | 8.72E-01 | |  | | FAM120AOS | 0.39 | 7.48E-03 | | -0.19 | | 6.02E-01 | -0.03 | 9.53E-01 | |
| CEBPG | 1.29 | | 1.47E-19 | -0.09 | 8.52E-01 | | -0.14 | 7.32E-01 | |  | | MARCH7_ | 0.38 | 9.54E-03 | | -0.25 | | 5.09E-01 | -0.30 | 4.90E-01 | |
| ARL4A | 1.29 | | 2.02E-14 | -0.11 | 8.35E-01 | | -0.08 | 8.95E-01 | |  | | DHRS7 | 0.38 | 9.46E-03 | | 0.08 | | 8.87E-01 | 0.39 | 1.20E-01 | |
| ZNF416 | 1.29 | | 8.26E-06 | 0.37 | 5.27E-01 | | 0.12 | 8.90E-01 | |  | | MTIF2 | 0.38 | 6.74E-03 | | 0.08 | | 8.52E-01 | -0.04 | 9.38E-01 | |
| CCDC122 | 1.27 | | 4.44E-06 | 0.35 | 6.01E-01 | | 0.05 | 9.67E-01 | |  | | TARS | 0.38 | 7.38E-03 | | -0.15 | | 7.17E-01 | -0.19 | 6.44E-01 | |
| NATD1 | 1.27 | | 2.03E-03 | 0.70 | 3.14E-01 | | 0.91 | 1.28E-01 | |  | | NEDD1 | 0.38 | 7.84E-03 | | -0.16 | | 7.52E-01 | 0.00 | 9.98E-01 | |
| IL11 | 1.27 | | 4.53E-05 | 0.48 | 3.96E-01 | | 0.67 | 1.77E-01 | |  | | GTF2H1 | 0.37 | 6.82E-03 | | -0.15 | | 7.73E-01 | -0.13 | 7.91E-01 | |
| CNOT6LP1 | 1.27 | | 9.47E-03 | -0.19 | 9.26E-01 | | -0.79 | 4.60E-01 | |  | | ERP29 | 0.37 | 4.84E-03 | | -0.04 | | 9.44E-01 | 0.05 | 9.28E-01 | |
| GATA6 | 1.27 | | 1.54E-12 | 0.33 | 4.24E-01 | | 0.23 | 6.70E-01 | |  | | ANXA7 | 0.37 | 4.40E-03 | | -0.09 | | 8.63E-01 | 0.16 | 6.70E-01 | |
| OSGIN1 | 1.26 | | 2.25E-12 | -0.27 | 5.12E-01 | | -0.07 | 9.28E-01 | |  | | H3F3B | 0.37 | 9.54E-03 | | 0.21 | | 4.31E-01 | 0.30 | 2.88E-01 | |
| ZNF623 | 1.26 | | 7.58E-11 | -0.25 | 6.33E-01 | | -0.16 | 7.82E-01 | |  | | ARF6 | 0.36 | 4.61E-03 | | -0.19 | | 5.82E-01 | 0.03 | 9.60E-01 | |
| PTP4A1 | 1.26 | | 2.09E-19 | 0.28 | 3.34E-01 | | 0.31 | 4.85E-01 | |  | | HGH1 | 0.36 | 9.75E-03 | | 0.19 | | 6.15E-01 | 0.21 | 6.43E-01 | |
| SEPSECS | 1.26 | | 2.01E-09 | 0.13 | 8.68E-01 | | 0.22 | 7.49E-01 | |  | | SCO1 | 0.36 | 7.74E-03 | | -0.14 | | 7.24E-01 | 0.08 | 8.56E-01 | |
| ORAI3 | 1.26 | | 1.99E-05 | 0.61 | 2.17E-01 | | 0.56 | 3.30E-01 | |  | | AP2M1 | -0.34 | 9.99E-03 | | -0.28 | | 2.34E-01 | -0.37 | 2.14E-01 | |
| XBP1 | 1.25 | | 2.78E-24 | -0.11 | 7.59E-01 | | -0.18 | 6.09E-01 | |  | | TIMM17A | -0.35 | 9.09E-03 | | -0.05 | | 9.29E-01 | -0.13 | 7.34E-01 | |
| SIX5 | 1.25 | | 7.90E-03 | 0.73 | 4.40E-01 | | 0.86 | 2.51E-01 | |  | | TSEN34 | -0.36 | 6.29E-03 | | 0.14 | | 7.27E-01 | 0.21 | 6.46E-01 | |
| CAPNS2 | 1.25 | | 2.32E-07 | 0.21 | 7.22E-01 | | 0.45 | 2.58E-01 | |  | | MAVS | -0.37 | 8.77E-03 | | -0.05 | | 9.24E-01 | -0.05 | 9.32E-01 | |
| DYRK1B | 1.25 | | 7.86E-05 | -0.09 | 9.46E-01 | | 0.39 | 6.21E-01 | |  | | EMP1 | -0.37 | 7.07E-03 | | 0.23 | | 4.21E-01 | -0.06 | 9.02E-01 | |
| AJUBA | 1.25 | | 2.70E-24 | -0.28 | 1.73E-01 | | -0.17 | 6.25E-01 | |  | | PRC1 | -0.37 | 5.86E-03 | | -0.20 | | 4.47E-01 | -0.27 | 3.50E-01 | |
| OSBPL6 | 1.24 | | 3.91E-03 | 0.68 | 3.48E-01 | | 0.19 | 8.71E-01 | |  | | NUP85 | -0.37 | 9.22E-03 | | -0.18 | | 5.51E-01 | -0.17 | 6.83E-01 | |
| CREBRF | 1.24 | | 4.58E-05 | -0.56 | 3.95E-01 | | 0.20 | 8.14E-01 | |  | | CHID1 | -0.38 | 5.66E-03 | | -0.04 | | 9.44E-01 | 0.02 | 9.74E-01 | |
| LOC102724889 | 1.24 | | 2.89E-03 | 0.83 | 1.62E-01 | | 0.82 | 2.61E-01 | |  | | BIRC5 | -0.38 | 5.03E-03 | | -0.17 | | 5.61E-01 | -0.24 | 4.66E-01 | |
| DLG1-AS1 | 1.23 | | 7.59E-06 | 0.01 | 9.90E-01 | | -0.46 | 4.88E-01 | |  | | HNRNPH3 | -0.38 | 5.41E-03 | | -0.07 | | 8.71E-01 | -0.18 | 6.32E-01 | |
| ZNF140 | 1.23 | | 9.05E-12 | -0.11 | 8.80E-01 | | 0.10 | 8.94E-01 | |  | | LDHA | -0.38 | 7.54E-03 | | -0.15 | | 7.19E-01 | -0.14 | 7.57E-01 | |
| SPECC1L | 1.22 | | 5.45E-08 | 0.27 | 6.32E-01 | | 0.31 | 5.64E-01 | |  | | SCAMP2 | -0.38 | 6.57E-03 | | 0.02 | | 9.71E-01 | 0.10 | 8.51E-01 | |
| PIK3IP1 | 1.22 | | 1.15E-04 | -1.18 | 3.44E-02 | | -0.48 | 5.29E-01 | |  | | SNRNP40 | -0.38 | 6.72E-03 | | -0.11 | | 7.57E-01 | -0.09 | 8.26E-01 | |
| TLCD5 | 1.22 | | 1.92E-04 | 0.74 | 1.28E-01 | | 0.39 | 5.74E-01 | |  | | TK1 | -0.38 | 3.46E-03 | | -0.23 | | 5.38E-01 | -0.12 | 8.53E-01 | |
| BCL6 | 1.22 | | 2.52E-07 | -0.57 | 2.37E-01 | | -0.10 | 9.05E-01 | |  | | PDIA6 | -0.38 | 3.79E-03 | | 0.03 | | 9.54E-01 | 0.14 | 7.29E-01 | |
| RGS3 | 1.21 | | 1.47E-18 | -0.13 | 7.69E-01 | | 0.23 | 5.72E-01 | |  | | PSMC5 | -0.38 | 7.46E-03 | | -0.12 | | 7.44E-01 | -0.11 | 8.19E-01 | |
| ALPK1 | 1.21 | | 2.94E-08 | -0.30 | 6.63E-01 | | 0.55 | 1.35E-01 | |  | | CD63 | -0.39 | 4.37E-03 | | 0.21 | | 4.38E-01 | 0.10 | 8.29E-01 | |
| KDM7A-DT | 1.19 | | 8.99E-03 | 0.75 | 3.29E-01 | | 0.76 | 2.67E-01 | |  | | DCAF13 | -0.39 | 5.70E-03 | | -0.08 | | 8.59E-01 | -0.24 | 6.07E-01 | |
| SLC22A15 | 1.19 | | 2.89E-06 | -0.17 | 8.20E-01 | | -0.66 | 1.28E-01 | |  | | PCMT1 | -0.39 | 6.35E-03 | | -0.06 | | 8.91E-01 | -0.34 | 2.28E-01 | |
| RNF217 | 1.19 | | 2.75E-10 | -0.28 | 6.01E-01 | | -0.06 | 9.43E-01 | |  | | CLPTM1L | -0.39 | 8.72E-03 | | 0.13 | | 7.17E-01 | 0.27 | 4.69E-01 | |
| C3 | 1.19 | | 1.34E-17 | -0.44 | 4.12E-02 | | 0.18 | 6.50E-01 | |  | | OAZ2 | -0.39 | 7.71E-03 | | 0.15 | | 6.53E-01 | -0.04 | 9.32E-01 | |
| RALA | 1.19 | | 2.31E-21 | 0.19 | 4.88E-01 | | 0.36 | 2.12E-01 | |  | | PIGS | -0.39 | 7.48E-03 | | -0.33 | | 1.34E-01 | -0.39 | 1.54E-01 | |
| ZNF19 | 1.18 | | 1.61E-04 | 0.12 | 9.28E-01 | | -0.09 | 9.32E-01 | |  | | MAN2A1 | -0.40 | 9.99E-03 | | 0.05 | | 9.40E-01 | -0.16 | 7.66E-01 | |
| ZFAND1 | 1.18 | | 2.42E-13 | 0.28 | 3.82E-01 | | 0.20 | 7.42E-01 | |  | | FAM83D | -0.40 | 2.56E-03 | | -0.36 | | 8.49E-02 | -0.14 | 7.12E-01 | |
| CARS | 1.17 | | 3.76E-17 | -0.07 | 8.85E-01 | | 0.01 | 9.82E-01 | |  | | EXOSC6 | -0.40 | 4.82E-03 | | 0.12 | | 7.85E-01 | 0.20 | 6.47E-01 | |
| ZNF319 | 1.16 | | 9.19E-08 | 0.33 | 4.66E-01 | | 0.57 | 1.50E-01 | |  | | EPS8L2 | -0.40 | 2.46E-03 | | -0.48 | | 1.19E-02 | -0.45 | 1.87E-01 | |
| ZNF133 | 1.16 | | 2.39E-14 | 0.01 | 9.82E-01 | | 0.26 | 5.09E-01 | |  | | SLBP | -0.40 | 6.05E-03 | | -0.07 | | 8.70E-01 | -0.06 | 8.90E-01 | |
| PRKAB2 | 1.16 | | 2.46E-10 | -0.25 | 5.11E-01 | | -0.33 | 4.14E-01 | |  | | ATG4B | -0.40 | 8.20E-03 | | -0.01 | | 9.80E-01 | -0.01 | 9.88E-01 | |
| GTF2IP13 | 1.16 | | 1.36E-04 | -0.07 | 9.59E-01 | | 0.69 | 1.28E-01 | |  | | NAXE | -0.40 | 4.66E-03 | | -0.10 | | 7.96E-01 | -0.28 | 4.45E-01 | |
| IL20RB | 1.15 | | 3.09E-17 | -0.32 | 2.06E-01 | | -0.19 | 6.08E-01 | |  | | HPS6 | -0.40 | 7.03E-03 | | 0.06 | | 9.17E-01 | 0.27 | 4.98E-01 | |
| PEAR1 | 1.15 | | 1.30E-04 | 0.33 | 6.69E-01 | | 0.34 | 6.21E-01 | |  | | CYB561A3 | -0.40 | 7.54E-03 | | -0.10 | | 8.20E-01 | 0.08 | 8.90E-01 | |
| UHRF1BP1 | 1.15 | | 4.77E-09 | -0.53 | 3.48E-02 | | -0.48 | 1.25E-01 | |  | | NUP37 | -0.40 | 5.66E-03 | | -0.01 | | 9.83E-01 | -0.18 | 6.56E-01 | |
| YPEL5 | 1.14 | | 1.81E-11 | -0.23 | 6.07E-01 | | 0.18 | 7.32E-01 | |  | | PRMT2 | -0.41 | 6.40E-03 | | -0.15 | | 6.63E-01 | -0.09 | 8.63E-01 | |
| CEP120 | 1.14 | | 1.47E-08 | 0.14 | 8.12E-01 | | 0.08 | 9.12E-01 | |  | | CDK1 | -0.41 | 5.28E-03 | | -0.10 | | 8.24E-01 | -0.25 | 6.14E-01 | |
| DNAJB9 | 1.14 | | 1.72E-11 | 0.12 | 8.19E-01 | | 0.21 | 7.35E-01 | |  | | PEX6 | -0.41 | 7.48E-03 | | 0.08 | | 8.69E-01 | 0.17 | 7.35E-01 | |
| SLC1A5 | 1.13 | | 1.81E-19 | 0.09 | 8.10E-01 | | 0.31 | 3.39E-01 | |  | | SCARB1 | -0.41 | 7.35E-03 | | -0.04 | | 9.54E-01 | -0.04 | 9.50E-01 | |
| C16orf72 | 1.13 | | 1.79E-11 | 0.15 | 7.74E-01 | | 0.16 | 7.78E-01 | |  | | SAMM50 | -0.41 | 3.86E-03 | | -0.23 | | 3.93E-01 | -0.19 | 6.09E-01 | |
| KLF6 | 1.13 | | 1.77E-18 | 0.01 | 9.79E-01 | | 0.29 | 3.37E-01 | |  | | ATP6V1A | -0.41 | 4.18E-03 | | -0.14 | | 7.22E-01 | -0.07 | 9.12E-01 | |
| GARS | 1.13 | | 1.35E-15 | -0.11 | 7.80E-01 | | -0.09 | 8.52E-01 | |  | | DBNL | -0.41 | 2.42E-03 | | 0.06 | | 9.13E-01 | 0.14 | 7.91E-01 | |
| ZNF543 | 1.13 | | 4.56E-07 | 0.00 | 9.98E-01 | | -0.18 | 7.88E-01 | |  | | PLPP2 | -0.41 | 3.77E-03 | | 0.12 | | 7.67E-01 | -0.08 | 8.92E-01 | |
| RUNX1 | 1.12 | | 8.23E-14 | 0.08 | 8.80E-01 | | 0.18 | 6.52E-01 | |  | | HDAC1 | -0.41 | 4.25E-03 | | -0.12 | | 7.72E-01 | -0.10 | 8.32E-01 | |
| CPEB2 | 1.12 | | 2.10E-05 | -0.58 | 3.01E-01 | | -0.18 | 8.25E-01 | |  | | MORF4L2 | -0.41 | 5.14E-03 | | -0.25 | | 4.25E-01 | -0.35 | 2.68E-01 | |
| MTMR3 | 1.12 | | 1.74E-03 | -0.01 | 9.96E-01 | | -0.03 | 9.78E-01 | |  | | NIPA2 | -0.42 | 6.36E-03 | | -0.06 | | 9.15E-01 | -0.05 | 9.24E-01 | |
| MIA2 | 1.12 | | 2.80E-03 | 0.20 | 8.56E-01 | | 0.14 | 8.98E-01 | |  | | VCP | -0.42 | 7.09E-03 | | -0.03 | | 9.51E-01 | 0.17 | 7.02E-01 | |
| AARS | 1.11 | | 5.33E-14 | -0.24 | 3.88E-01 | | -0.22 | 5.54E-01 | |  | | TTC3 | -0.42 | 8.07E-03 | | -0.14 | | 7.19E-01 | -0.46 | 1.29E-01 | |
| ZNF768 | 1.11 | | 7.91E-09 | 0.30 | 5.16E-01 | | 0.42 | 2.95E-01 | |  | | CD276 | -0.42 | 1.41E-03 | | 0.06 | | 8.85E-01 | 0.11 | 8.28E-01 | |
| THAP9-AS1 | 1.11 | | 8.59E-10 | -0.05 | 9.54E-01 | | -0.17 | 7.75E-01 | |  | | FAM98A | -0.42 | 8.73E-03 | | -0.07 | | 8.99E-01 | -0.03 | 9.62E-01 | |
| MDM4 | 1.11 | | 4.10E-10 | -0.11 | 8.63E-01 | | 0.05 | 9.44E-01 | |  | | ATP6V1B2 | -0.42 | 8.67E-03 | | -0.04 | | 9.48E-01 | -0.01 | 9.88E-01 | |
| C1RL | 1.11 | | 1.85E-04 | 0.22 | 8.02E-01 | | 0.72 | 1.02E-01 | |  | | PRPS2 | -0.42 | 1.26E-03 | | -0.02 | | 9.76E-01 | -0.09 | 8.54E-01 | |
| STK17B | 1.11 | | 5.67E-09 | 0.21 | 6.83E-01 | | 0.29 | 6.05E-01 | |  | | SPG7 | -0.42 | 4.37E-03 | | -0.01 | | 9.81E-01 | 0.06 | 9.30E-01 | |
| IRS1 | 1.10 | | 1.01E-05 | 0.45 | 2.97E-01 | | 0.60 | 1.17E-01 | |  | | EI24 | -0.42 | 2.43E-03 | | -0.05 | | 9.22E-01 | -0.16 | 6.58E-01 | |
| CLUAP1 | 1.10 | | 2.94E-14 | -0.29 | 3.93E-01 | | 0.34 | 3.00E-01 | |  | | SARAF | -0.42 | 1.44E-03 | | -0.03 | | 9.70E-01 | -0.13 | 7.90E-01 | |
| SLC30A1 | 1.10 | | 5.43E-10 | -0.27 | 5.39E-01 | | -0.14 | 8.36E-01 | |  | | SDC1 | -0.42 | 5.77E-03 | | 0.35 | | 1.21E-01 | 0.42 | 1.51E-01 | |
| ZXDB | 1.10 | | 1.19E-07 | -0.01 | 9.92E-01 | | 0.03 | 9.67E-01 | |  | | CINP | -0.42 | 7.40E-03 | | 0.11 | | 7.94E-01 | 0.11 | 8.23E-01 | |
| ZNF711 | 1.09 | | 3.81E-03 | -0.64 | 4.75E-01 | | -0.60 | 4.83E-01 | |  | | RBM14 | -0.42 | 2.70E-03 | | -0.02 | | 9.67E-01 | 0.12 | 7.81E-01 | |
| NFE2L2 | 1.09 | | 3.04E-15 | -0.29 | 2.40E-01 | | -0.23 | 5.22E-01 | |  | | ARHGAP27 | -0.42 | 5.66E-03 | | 0.11 | | 8.14E-01 | 0.07 | 9.12E-01 | |
| RNF43 | 1.09 | | 1.66E-06 | -0.04 | 9.59E-01 | | -0.13 | 8.28E-01 | |  | | COPS7B | -0.42 | 7.12E-03 | | -0.09 | | 8.31E-01 | -0.11 | 8.11E-01 | |
| R3HCC1L | 1.09 | | 2.21E-10 | -0.01 | 9.85E-01 | | 0.03 | 9.67E-01 | |  | | FANCG | -0.43 | 5.98E-03 | | -0.01 | | 9.85E-01 | 0.01 | 9.84E-01 | |
| ETS2 | 1.08 | | 9.55E-11 | -0.17 | 7.08E-01 | | 0.12 | 8.26E-01 | |  | | FBXO21 | -0.43 | 2.80E-03 | | -0.05 | | 9.27E-01 | -0.11 | 8.08E-01 | |
| CBX4 | 1.08 | | 4.17E-07 | 0.25 | 6.18E-01 | | 0.44 | 2.54E-01 | |  | | PMPCB | -0.43 | 4.65E-03 | | -0.14 | | 6.99E-01 | -0.31 | 3.44E-01 | |
| BACH1 | 1.08 | | 9.27E-09 | -0.25 | 5.87E-01 | | 0.38 | 3.56E-01 | |  | | EIF3E | -0.43 | 3.08E-03 | | 0.05 | | 9.19E-01 | -0.26 | 5.47E-01 | |
| ZCCHC8 | 1.08 | | 9.60E-13 | -0.20 | 6.36E-01 | | 0.15 | 7.52E-01 | |  | | ATG9A | -0.43 | 5.91E-03 | | -0.25 | | 4.22E-01 | -0.37 | 2.79E-01 | |
| ABL2 | 1.08 | | 4.13E-11 | 0.27 | 2.99E-01 | | 0.23 | 5.58E-01 | |  | | YWHAH | -0.43 | 1.89E-03 | | -0.16 | | 6.36E-01 | -0.09 | 8.31E-01 | |
| H1F0 | 1.08 | | 2.79E-18 | 0.01 | 9.76E-01 | | 0.18 | 5.80E-01 | |  | | ABCF3 | -0.43 | 2.32E-03 | | -0.09 | | 8.59E-01 | -0.09 | 8.69E-01 | |
| ETV6 | 1.08 | | 1.89E-10 | 0.04 | 9.56E-01 | | 0.30 | 4.45E-01 | |  | | SCAP | -0.43 | 5.40E-03 | | -0.03 | | 9.67E-01 | 0.01 | 9.89E-01 | |
| GRPEL2 | 1.08 | | 5.37E-10 | -0.26 | 5.60E-01 | | -0.44 | 3.09E-01 | |  | | TTF2 | -0.43 | 2.98E-03 | | -0.17 | | 6.14E-01 | -0.16 | 6.85E-01 | |
| GTPBP2 | 1.08 | | 3.69E-06 | -0.29 | 4.95E-01 | | 0.09 | 8.96E-01 | |  | | CHAF1B | -0.43 | 5.58E-03 | | -0.24 | | 4.95E-01 | -0.26 | 4.54E-01 | |
| ZNF672 | 1.08 | | 1.44E-12 | -0.20 | 5.99E-01 | | 0.44 | 1.93E-01 | |  | | NSDHL | -0.43 | 3.47E-03 | | -0.23 | | 4.27E-01 | -0.22 | 5.94E-01 | |
| NFIL3 | 1.08 | | 5.00E-06 | -0.47 | 2.76E-01 | | -0.34 | 4.51E-01 | |  | | HADH | -0.43 | 4.39E-03 | | -0.23 | | 4.39E-01 | -0.29 | 3.11E-01 | |
| KLF7 | 1.07 | | 2.47E-08 | -0.05 | 9.47E-01 | | 0.12 | 8.36E-01 | |  | | EBP | -0.43 | 3.75E-03 | | -0.24 | | 4.02E-01 | -0.22 | 5.85E-01 | |
| DCLRE1C | 1.07 | | 1.16E-14 | -0.01 | 9.79E-01 | | 0.15 | 7.74E-01 | |  | | VPS26B | -0.44 | 2.59E-03 | | -0.08 | | 8.65E-01 | -0.32 | 3.39E-01 | |
| GADD45B | 1.07 | | 1.17E-08 | -0.43 | 1.61E-01 | | -0.23 | 6.29E-01 | |  | | DLGAP5 | -0.44 | 9.78E-04 | | -0.28 | | 4.25E-01 | -0.35 | 3.63E-01 | |
| ZNF596 | 1.07 | | 1.36E-03 | 0.13 | 9.21E-01 | | 0.09 | 9.36E-01 | |  | | ATP6V1C1 | -0.44 | 2.17E-03 | | 0.07 | | 8.85E-01 | -0.08 | 8.98E-01 | |
| TUFT1 | 1.06 | | 1.95E-10 | -0.32 | 2.49E-01 | | -0.18 | 6.64E-01 | |  | | RRM1 | -0.44 | 4.58E-03 | | -0.38 | | 1.74E-01 | -0.37 | 1.78E-01 | |
| ANKRD33B | 1.06 | | 1.34E-06 | -0.36 | 4.13E-01 | | -0.08 | 9.05E-01 | |  | | TRIAP1 | -0.44 | 3.69E-03 | | -0.06 | | 9.14E-01 | -0.34 | 2.76E-01 | |
| KMO | 1.06 | | 4.25E-09 | -1.57 | 4.34E-09 | | -0.55 | 1.17E-01 | |  | | LRPAP1 | -0.44 | 4.19E-03 | | -0.04 | | 9.45E-01 | 0.16 | 7.92E-01 | |
| KLF3 | 1.05 | | 3.21E-10 | -0.24 | 5.19E-01 | | -0.04 | 9.60E-01 | |  | | NUSAP1 | -0.44 | 7.76E-04 | | -0.34 | | 9.84E-02 | -0.40 | 1.22E-01 | |
| ZNF627 | 1.05 | | 2.04E-06 | -0.30 | 6.08E-01 | | -0.10 | 8.91E-01 | |  | | THYN1 | -0.44 | 8.28E-03 | | 0.14 | | 7.14E-01 | -0.12 | 7.90E-01 | |
| TP63 | 1.05 | | 6.81E-11 | 0.04 | 9.58E-01 | | 0.26 | 5.37E-01 | |  | | CFL2 | -0.44 | 8.87E-03 | | -0.37 | | 3.19E-01 | -0.35 | 4.81E-01 | |
| GAS5 | 1.04 | | 3.06E-15 | 0.24 | 3.71E-01 | | 0.09 | 8.44E-01 | |  | | TUBA1B | -0.44 | 7.52E-03 | | -0.04 | | 9.38E-01 | 0.06 | 9.27E-01 | |
| CBSL | 1.04 | | 8.63E-03 | -0.06 | 9.70E-01 | | -0.27 | 8.00E-01 | |  | | FGD5-AS1 | -0.44 | 7.33E-03 | | -0.30 | | 4.87E-01 | -0.35 | 2.57E-01 | |
| TFE3 | 1.04 | | 6.23E-08 | 0.23 | 6.20E-01 | | 0.35 | 4.09E-01 | |  | | CTSB | -0.44 | 1.11E-03 | | -0.40 | | 2.52E-02 | -0.30 | 3.23E-01 | |
| PPM1M | 1.04 | | 8.35E-06 | 0.73 | 2.00E-02 | | 0.58 | 1.22E-01 | |  | | EED | -0.44 | 6.13E-03 | | 0.10 | | 7.98E-01 | -0.01 | 9.91E-01 | |
| ATF4 | 1.04 | | 1.50E-16 | -0.04 | 9.39E-01 | | -0.02 | 9.71E-01 | |  | | H2AFX | -0.44 | 8.03E-03 | | 0.15 | | 7.49E-01 | 0.28 | 5.85E-01 | |
| CALCOCO2 | 1.04 | | 2.05E-16 | -0.20 | 5.77E-01 | | 0.36 | 1.83E-01 | |  | | MTFMT | -0.45 | 9.91E-03 | | 0.00 | | 9.98E-01 | -0.10 | 8.37E-01 | |
| NOCT | 1.03 | | 1.73E-05 | 0.06 | 9.56E-01 | | 0.11 | 8.92E-01 | |  | | CUL1 | -0.45 | 2.26E-03 | | -0.14 | | 7.48E-01 | -0.20 | 5.74E-01 | |
| TLK2 | 1.03 | | 2.60E-10 | -0.13 | 8.13E-01 | | 0.05 | 9.36E-01 | |  | | MCRS1 | -0.45 | 6.28E-03 | | -0.06 | | 9.16E-01 | 0.01 | 9.89E-01 | |
| PLD6 | 1.03 | | 8.76E-05 | 0.41 | 4.00E-01 | | 0.61 | 1.47E-01 | |  | | DCXR | -0.45 | 3.10E-03 | | -0.08 | | 8.92E-01 | -0.10 | 8.82E-01 | |
| ZNF777 | 1.03 | | 5.66E-11 | 0.21 | 6.29E-01 | | 0.33 | 4.96E-01 | |  | | ALG8 | -0.45 | 4.46E-03 | | 0.05 | | 9.22E-01 | -0.16 | 7.34E-01 | |
| SNHG15 | 1.02 | | 6.17E-17 | 0.23 | 4.74E-01 | | 0.24 | 4.98E-01 | |  | | CKB | -0.45 | 6.13E-03 | | -0.44 | | 1.08E-01 | -0.32 | 5.16E-01 | |
| HOXA11 | 1.02 | | 1.03E-06 | 0.51 | 1.04E-01 | | 0.29 | 5.74E-01 | |  | | POP1 | -0.45 | 7.55E-03 | | 0.27 | | 3.06E-01 | 0.32 | 2.76E-01 | |
| TMEM198 | 1.02 | | 5.15E-03 | 0.12 | 9.30E-01 | | 0.34 | 6.82E-01 | |  | | SLCO3A1 | -0.45 | 8.54E-04 | | -0.37 | | 5.36E-02 | -0.26 | 5.14E-01 | |
| MXI1 | 1.02 | | 1.72E-06 | 0.19 | 7.41E-01 | | 0.18 | 7.65E-01 | |  | | PPIA | -0.45 | 7.16E-03 | | 0.01 | | 9.80E-01 | -0.03 | 9.65E-01 | |
| GPCPD1 | 1.01 | | 4.82E-05 | 0.12 | 8.72E-01 | | 0.24 | 7.35E-01 | |  | | TSEN54 | -0.45 | 1.97E-03 | | 0.02 | | 9.68E-01 | -0.01 | 9.87E-01 | |
| PALB2 | 1.01 | | 2.25E-09 | 0.12 | 8.10E-01 | | 0.22 | 6.34E-01 | |  | | TMEM51 | -0.45 | 9.38E-03 | | -0.14 | | 7.12E-01 | -0.16 | 7.13E-01 | |
| NR4A1 | 1.01 | | 2.07E-03 | 0.11 | 9.29E-01 | | 0.72 | 1.70E-01 | |  | | DHCR24 | -0.45 | 6.29E-03 | | -0.41 | | 7.33E-02 | -0.15 | 7.45E-01 | |
| RAPH1 | 1.01 | | 5.21E-04 | -0.08 | 9.40E-01 | | 0.39 | 4.81E-01 | |  | | RRM2 | -0.45 | 4.25E-03 | | -0.04 | | 9.51E-01 | 0.09 | 8.59E-01 | |
| THSD1 | 1.01 | | 1.43E-06 | 0.03 | 9.71E-01 | | 0.31 | 5.60E-01 | |  | | DAZAP2 | -0.45 | 3.34E-04 | | -0.29 | | 2.20E-01 | -0.19 | 5.47E-01 | |
| HSPA13 | 1.00 | | 1.55E-07 | 0.02 | 9.83E-01 | | 0.27 | 7.02E-01 | |  | | ELOVL5 | -0.45 | 3.43E-03 | | -0.05 | | 9.41E-01 | -0.20 | 7.03E-01 | |
| B3GNT10 | 1.00 | | 6.06E-08 | 0.12 | 8.59E-01 | | 0.30 | 5.35E-01 | |  | | CD9 | -0.45 | 5.07E-04 | | -0.16 | | 6.09E-01 | -0.16 | 6.38E-01 | |
| PER2 | 1.00 | | 3.34E-09 | 0.25 | 5.45E-01 | | 0.15 | 7.82E-01 | |  | | GGA2 | -0.45 | 7.41E-03 | | -0.28 | | 3.59E-01 | -0.35 | 2.74E-01 | |
| ZNF181 | 1.00 | | 2.35E-03 | -0.18 | 8.61E-01 | | -0.17 | 8.63E-01 | |  | | LMNB2 | -0.45 | 8.07E-04 | | -0.27 | | 2.32E-01 | -0.10 | 8.34E-01 | |
| PYROXD1 | 1.00 | | 1.06E-09 | 0.06 | 9.33E-01 | | 0.08 | 8.93E-01 | |  | | TM9SF4 | -0.45 | 3.79E-03 | | 0.06 | | 9.02E-01 | 0.07 | 8.91E-01 | |
| PDLIM2 | 1.00 | | 1.19E-16 | 0.25 | 4.83E-01 | | 0.50 | 1.02E-01 | |  | | ZDHHC13 | -0.45 | 9.39E-04 | | -0.15 | | 7.53E-01 | -0.10 | 8.68E-01 | |
| ZFP41 | 0.99 | | 1.09E-04 | 0.38 | 4.55E-01 | | 0.66 | 1.29E-01 | |  | | DONSON | -0.46 | 3.59E-03 | | -0.28 | | 3.03E-01 | -0.39 | 2.24E-01 | |
| ZNF202 | 0.99 | | 2.19E-10 | 0.10 | 8.46E-01 | | 0.20 | 6.33E-01 | |  | | UQCC1 | -0.46 | 5.66E-03 | | -0.03 | | 9.54E-01 | -0.01 | 9.86E-01 | |
| HIVEP2 | 0.99 | | 4.74E-05 | -0.27 | 6.26E-01 | | 0.20 | 7.34E-01 | |  | | ACAD8 | -0.46 | 8.22E-03 | | 0.15 | | 7.05E-01 | 0.07 | 8.89E-01 | |
| ARL14 | 0.99 | | 5.60E-07 | 0.60 | 2.44E-02 | | 0.28 | 5.38E-01 | |  | | UBE2G2 | -0.46 | 4.15E-03 | | -0.01 | | 9.87E-01 | -0.12 | 7.89E-01 | |
| KLF5 | 0.99 | | 6.00E-15 | -0.01 | 9.78E-01 | | 0.14 | 7.15E-01 | |  | | SLC35B2 | -0.46 | 2.74E-03 | | 0.05 | | 9.24E-01 | -0.02 | 9.78E-01 | |
| F2R | 0.99 | | 7.83E-07 | 0.21 | 7.11E-01 | | 0.14 | 7.88E-01 | |  | | MRPL43 | -0.46 | 5.10E-03 | | -0.21 | | 5.39E-01 | -0.29 | 4.52E-01 | |
| ZSCAN21 | 0.99 | | 5.50E-04 | 0.19 | 8.18E-01 | | 0.36 | 5.54E-01 | |  | | BID | -0.46 | 1.52E-03 | | 0.00 | | 9.96E-01 | -0.05 | 9.40E-01 | |
| DUSP16 | 0.99 | | 2.01E-03 | -0.12 | 9.17E-01 | | -0.10 | 9.17E-01 | |  | | IL1RN | -0.46 | 1.30E-03 | | -0.19 | | 5.38E-01 | -0.34 | 2.60E-01 | |
| ZNF354A | 0.99 | | 1.81E-06 | 0.11 | 8.79E-01 | | -0.13 | 8.51E-01 | |  | | ZFYVE21 | -0.46 | 2.41E-03 | | -0.12 | | 7.64E-01 | -0.07 | 8.93E-01 | |
| USP25 | 0.98 | | 2.23E-08 | -0.21 | 6.83E-01 | | -0.01 | 9.92E-01 | |  | | CCDC115 | -0.46 | 9.34E-03 | | -0.45 | | 5.69E-02 | -0.44 | 1.50E-01 | |
| MXRA5 | 0.98 | | 7.35E-09 | -1.04 | 1.82E-08 | | 0.10 | 8.35E-01 | |  | | ACTR1A | -0.46 | 9.39E-04 | | 0.02 | | 9.67E-01 | 0.05 | 9.30E-01 | |
| SCML1 | 0.98 | | 6.59E-07 | 0.40 | 2.45E-01 | | 0.48 | 1.82E-01 | |  | | TUBA4A | -0.46 | 1.26E-03 | | -0.26 | | 3.29E-01 | -0.12 | 8.22E-01 | |
| POLR3C | 0.97 | | 7.35E-13 | -0.14 | 7.57E-01 | | 0.02 | 9.72E-01 | |  | | KIF20B | -0.46 | 6.30E-03 | | -0.14 | | 7.35E-01 | -0.57 | 1.15E-01 | |
| NAMPT | 0.97 | | 7.36E-16 | 0.24 | 4.83E-01 | | 0.42 | 2.90E-01 | |  | | PIDD1 | -0.46 | 6.21E-03 | | -0.04 | | 9.54E-01 | 0.06 | 9.40E-01 | |
| ZC2HC1A | 0.96 | | 1.64E-04 | -0.24 | 7.57E-01 | | -0.15 | 8.81E-01 | |  | | CGN | -0.46 | 7.45E-03 | | -0.25 | | 4.44E-01 | 0.28 | 4.46E-01 | |
| TRIM39 | 0.96 | | 9.11E-05 | -0.05 | 9.64E-01 | | 0.18 | 8.00E-01 | |  | | SLC35B4 | -0.46 | 7.31E-03 | | 0.34 | | 1.41E-01 | 0.15 | 7.40E-01 | |
| FAM86EP | 0.96 | | 1.53E-05 | 0.05 | 9.60E-01 | | -0.06 | 9.40E-01 | |  | | TCERG1 | -0.46 | 5.63E-04 | | -0.16 | | 6.23E-01 | -0.24 | 4.35E-01 | |
| PNRC1 | 0.96 | | 1.36E-04 | -0.54 | 2.48E-01 | | -0.65 | 1.58E-01 | |  | | NUDT1 | -0.47 | 7.40E-03 | | -0.02 | | 9.72E-01 | -0.03 | 9.65E-01 | |
| MSANTD4 | 0.96 | | 2.04E-06 | 0.00 | 9.99E-01 | | 0.03 | 9.69E-01 | |  | | HMGB1 | -0.47 | 2.07E-03 | | -0.16 | | 5.98E-01 | -0.35 | 2.30E-01 | |
| TAF4B | 0.96 | | 3.41E-07 | 0.05 | 9.42E-01 | | 0.31 | 4.06E-01 | |  | | SLC25A1 | -0.47 | 6.05E-04 | | -0.33 | | 1.74E-01 | -0.29 | 4.76E-01 | |
| ZC3H8 | 0.95 | | 3.74E-07 | 0.38 | 2.07E-01 | | 0.11 | 8.85E-01 | |  | | UBR7 | -0.47 | 2.74E-03 | | -0.21 | | 6.40E-01 | -0.09 | 8.56E-01 | |
| UPF3B | 0.95 | | 1.16E-07 | -0.05 | 9.44E-01 | | -0.36 | 4.06E-01 | |  | | RAB13 | -0.47 | 6.21E-03 | | -0.13 | | 7.71E-01 | -0.40 | 1.73E-01 | |
| BCL10 | 0.95 | | 1.88E-08 | 0.46 | 8.91E-02 | | 0.50 | 2.22E-01 | |  | | MANEAL | -0.47 | 6.35E-03 | | -0.03 | | 9.60E-01 | -0.13 | 7.89E-01 | |
| WASL | 0.95 | | 6.80E-10 | 0.43 | 1.47E-01 | | 0.48 | 1.99E-01 | |  | | NDRG3 | -0.47 | 9.18E-04 | | -0.24 | | 3.38E-01 | -0.33 | 2.16E-01 | |
| RASSF10-DT | 0.95 | | 1.13E-03 | 0.06 | 9.59E-01 | | -0.08 | 9.36E-01 | |  | | SLC30A5 | -0.47 | 6.33E-03 | | 0.00 | | 9.96E-01 | -0.15 | 7.71E-01 | |
| CHN1 | 0.95 | | 2.82E-04 | -0.26 | 7.28E-01 | | 0.18 | 7.91E-01 | |  | | AKT1 | -0.47 | 1.22E-03 | | 0.18 | | 6.29E-01 | 0.33 | 3.74E-01 | |
| PYCR1 | 0.95 | | 3.88E-09 | -0.05 | 9.44E-01 | | 0.00 | 9.98E-01 | |  | | ARV1 | -0.47 | 2.93E-03 | | -0.10 | | 8.37E-01 | -0.22 | 6.56E-01 | |
| TRIM36 | 0.95 | | 3.28E-04 | -0.19 | 8.30E-01 | | -0.28 | 6.93E-01 | |  | | PTMA | -0.47 | 5.07E-03 | | -0.03 | | 9.59E-01 | 0.13 | 7.54E-01 | |
| ZNF134 | 0.95 | | 4.95E-06 | -0.25 | 6.19E-01 | | -0.34 | 4.75E-01 | |  | | LAMC1 | -0.47 | 9.50E-03 | | -0.21 | | 5.85E-01 | -0.05 | 9.32E-01 | |
| FGD6 | 0.94 | | 7.08E-10 | 0.14 | 7.73E-01 | | 0.43 | 2.47E-01 | |  | | FOXA1 | -0.47 | 4.00E-03 | | -0.31 | | 2.70E-01 | -0.38 | 2.35E-01 | |
| ZNF518A | 0.94 | | 9.64E-07 | -0.08 | 9.21E-01 | | -0.01 | 9.92E-01 | |  | | TECR | -0.47 | 1.74E-03 | | -0.14 | | 7.15E-01 | -0.27 | 4.22E-01 | |
| EPB41L4A-AS1 | 0.94 | | 1.15E-05 | 0.16 | 8.29E-01 | | 0.07 | 9.32E-01 | |  | | WRAP53 | -0.47 | 2.38E-03 | | 0.00 | | 9.96E-01 | 0.17 | 7.39E-01 | |
| PKD1P6 | 0.94 | | 8.44E-03 | 0.09 | 9.45E-01 | | 0.50 | 4.60E-01 | |  | | CCNB1 | -0.48 | 5.10E-04 | | -0.21 | | 4.14E-01 | -0.29 | 3.07E-01 | |
| GTF2IP20 | 0.94 | | 1.56E-03 | 0.33 | 6.29E-01 | | 0.42 | 4.55E-01 | |  | | GID8 | -0.48 | 6.59E-04 | | -0.13 | | 7.17E-01 | -0.24 | 4.88E-01 | |
| ZNF438 | 0.94 | | 7.75E-03 | 0.06 | 9.63E-01 | | 0.23 | 7.94E-01 | |  | | VSIG10 | -0.48 | 1.82E-03 | | -0.24 | | 3.92E-01 | -0.43 | 1.17E-01 | |
| COX19 | 0.94 | | 5.45E-13 | 0.02 | 9.76E-01 | | -0.14 | 7.36E-01 | |  | | POLD1 | -0.48 | 1.18E-03 | | 0.03 | | 9.68E-01 | 0.07 | 9.22E-01 | |
| PROSER2 | 0.93 | | 1.84E-12 | 0.09 | 8.85E-01 | | 0.30 | 4.33E-01 | |  | | TSN | -0.48 | 9.82E-04 | | -0.30 | | 3.15E-01 | -0.38 | 2.30E-01 | |
| ZNF562 | 0.93 | | 1.77E-07 | 0.24 | 5.34E-01 | | 0.40 | 3.43E-01 | |  | | AIMP2 | -0.48 | 1.24E-03 | | -0.06 | | 9.15E-01 | -0.12 | 7.98E-01 | |
| STOML1 | 0.93 | | 2.39E-10 | -0.18 | 7.08E-01 | | 0.28 | 5.47E-01 | |  | | MALSU1 | -0.48 | 5.45E-03 | | 0.04 | | 9.52E-01 | -0.07 | 8.90E-01 | |
| SDSL | 0.93 | | 3.16E-07 | 0.18 | 7.60E-01 | | 0.52 | 1.71E-01 | |  | | SAFB2 | -0.48 | 4.09E-03 | | 0.03 | | 9.67E-01 | 0.09 | 8.56E-01 | |
| JMY | 0.93 | | 2.05E-05 | -0.19 | 7.58E-01 | | -0.38 | 4.11E-01 | |  | | FANCA | -0.48 | 4.80E-03 | | -0.17 | | 6.65E-01 | -0.17 | 6.77E-01 | |
| KLF13 | 0.93 | | 1.84E-03 | 0.44 | 4.96E-01 | | 0.58 | 2.79E-01 | |  | | ORC1 | -0.48 | 8.50E-03 | | -0.20 | | 5.74E-01 | -0.05 | 9.39E-01 | |
| MDK | 0.93 | | 1.46E-12 | -0.49 | 6.72E-02 | | 0.34 | 4.11E-01 | |  | | WDR77 | -0.48 | 9.14E-04 | | 0.10 | | 7.84E-01 | 0.12 | 7.90E-01 | |
| GORAB | 0.93 | | 5.04E-06 | -0.08 | 9.32E-01 | | -0.09 | 9.07E-01 | |  | | STIP1 | -0.48 | 6.96E-04 | | -0.06 | | 8.85E-01 | -0.03 | 9.50E-01 | |
| DUS4L | 0.93 | | 3.32E-08 | 0.38 | 2.47E-01 | | 0.36 | 4.13E-01 | |  | | DHRS1 | -0.48 | 8.22E-03 | | -0.22 | | 4.93E-01 | -0.06 | 9.20E-01 | |
| SH3RF2 | 0.93 | | 7.59E-11 | -0.04 | 9.41E-01 | | -0.20 | 5.90E-01 | |  | | FN3KRP | -0.48 | 9.80E-04 | | -0.21 | | 5.24E-01 | -0.27 | 4.16E-01 | |
| SNHG17 | 0.93 | | 4.41E-12 | 0.21 | 5.34E-01 | | 0.22 | 6.37E-01 | |  | | PODXL | -0.48 | 3.05E-03 | | -0.21 | | 5.50E-01 | -0.15 | 7.26E-01 | |
| TBC1D9 | 0.92 | | 1.57E-04 | -0.45 | 3.70E-01 | | 0.21 | 7.28E-01 | |  | | AK3 | -0.48 | 1.98E-03 | | -0.24 | | 6.44E-01 | -0.42 | 2.38E-01 | |
| ODF2L | 0.92 | | 1.89E-06 | 0.27 | 5.29E-01 | | 0.49 | 2.94E-01 | |  | | ZDHHC3 | -0.48 | 2.15E-04 | | -0.33 | | 1.25E-01 | -0.35 | 1.70E-01 | |
| SARS | 0.92 | | 1.20E-12 | -0.10 | 8.06E-01 | | -0.13 | 7.62E-01 | |  | | RIPK4 | -0.48 | 3.40E-03 | | -0.60 | | 9.26E-03 | -0.49 | 1.31E-01 | |
| ZNF426 | 0.92 | | 1.12E-07 | 0.09 | 8.81E-01 | | 0.13 | 8.10E-01 | |  | | ACTG1 | -0.48 | 1.40E-04 | | -0.20 | | 5.39E-01 | -0.11 | 8.36E-01 | |
| ZNF701 | 0.92 | | 1.78E-03 | -0.13 | 8.92E-01 | | 0.20 | 7.95E-01 | |  | | PLD2 | -0.48 | 1.75E-03 | | -0.27 | | 3.06E-01 | -0.12 | 8.11E-01 | |
| NAMPTP1 | 0.91 | | 2.02E-04 | 0.13 | 8.75E-01 | | 0.31 | 6.14E-01 | |  | | CBR1 | -0.48 | 8.38E-03 | | -0.09 | | 8.14E-01 | 0.07 | 8.77E-01 | |
| TMCO4 | 0.91 | | 4.05E-07 | -0.04 | 9.55E-01 | | 0.49 | 1.69E-01 | |  | | HAT1 | -0.48 | 2.23E-04 | | -0.21 | | 4.53E-01 | -0.39 | 2.71E-01 | |
| PATL1 | 0.91 | | 5.74E-08 | 0.13 | 8.01E-01 | | 0.46 | 1.40E-01 | |  | | TM9SF2 | -0.48 | 1.14E-04 | | -0.21 | | 6.13E-01 | -0.13 | 8.31E-01 | |
| ZFAND3 | 0.91 | | 1.03E-03 | 0.25 | 6.94E-01 | | 0.35 | 5.47E-01 | |  | | MTCH2 | -0.49 | 6.45E-04 | | -0.12 | | 7.40E-01 | -0.31 | 2.43E-01 | |
| LINC00339 | 0.91 | | 6.01E-05 | 0.08 | 9.29E-01 | | 0.01 | 9.93E-01 | |  | | PWWP3A | -0.49 | 7.05E-03 | | 0.01 | | 9.90E-01 | -0.08 | 8.88E-01 | |
| RPL22L1 | 0.91 | | 3.66E-05 | 0.48 | 1.53E-01 | | 0.52 | 2.65E-01 | |  | | IFT122 | -0.49 | 7.13E-03 | | -0.36 | | 1.98E-01 | -0.36 | 2.48E-01 | |
| ARHGEF37 | 0.91 | | 1.48E-07 | -0.42 | 8.38E-02 | | -0.06 | 9.18E-01 | |  | | STX10 | -0.49 | 2.77E-03 | | -0.04 | | 9.50E-01 | -0.03 | 9.58E-01 | |
| SH2D3A | 0.90 | | 2.54E-11 | 0.39 | 1.20E-01 | | 0.43 | 2.74E-01 | |  | | CLK3 | -0.49 | 9.53E-04 | | 0.02 | | 9.76E-01 | 0.06 | 9.20E-01 | |
| LTO1 | 0.90 | | 9.96E-09 | -0.06 | 9.29E-01 | | -0.07 | 8.98E-01 | |  | | TMEM30A | -0.49 | 4.43E-03 | | -0.14 | | 8.35E-01 | -0.24 | 6.49E-01 | |
| ZNF641 | 0.90 | | 6.01E-06 | 0.20 | 7.20E-01 | | 0.20 | 6.98E-01 | |  | | RAB34 | -0.49 | 2.74E-04 | | -0.02 | | 9.76E-01 | -0.12 | 7.92E-01 | |
| CBLB | 0.90 | | 1.30E-04 | -0.66 | 2.80E-02 | | -0.56 | 1.50E-01 | |  | | GRWD1 | -0.49 | 1.64E-03 | | 0.13 | | 7.52E-01 | 0.35 | 3.62E-01 | |
| EIF4EBP1 | 0.90 | | 7.69E-13 | 0.57 | 8.78E-03 | | 0.50 | 1.25E-01 | |  | | CDKN2AIPNL | -0.49 | 3.87E-03 | | -0.04 | | 9.55E-01 | -0.25 | 4.79E-01 | |
| NCOA7 | 0.90 | | 4.70E-09 | -0.76 | 3.67E-04 | | -0.47 | 2.40E-01 | |  | | SLC25A4 | -0.49 | 7.63E-04 | | 0.05 | | 9.21E-01 | 0.04 | 9.36E-01 | |
| CAMK2D | 0.90 | | 1.24E-04 | -0.20 | 7.74E-01 | | 0.28 | 6.23E-01 | |  | | ITGAE | -0.49 | 9.80E-03 | | 0.01 | | 9.93E-01 | -0.47 | 1.33E-01 | |
| ZBTB37 | 0.90 | | 1.47E-03 | -0.15 | 8.65E-01 | | 0.20 | 7.98E-01 | |  | | PSMD10 | -0.49 | 9.25E-04 | | -0.02 | | 9.72E-01 | -0.12 | 8.10E-01 | |
| CHIC2 | 0.90 | | 2.32E-06 | 0.24 | 5.90E-01 | | 0.12 | 8.57E-01 | |  | | HSPH1 | -0.49 | 2.01E-04 | | 0.07 | | 8.88E-01 | 0.12 | 8.08E-01 | |
| BNC1 | 0.90 | | 2.42E-09 | 0.08 | 8.94E-01 | | 0.28 | 3.76E-01 | |  | | CERT1 | -0.49 | 2.65E-03 | | -0.35 | | 1.84E-01 | -0.44 | 1.13E-01 | |
| MINDY2 | 0.90 | | 1.22E-07 | 0.08 | 8.76E-01 | | -0.02 | 9.74E-01 | |  | | DSCC1 | -0.49 | 4.18E-03 | | 0.03 | | 9.60E-01 | -0.14 | 7.88E-01 | |
| RP9 | 0.89 | | 1.20E-05 | 0.28 | 5.89E-01 | | 0.05 | 9.52E-01 | |  | | C1QTNF6 | -0.49 | 4.79E-03 | | 0.13 | | 7.78E-01 | -0.04 | 9.46E-01 | |
| ZNF44 | 0.89 | | 1.34E-04 | -0.54 | 1.71E-01 | | -0.47 | 3.01E-01 | |  | | MTERF1 | -0.49 | 8.80E-03 | | -0.09 | | 8.87E-01 | -0.22 | 7.13E-01 | |
| PKD1 | 0.89 | | 9.49E-04 | 0.29 | 5.65E-01 | | 0.39 | 4.36E-01 | |  | | H2AFZ | -0.49 | 5.98E-04 | | 0.12 | | 7.88E-01 | -0.11 | 8.09E-01 | |
| SRGAP1 | 0.89 | | 3.64E-08 | 0.23 | 4.34E-01 | | 0.00 | 9.93E-01 | |  | | SMARCA1 | -0.49 | 7.90E-03 | | 0.06 | | 9.27E-01 | -0.18 | 7.46E-01 | |
| AXIN1 | 0.89 | | 4.46E-04 | 0.43 | 3.35E-01 | | 0.57 | 1.98E-01 | |  | | JMJD8 | -0.49 | 1.40E-03 | | -0.06 | | 9.26E-01 | -0.07 | 9.19E-01 | |
| RAB11FIP1 | 0.89 | | 7.81E-07 | 0.14 | 7.78E-01 | | 0.33 | 3.43E-01 | |  | | MEN1 | -0.50 | 3.34E-04 | | 0.02 | | 9.67E-01 | -0.01 | 9.84E-01 | |
| RIMKLB | 0.88 | | 5.96E-06 | 0.43 | 1.06E-01 | | -0.02 | 9.78E-01 | |  | | NOMO1 | -0.50 | 4.82E-03 | | -0.01 | | 9.93E-01 | 0.04 | 9.52E-01 | |
| ZNF205 | 0.88 | | 4.96E-06 | 0.10 | 8.85E-01 | | 0.43 | 3.91E-01 | |  | | PFDN5 | -0.50 | 5.28E-03 | | 0.13 | | 7.93E-01 | 0.10 | 8.56E-01 | |
| HOXA1 | 0.88 | | 9.55E-07 | 0.49 | 5.39E-02 | | 0.44 | 2.09E-01 | |  | | PPARG | -0.50 | 2.56E-03 | | 0.22 | | 5.08E-01 | -0.35 | 2.47E-01 | |
| BCLAF3 | 0.88 | | 4.67E-05 | 0.12 | 8.59E-01 | | 0.28 | 6.80E-01 | |  | | LGR4 | -0.50 | 6.89E-03 | | -0.55 | | 5.58E-02 | -0.27 | 5.72E-01 | |
| CLCA2 | 0.88 | | 3.06E-08 | -0.05 | 9.46E-01 | | 0.19 | 7.19E-01 | |  | | RBBP7 | -0.50 | 2.75E-04 | | -0.29 | | 2.68E-01 | -0.29 | 3.18E-01 | |
| LRIF1 | 0.88 | | 2.65E-06 | 0.08 | 9.10E-01 | | 0.17 | 8.20E-01 | |  | | CDK5 | -0.50 | 6.26E-03 | | -0.01 | | 9.93E-01 | -0.10 | 8.60E-01 | |
| SCAMP1-AS1 | 0.88 | | 1.84E-03 | 0.08 | 9.41E-01 | | 0.29 | 6.70E-01 | |  | | SLC25A5 | -0.50 | 2.05E-03 | | -0.12 | | 7.64E-01 | -0.11 | 7.88E-01 | |
| RIOK3 | 0.88 | | 9.96E-08 | -0.07 | 8.99E-01 | | -0.05 | 9.36E-01 | |  | | CNP | -0.50 | 5.28E-04 | | -0.14 | | 6.93E-01 | 0.00 | 9.97E-01 | |
| R3HDM2 | 0.88 | | 6.19E-05 | 0.38 | 3.12E-01 | | 0.40 | 3.84E-01 | |  | | TFDP1 | -0.50 | 1.92E-04 | | -0.07 | | 8.66E-01 | -0.10 | 8.10E-01 | |
| SMG9 | 0.87 | | 2.77E-05 | 0.09 | 9.09E-01 | | 0.11 | 8.64E-01 | |  | | HAUS8 | -0.50 | 6.89E-03 | | -0.33 | | 2.35E-01 | -0.21 | 6.15E-01 | |
| YARS | 0.87 | | 7.74E-11 | -0.10 | 8.17E-01 | | -0.12 | 7.82E-01 | |  | | FGFBP1 | -0.50 | 6.53E-04 | | -0.10 | | 7.94E-01 | 0.04 | 9.46E-01 | |
| HERPUD1 | 0.87 | | 2.65E-10 | -0.36 | 1.83E-01 | | -0.39 | 1.28E-01 | |  | | CETN2 | -0.50 | 1.15E-03 | | -0.15 | | 7.08E-01 | -0.39 | 1.54E-01 | |
| ZSCAN26 | 0.87 | | 1.34E-03 | -0.10 | 9.26E-01 | | 0.08 | 9.36E-01 | |  | | RPL29 | -0.50 | 3.45E-03 | | 0.26 | | 5.53E-01 | 0.16 | 7.57E-01 | |
| SNHG8 | 0.87 | | 1.95E-05 | 0.30 | 5.22E-01 | | 0.15 | 7.98E-01 | |  | | USPL1 | -0.50 | 5.00E-03 | | -0.17 | | 7.18E-01 | -0.27 | 6.18E-01 | |
| SLC43A1 | 0.87 | | 4.43E-03 | 0.37 | 5.51E-01 | | -0.04 | 9.65E-01 | |  | | HS6ST1 | -0.50 | 7.52E-04 | | 0.03 | | 9.54E-01 | 0.27 | 4.85E-01 | |
| AKR1C2 | 0.87 | | 2.07E-07 | 0.10 | 8.46E-01 | | 0.26 | 5.17E-01 | |  | | MOGS | -0.50 | 2.24E-04 | | 0.21 | | 5.56E-01 | 0.15 | 7.54E-01 | |
| SETDB2 | 0.87 | | 4.25E-05 | -0.06 | 9.47E-01 | | 0.19 | 7.87E-01 | |  | | OPA1 | -0.50 | 3.77E-03 | | -0.35 | | 3.10E-01 | -0.48 | 1.68E-01 | |
| ZNF263 | 0.87 | | 1.99E-08 | -0.08 | 8.74E-01 | | 0.11 | 8.20E-01 | |  | | NPEPPS | -0.50 | 1.38E-04 | | -0.20 | | 6.00E-01 | -0.29 | 4.22E-01 | |
| CERS3 | 0.86 | | 6.42E-07 | -0.48 | 9.84E-02 | | 0.17 | 7.28E-01 | |  | | ARPC5 | -0.50 | 7.06E-04 | | -0.10 | | 8.32E-01 | -0.17 | 7.57E-01 | |
| ZNF550 | 0.86 | | 1.84E-03 | -0.05 | 9.65E-01 | | 0.11 | 8.97E-01 | |  | | ST14 | -0.51 | 1.91E-03 | | -0.28 | | 3.20E-01 | 0.03 | 9.65E-01 | |
| KRCC1 | 0.86 | | 2.06E-07 | -0.17 | 7.53E-01 | | -0.17 | 7.87E-01 | |  | | RPS3 | -0.51 | 3.99E-04 | | -0.07 | | 8.53E-01 | -0.07 | 8.98E-01 | |
| CSGALNACT2 | 0.86 | | 3.30E-06 | 0.16 | 7.77E-01 | | 0.05 | 9.49E-01 | |  | | PRADC1 | -0.51 | 5.53E-03 | | -0.27 | | 4.49E-01 | -0.43 | 2.23E-01 | |
| RNF146 | 0.86 | | 2.48E-07 | -0.17 | 7.33E-01 | | -0.13 | 8.26E-01 | |  | | MESD | -0.51 | 1.36E-04 | | 0.04 | | 9.37E-01 | -0.01 | 9.85E-01 | |
| LATS2 | 0.86 | | 8.35E-04 | -0.18 | 8.11E-01 | | -0.27 | 6.67E-01 | |  | | POM121 | -0.51 | 3.41E-03 | | 0.17 | | 6.54E-01 | 0.23 | 5.57E-01 | |
| BMP2K | 0.86 | | 4.89E-04 | 0.62 | 8.30E-02 | | 0.63 | 1.00E-01 | |  | | ZSWIM1 | -0.51 | 8.01E-03 | | -0.23 | | 5.60E-01 | -0.22 | 6.07E-01 | |
| AGTPBP1 | 0.86 | | 1.48E-03 | -0.10 | 9.19E-01 | | -0.16 | 8.35E-01 | |  | | NUDT16L1 | -0.51 | 1.08E-03 | | -0.14 | | 7.69E-01 | -0.14 | 8.15E-01 | |
| POLR3D | 0.85 | | 2.21E-09 | 0.07 | 8.94E-01 | | 0.37 | 2.03E-01 | |  | | PANK4 | -0.51 | 9.65E-03 | | 0.12 | | 7.81E-01 | 0.23 | 6.01E-01 | |
| CHST3 | 0.85 | | 4.18E-07 | -0.03 | 9.65E-01 | | 0.20 | 6.59E-01 | |  | | CDC20 | -0.51 | 2.17E-04 | | -0.03 | | 9.47E-01 | 0.05 | 9.32E-01 | |
| RNF214 | 0.85 | | 8.99E-06 | 0.33 | 3.17E-01 | | 0.22 | 6.37E-01 | |  | | ENDOG | -0.51 | 4.79E-03 | | 0.00 | | 9.97E-01 | 0.12 | 8.64E-01 | |
| NEDD4L | 0.85 | | 1.12E-07 | 0.41 | 5.29E-02 | | 0.30 | 3.38E-01 | |  | | AREL1 | -0.51 | 7.36E-03 | | -0.02 | | 9.74E-01 | 0.24 | 5.67E-01 | |
| CCDC174 | 0.85 | | 5.67E-04 | 0.13 | 8.57E-01 | | -0.13 | 8.51E-01 | |  | | CHEK2 | -0.51 | 7.22E-03 | | -0.28 | | 5.29E-01 | -0.31 | 4.01E-01 | |
| OXSR1 | 0.85 | | 5.32E-08 | 0.23 | 4.85E-01 | | 0.33 | 3.31E-01 | |  | | QARS | -0.51 | 5.32E-04 | | -0.33 | | 1.29E-01 | -0.26 | 4.98E-01 | |
| NFXL1 | 0.85 | | 9.64E-07 | 0.05 | 9.47E-01 | | 0.30 | 4.40E-01 | |  | | ZWINT | -0.51 | 1.24E-04 | | -0.21 | | 4.35E-01 | -0.15 | 6.79E-01 | |
| TMCC1 | 0.85 | | 7.22E-07 | -0.23 | 5.79E-01 | | -0.09 | 8.73E-01 | |  | | WDR34 | -0.51 | 3.45E-04 | | -0.36 | | 1.05E-01 | -0.35 | 4.27E-01 | |
| EPC2 | 0.85 | | 6.43E-03 | 0.22 | 7.79E-01 | | -0.08 | 9.36E-01 | |  | | EIF4EBP2 | -0.51 | 6.05E-04 | | -0.13 | | 7.61E-01 | -0.11 | 8.17E-01 | |
| TNFRSF12A | 0.84 | | 5.60E-12 | 0.23 | 5.74E-01 | | 0.40 | 2.13E-01 | |  | | DYNLT1 | -0.51 | 6.18E-03 | | -0.13 | | 7.40E-01 | -0.23 | 4.98E-01 | |
| FOXE1 | 0.84 | | 8.34E-07 | 0.22 | 5.29E-01 | | 0.08 | 8.86E-01 | |  | | MCM10 | -0.51 | 1.56E-04 | | -0.02 | | 9.72E-01 | -0.06 | 8.99E-01 | |
| PDRG1 | 0.84 | | 1.69E-09 | -0.01 | 9.90E-01 | | -0.02 | 9.78E-01 | |  | | TUSC2 | -0.51 | 3.60E-03 | | 0.14 | | 7.59E-01 | 0.22 | 6.55E-01 | |
| TAOK3 | 0.84 | | 1.64E-09 | 0.19 | 5.72E-01 | | 0.40 | 1.17E-01 | |  | | ATAD5 | -0.51 | 8.88E-03 | | -0.04 | | 9.59E-01 | -0.18 | 7.07E-01 | |
| LENG9 | 0.84 | | 6.98E-03 | 0.60 | 2.93E-01 | | 0.69 | 2.00E-01 | |  | | HNRNPAB | -0.51 | 1.67E-03 | | -0.09 | | 8.52E-01 | -0.07 | 8.98E-01 | |
| C1orf116 | 0.84 | | 1.88E-08 | 0.05 | 9.26E-01 | | 0.17 | 6.62E-01 | |  | | JAG2 | -0.52 | 3.50E-03 | | -0.37 | | 2.20E-01 | -0.58 | 1.44E-01 | |
| FAM110A | 0.84 | | 2.89E-08 | 0.02 | 9.76E-01 | | 0.38 | 3.34E-01 | |  | | RPS15 | -0.52 | 1.58E-03 | | 0.21 | | 6.09E-01 | 0.29 | 5.33E-01 | |
| ZNF670 | 0.84 | | 8.24E-06 | 0.00 | 9.98E-01 | | -0.06 | 9.37E-01 | |  | | ARL6IP1 | -0.52 | 2.10E-05 | | -0.31 | | 1.88E-01 | -0.40 | 2.55E-01 | |
| ZFP36L1 | 0.84 | | 2.71E-06 | 0.22 | 6.18E-01 | | 0.21 | 6.67E-01 | |  | | RAB5B | -0.52 | 3.26E-03 | | -0.32 | | 3.51E-01 | -0.38 | 2.00E-01 | |
| PARD3 | 0.84 | | 2.05E-07 | 0.08 | 8.91E-01 | | 0.38 | 2.24E-01 | |  | | PPME1 | -0.52 | 1.06E-04 | | -0.30 | | 1.53E-01 | -0.23 | 4.92E-01 | |
| CCNB1IP1 | 0.84 | | 9.57E-09 | 0.10 | 8.35E-01 | | -0.07 | 9.07E-01 | |  | | ADD3 | -0.52 | 4.37E-03 | | -0.27 | | 4.25E-01 | -0.41 | 2.43E-01 | |
| SRPX | 0.84 | | 6.27E-03 | 0.72 | 1.04E-01 | | 0.43 | 4.98E-01 | |  | | FAT2 | -0.52 | 2.66E-03 | | -0.42 | | 4.14E-02 | -0.37 | 1.58E-01 | |
| HIVEP1 | 0.84 | | 1.91E-05 | -0.37 | 3.68E-01 | | -0.03 | 9.69E-01 | |  | | MICOS10 | -0.52 | 4.77E-03 | | 0.07 | | 9.09E-01 | -0.20 | 7.62E-01 | |
| CCNG2 | 0.83 | | 2.15E-03 | 0.58 | 1.89E-01 | | 0.28 | 6.48E-01 | |  | | BUB1B | -0.52 | 3.10E-04 | | -0.22 | | 5.18E-01 | -0.22 | 6.01E-01 | |
| CSRNP2 | 0.83 | | 6.09E-07 | -0.21 | 6.16E-01 | | 0.12 | 7.94E-01 | |  | | CCT3 | -0.52 | 2.83E-04 | | -0.04 | | 9.42E-01 | -0.10 | 8.25E-01 | |
| CSNK1G1 | 0.83 | | 3.02E-08 | -0.05 | 9.41E-01 | | 0.03 | 9.58E-01 | |  | | CHKA | -0.52 | 3.66E-03 | | 0.03 | | 9.64E-01 | -0.33 | 3.16E-01 | |
| APBB3 | 0.83 | | 2.30E-03 | 0.61 | 7.97E-02 | | 0.53 | 2.27E-01 | |  | | SLC25A12 | -0.52 | 1.46E-03 | | -0.16 | | 7.20E-01 | -0.26 | 4.91E-01 | |
| IL15 | 0.83 | | 9.66E-03 | -1.67 | 2.08E-04 | | -0.05 | 9.61E-01 | |  | | ZDHHC16 | -0.52 | 2.98E-04 | | -0.10 | | 8.02E-01 | -0.06 | 9.01E-01 | |
| BICDL1 | 0.83 | | 5.70E-05 | 0.27 | 6.12E-01 | | 0.54 | 1.42E-01 | |  | | SNRPD3 | -0.52 | 1.97E-03 | | 0.01 | | 9.85E-01 | -0.03 | 9.65E-01 | |
| SLC43A2 | 0.83 | | 3.22E-05 | 0.10 | 8.81E-01 | | 0.12 | 8.56E-01 | |  | | TRNAU1AP | -0.52 | 5.65E-03 | | -0.22 | | 5.65E-01 | -0.21 | 6.35E-01 | |
| STARD7-AS1 | 0.83 | | 9.20E-03 | 0.04 | 9.75E-01 | | -0.03 | 9.77E-01 | |  | | COMT | -0.52 | 3.43E-04 | | 0.41 | | 7.21E-02 | 0.31 | 4.34E-01 | |
| TSPAN2 | 0.83 | | 1.91E-03 | -0.50 | 3.50E-01 | | -0.42 | 4.85E-01 | |  | | ZBTB8OS | -0.52 | 1.35E-03 | | 0.02 | | 9.76E-01 | -0.27 | 4.40E-01 | |
| TIPARP | 0.83 | | 4.39E-07 | 0.26 | 4.41E-01 | | 0.28 | 4.69E-01 | |  | | LPAR1 | -0.52 | 3.54E-04 | | 0.10 | | 8.50E-01 | -0.12 | 7.91E-01 | |
| CHD2 | 0.83 | | 5.65E-08 | -0.11 | 8.14E-01 | | -0.20 | 6.42E-01 | |  | | NARF | -0.53 | 3.16E-04 | | -0.03 | | 9.60E-01 | 0.10 | 8.49E-01 | |
| PXK | 0.82 | | 3.70E-05 | 0.00 | 9.98E-01 | | -0.06 | 9.30E-01 | |  | | TMEM120A | -0.53 | 8.60E-03 | | -0.09 | | 8.85E-01 | -0.38 | 3.26E-01 | |
| XRN1 | 0.82 | | 6.37E-07 | -0.13 | 8.15E-01 | | 0.42 | 4.12E-01 | |  | | P2RY2 | -0.53 | 1.36E-03 | | -0.09 | | 8.54E-01 | -0.15 | 7.78E-01 | |
| FOXN2 | 0.82 | | 2.28E-06 | 0.07 | 9.30E-01 | | 0.18 | 7.92E-01 | |  | | DDX3X | -0.53 | 1.94E-03 | | -0.22 | | 5.90E-01 | -0.33 | 4.12E-01 | |
| PTPRZ1 | 0.82 | | 1.00E-05 | -0.40 | 3.08E-01 | | 0.38 | 2.88E-01 | |  | | TM7SF3 | -0.53 | 1.33E-04 | | -0.21 | | 6.00E-01 | -0.26 | 4.13E-01 | |
| SRGAP2C | 0.82 | | 4.80E-04 | 0.08 | 9.29E-01 | | 0.08 | 9.20E-01 | |  | | ILVBL | -0.53 | 2.18E-03 | | -0.12 | | 8.15E-01 | -0.07 | 9.24E-01 | |
| RANBP9 | 0.82 | | 3.69E-03 | 0.54 | 2.58E-01 | | 0.53 | 2.63E-01 | |  | | ABHD2 | -0.53 | 5.93E-03 | | -0.15 | | 7.45E-01 | -0.06 | 9.26E-01 | |
| OSGIN2 | 0.82 | | 3.49E-07 | -0.04 | 9.59E-01 | | 0.03 | 9.64E-01 | |  | | TMEM109 | -0.53 | 6.18E-04 | | 0.01 | | 9.87E-01 | 0.14 | 7.67E-01 | |
| LINC00649 | 0.82 | | 7.07E-03 | 0.01 | 9.90E-01 | | 0.43 | 4.10E-01 | |  | | CLU | -0.53 | 6.91E-05 | | -0.34 | | 1.20E-01 | -0.36 | 2.47E-01 | |
| ZC3H12C | 0.82 | | 1.59E-03 | 0.18 | 8.05E-01 | | 0.13 | 8.69E-01 | |  | | APMAP | -0.53 | 6.83E-05 | | -0.04 | | 9.40E-01 | -0.13 | 7.62E-01 | |
| JAG1 | 0.81 | | 5.45E-06 | -0.39 | 2.38E-01 | | -0.19 | 6.74E-01 | |  | | C1GALT1C1 | -0.53 | 1.07E-03 | | 0.00 | | 9.96E-01 | 0.04 | 9.52E-01 | |
| HPS4 | 0.81 | | 3.34E-07 | 0.15 | 7.17E-01 | | 0.29 | 4.07E-01 | |  | | MED28 | -0.53 | 1.66E-03 | | -0.04 | | 9.44E-01 | -0.14 | 7.55E-01 | |
| SIAH1 | 0.81 | | 2.80E-03 | -0.14 | 8.55E-01 | | -0.30 | 6.60E-01 | |  | | E2F2 | -0.53 | 1.12E-03 | | -0.37 | | 1.56E-01 | -0.45 | 1.45E-01 | |
| ZNF16 | 0.81 | | 7.72E-04 | -0.16 | 8.20E-01 | | 0.01 | 9.92E-01 | |  | | LRP1 | -0.53 | 2.24E-03 | | -0.16 | | 7.06E-01 | 0.03 | 9.66E-01 | |
| TPRA1 | 0.81 | | 1.46E-09 | -0.06 | 9.29E-01 | | 0.11 | 8.29E-01 | |  | | PINK1 | -0.53 | 7.35E-03 | | -0.26 | | 4.70E-01 | -0.33 | 4.85E-01 | |
| TAMM41 | 0.81 | | 2.78E-06 | 0.15 | 7.64E-01 | | 0.24 | 5.85E-01 | |  | | NPRL2 | -0.53 | 4.43E-03 | | -0.03 | | 9.65E-01 | 0.01 | 9.87E-01 | |
| USP36 | 0.81 | | 1.64E-08 | 0.28 | 3.48E-01 | | 0.35 | 2.85E-01 | |  | | HSPB11 | -0.53 | 3.64E-03 | | 0.27 | | 4.22E-01 | 0.00 | 1.00E+00 | |
| LRIG2 | 0.81 | | 1.76E-03 | 0.03 | 9.71E-01 | | 0.09 | 9.02E-01 | |  | | NDUFAF6 | -0.54 | 1.18E-03 | | -0.18 | | 6.33E-01 | -0.46 | 1.10E-01 | |
| XPOT | 0.81 | | 4.64E-07 | -0.13 | 8.19E-01 | | -0.13 | 8.16E-01 | |  | | CDC42 | -0.54 | 7.41E-05 | | -0.10 | | 8.03E-01 | -0.21 | 5.92E-01 | |
| TSTD2 | 0.81 | | 5.08E-06 | -0.36 | 3.39E-01 | | -0.31 | 4.12E-01 | |  | | DSN1 | -0.54 | 2.80E-04 | | -0.29 | | 3.89E-01 | -0.35 | 2.12E-01 | |
| AMOTL2 | 0.81 | | 1.59E-06 | -0.14 | 7.78E-01 | | 0.01 | 9.85E-01 | |  | | ZNHIT1 | -0.54 | 1.92E-03 | | 0.17 | | 7.33E-01 | 0.03 | 9.62E-01 | |
| BRAP | 0.81 | | 2.82E-07 | -0.13 | 7.61E-01 | | -0.10 | 8.36E-01 | |  | | ATP1B3 | -0.54 | 2.91E-05 | | -0.25 | | 2.89E-01 | -0.37 | 1.38E-01 | |
| C19orf25 | 0.81 | | 1.74E-07 | 0.18 | 6.90E-01 | | 0.22 | 6.85E-01 | |  | | ZNRF1 | -0.54 | 8.91E-04 | | -0.14 | | 7.89E-01 | -0.31 | 4.28E-01 | |
| PXDC1 | 0.81 | | 5.23E-05 | -0.41 | 1.87E-01 | | -0.37 | 3.33E-01 | |  | | ASF1B | -0.54 | 1.86E-04 | | 0.01 | | 9.80E-01 | -0.02 | 9.74E-01 | |
| MLLT10 | 0.80 | | 4.29E-04 | -0.13 | 8.54E-01 | | -0.30 | 5.33E-01 | |  | | RNF130 | -0.54 | 5.66E-03 | | 0.11 | | 8.32E-01 | 0.02 | 9.67E-01 | |
| CACNB1 | 0.80 | | 5.93E-03 | 0.05 | 9.67E-01 | | -0.31 | 6.59E-01 | |  | | HDAC8 | -0.54 | 2.64E-03 | | -0.09 | | 8.65E-01 | -0.08 | 8.90E-01 | |
| FBXO11 | 0.80 | | 1.26E-05 | 0.20 | 6.99E-01 | | 0.26 | 6.87E-01 | |  | | PTK7 | -0.54 | 6.24E-04 | | -0.05 | | 9.33E-01 | -0.02 | 9.78E-01 | |
| RELA | 0.80 | | 4.57E-08 | 0.27 | 3.85E-01 | | 0.42 | 1.60E-01 | |  | | HYAL2 | -0.54 | 9.44E-04 | | -0.11 | | 8.19E-01 | 0.06 | 9.26E-01 | |
| ZNF558 | 0.80 | | 2.08E-06 | -0.02 | 9.81E-01 | | 0.05 | 9.31E-01 | |  | | SELENOW | -0.54 | 1.88E-03 | | 0.12 | | 8.11E-01 | 0.01 | 9.79E-01 | |
| TRAPPC6B | 0.80 | | 2.39E-05 | 0.04 | 9.60E-01 | | 0.10 | 8.87E-01 | |  | | RAB11A | -0.54 | 1.32E-04 | | -0.07 | | 8.82E-01 | -0.06 | 9.18E-01 | |
| ZBTB48 | 0.80 | | 6.76E-06 | 0.31 | 3.22E-01 | | 0.44 | 1.97E-01 | |  | | RPS24 | -0.54 | 4.30E-03 | | 0.18 | | 6.70E-01 | -0.10 | 8.41E-01 | |
| ZNF12 | 0.80 | | 5.11E-07 | -0.17 | 7.43E-01 | | -0.35 | 4.84E-01 | |  | | NUCB2 | -0.54 | 1.63E-03 | | 0.06 | | 9.27E-01 | -0.31 | 4.58E-01 | |
| NT5C3A | 0.80 | | 4.41E-09 | 0.14 | 7.17E-01 | | 0.16 | 7.93E-01 | |  | | FKBP4 | -0.54 | 3.83E-04 | | -0.03 | | 9.60E-01 | 0.03 | 9.47E-01 | |
| ZNF773 | 0.80 | | 9.34E-03 | -0.05 | 9.65E-01 | | -0.09 | 9.22E-01 | |  | | TMEM106C | -0.54 | 5.78E-05 | | -0.26 | | 2.62E-01 | -0.38 | 1.07E-01 | |
| ZNF525 | 0.80 | | 1.36E-03 | -0.16 | 7.91E-01 | | -0.06 | 9.37E-01 | |  | | LNPK | -0.55 | 4.28E-03 | | -0.19 | | 6.97E-01 | -0.38 | 3.83E-01 | |
| PRDM4 | 0.79 | | 2.39E-06 | 0.07 | 9.13E-01 | | 0.28 | 4.06E-01 | |  | | PLOD1 | -0.55 | 7.56E-04 | | 0.02 | | 9.76E-01 | 0.03 | 9.67E-01 | |
| EVC2 | 0.79 | | 1.59E-03 | 0.24 | 6.83E-01 | | 0.41 | 3.99E-01 | |  | | RABIF | -0.55 | 7.66E-04 | | -0.02 | | 9.73E-01 | -0.21 | 5.73E-01 | |
| NBN | 0.79 | | 3.20E-10 | -0.03 | 9.64E-01 | | 0.28 | 5.38E-01 | |  | | OCLN | -0.55 | 1.70E-03 | | -0.42 | | 1.15E-01 | -0.48 | 1.08E-01 | |
| TGDS | 0.79 | | 1.12E-07 | 0.25 | 4.40E-01 | | 0.20 | 7.04E-01 | |  | | ATP5MC2 | -0.55 | 9.54E-04 | | 0.02 | | 9.76E-01 | -0.29 | 4.26E-01 | |
| XIAP | 0.79 | | 1.88E-03 | 0.22 | 7.23E-01 | | 0.32 | 6.17E-01 | |  | | AKR7A2 | -0.55 | 5.22E-05 | | -0.03 | | 9.61E-01 | -0.10 | 8.62E-01 | |
| GRAMD2B | 0.79 | | 1.02E-07 | -0.54 | 6.44E-03 | | 0.04 | 9.45E-01 | |  | | PNKD | -0.55 | 1.20E-03 | | -0.05 | | 9.45E-01 | -0.15 | 7.77E-01 | |
| SLX4IP | 0.79 | | 5.46E-06 | -0.29 | 3.95E-01 | | -0.14 | 7.92E-01 | |  | | SUMO2 | -0.55 | 3.06E-03 | | 0.09 | | 8.52E-01 | 0.09 | 8.64E-01 | |
| ZZZ3 | 0.79 | | 1.33E-06 | -0.13 | 7.99E-01 | | -0.16 | 7.90E-01 | |  | | LIG1 | -0.55 | 6.18E-04 | | -0.26 | | 4.22E-01 | -0.12 | 8.34E-01 | |
| ZNF740 | 0.78 | | 9.24E-04 | 0.24 | 6.72E-01 | | 0.22 | 7.15E-01 | |  | | RBBP4 | -0.55 | 4.19E-05 | | -0.35 | | 1.05E-01 | -0.34 | 2.74E-01 | |
| RPP38 | 0.78 | | 1.49E-05 | 0.13 | 8.16E-01 | | 0.23 | 6.08E-01 | |  | | CCDC117 | -0.55 | 6.74E-03 | | -0.55 | | 1.79E-01 | -0.56 | 1.81E-01 | |
| FAM76B | 0.78 | | 7.27E-05 | 0.03 | 9.67E-01 | | 0.01 | 9.87E-01 | |  | | ESYT1 | -0.55 | 7.74E-05 | | 0.24 | | 3.54E-01 | 0.25 | 4.84E-01 | |
| MOCOS | 0.78 | | 8.03E-06 | -0.07 | 9.15E-01 | | 0.28 | 4.54E-01 | |  | | NDUFAF8 | -0.55 | 4.52E-04 | | 0.25 | | 5.61E-01 | -0.14 | 8.07E-01 | |
| ZNF678 | 0.78 | | 5.58E-03 | 0.00 | 9.99E-01 | | 0.11 | 8.98E-01 | |  | | TPCN1 | -0.56 | 2.61E-04 | | 0.44 | | 3.50E-02 | 0.34 | 2.23E-01 | |
| SLC25A30 | 0.78 | | 4.83E-04 | 0.04 | 9.68E-01 | | 0.26 | 6.62E-01 | |  | | SEC13 | -0.56 | 1.63E-04 | | 0.01 | | 9.80E-01 | -0.02 | 9.69E-01 | |
| ZNF350 | 0.78 | | 4.46E-04 | -0.03 | 9.73E-01 | | -0.19 | 7.44E-01 | |  | | ATG4A | -0.56 | 2.23E-03 | | 0.20 | | 6.09E-01 | 0.37 | 2.41E-01 | |
| ATXN7 | 0.78 | | 1.36E-05 | -0.19 | 6.79E-01 | | 0.09 | 8.83E-01 | |  | | SF3B5 | -0.56 | 3.47E-04 | | 0.23 | | 5.36E-01 | 0.00 | 9.99E-01 | |
| KRT17 | 0.78 | | 5.39E-09 | -0.36 | 1.06E-01 | | 0.20 | 7.02E-01 | |  | | SSR2 | -0.56 | 2.48E-05 | | 0.17 | | 5.49E-01 | 0.01 | 9.87E-01 | |
| ZNF48 | 0.78 | | 7.79E-03 | 0.02 | 9.87E-01 | | -0.26 | 7.14E-01 | |  | | TMEM179B | -0.56 | 8.93E-04 | | 0.20 | | 6.00E-01 | -0.12 | 8.21E-01 | |
| UVRAG | 0.78 | | 7.27E-06 | -0.08 | 8.99E-01 | | 0.11 | 8.54E-01 | |  | | PTPN4 | -0.56 | 4.37E-03 | | -0.18 | | 7.61E-01 | -0.18 | 7.26E-01 | |
| PMS2CL | 0.78 | | 1.37E-04 | 0.11 | 8.69E-01 | | -0.11 | 8.63E-01 | |  | | UNC119 | -0.56 | 4.83E-04 | | -0.05 | | 9.34E-01 | -0.20 | 6.49E-01 | |
| HINT3 | 0.78 | | 3.10E-04 | -0.69 | 2.89E-02 | | -0.61 | 1.45E-01 | |  | | NECTIN4 | -0.56 | 1.14E-03 | | -0.43 | | 4.99E-02 | -0.18 | 7.25E-01 | |
| AMMECR1L | 0.78 | | 2.92E-05 | -0.11 | 8.07E-01 | | -0.17 | 7.06E-01 | |  | | GLB1L2 | -0.56 | 2.12E-04 | | 0.12 | | 7.92E-01 | -0.17 | 7.15E-01 | |
| FAM13B | 0.78 | | 4.72E-05 | -0.23 | 6.05E-01 | | -0.37 | 4.77E-01 | |  | | LRATD1 | -0.56 | 1.07E-03 | | 0.11 | | 7.80E-01 | 0.17 | 6.49E-01 | |
| PARD6B | 0.78 | | 2.83E-04 | 0.16 | 7.77E-01 | | 0.37 | 4.96E-01 | |  | | VPS18 | -0.56 | 1.07E-03 | | -0.07 | | 9.24E-01 | 0.30 | 4.83E-01 | |
| ARL14EP | 0.78 | | 1.73E-05 | 0.00 | 9.98E-01 | | -0.24 | 5.76E-01 | |  | | UAP1L1 | -0.56 | 1.23E-03 | | -0.26 | | 4.72E-01 | -0.39 | 2.32E-01 | |
| AFDN | 0.77 | | 1.11E-03 | 0.38 | 3.18E-01 | | 0.44 | 2.56E-01 | |  | | AIP | -0.56 | 1.21E-03 | | 0.17 | | 6.82E-01 | 0.21 | 7.12E-01 | |
| OTUD4 | 0.77 | | 7.91E-06 | 0.00 | 9.98E-01 | | 0.36 | 3.27E-01 | |  | | TSPAN6 | -0.57 | 2.08E-04 | | -0.05 | | 9.43E-01 | -0.29 | 4.66E-01 | |
| TAB2 | 0.77 | | 8.12E-05 | -0.37 | 3.46E-01 | | -0.32 | 5.10E-01 | |  | | USP21 | -0.57 | 6.24E-04 | | -0.05 | | 9.37E-01 | -0.02 | 9.68E-01 | |
| NOP14-AS1 | 0.77 | | 5.96E-05 | 0.39 | 2.90E-01 | | 0.47 | 1.74E-01 | |  | | CLCN2 | -0.57 | 1.12E-03 | | 0.23 | | 5.20E-01 | 0.11 | 8.42E-01 | |
| MARK3 | 0.77 | | 1.58E-07 | -0.01 | 9.87E-01 | | 0.10 | 8.32E-01 | |  | | NCAPH | -0.57 | 5.48E-04 | | -0.05 | | 9.24E-01 | -0.04 | 9.46E-01 | |
| PAWR | 0.77 | | 9.06E-06 | -0.34 | 2.97E-01 | | -0.35 | 4.18E-01 | |  | | GTSE1 | -0.57 | 1.91E-03 | | -0.06 | | 9.22E-01 | 0.05 | 9.34E-01 | |
| NFKBIE | 0.77 | | 1.04E-05 | -0.24 | 5.62E-01 | | 0.22 | 6.51E-01 | |  | | XRCC2 | -0.57 | 5.39E-03 | | 0.00 | | 9.97E-01 | -0.49 | 1.31E-01 | |
| FEM1C | 0.76 | | 8.49E-05 | -0.20 | 6.95E-01 | | 0.12 | 8.44E-01 | |  | | FOPNL | -0.57 | 1.79E-04 | | -0.14 | | 7.69E-01 | -0.44 | 2.24E-01 | |
| JUN | 0.76 | | 1.13E-04 | -0.30 | 4.43E-01 | | -0.15 | 7.82E-01 | |  | | ACP6 | -0.57 | 1.02E-03 | | -0.19 | | 6.16E-01 | -0.30 | 3.24E-01 | |
| SPIRE1 | 0.76 | | 2.81E-03 | 0.41 | 2.83E-01 | | 0.26 | 6.33E-01 | |  | | PDXK | -0.57 | 3.82E-06 | | -0.41 | | 2.59E-02 | -0.35 | 2.92E-01 | |
| ZNF267 | 0.76 | | 1.97E-07 | -0.19 | 6.55E-01 | | 0.40 | 2.96E-01 | |  | | VCL | -0.57 | 2.71E-04 | | -0.41 | | 8.49E-02 | -0.45 | 1.04E-01 | |
| ANKRD12 | 0.76 | | 3.90E-04 | 0.14 | 8.14E-01 | | 0.01 | 9.88E-01 | |  | | PIAS2 | -0.57 | 1.16E-03 | | -0.23 | | 5.38E-01 | -0.37 | 4.12E-01 | |
| RFX5 | 0.76 | | 2.50E-06 | -0.29 | 3.78E-01 | | 0.27 | 4.86E-01 | |  | | TRMT112 | -0.57 | 1.96E-03 | | 0.13 | | 8.08E-01 | -0.11 | 8.34E-01 | |
| ZNF260 | 0.76 | | 4.13E-03 | -0.16 | 8.29E-01 | | -0.37 | 5.83E-01 | |  | | BCKDHB | -0.57 | 4.15E-03 | | -0.45 | | 1.47E-01 | -0.57 | 1.32E-01 | |
| TRAF5 | 0.76 | | 4.39E-03 | 0.06 | 9.60E-01 | | 0.08 | 9.31E-01 | |  | | TCEAL8 | -0.57 | 3.52E-03 | | 0.16 | | 7.25E-01 | 0.12 | 8.46E-01 | |
| DLC1 | 0.76 | | 2.26E-04 | 0.16 | 7.71E-01 | | 0.26 | 5.44E-01 | |  | | RPS16 | -0.57 | 3.09E-03 | | 0.30 | | 3.86E-01 | 0.22 | 6.45E-01 | |
| SHB | 0.75 | | 2.55E-04 | 0.39 | 3.14E-01 | | 0.49 | 2.58E-01 | |  | | RPA3 | -0.57 | 9.50E-04 | | 0.08 | | 8.88E-01 | -0.24 | 5.52E-01 | |
| C2orf42 | 0.75 | | 8.00E-05 | -0.03 | 9.71E-01 | | -0.25 | 5.85E-01 | |  | | CTNNB1 | -0.57 | 2.62E-04 | | -0.13 | | 7.67E-01 | -0.22 | 5.52E-01 | |
| USP43 | 0.75 | | 2.69E-06 | -0.40 | 1.70E-01 | | 0.22 | 6.32E-01 | |  | | AAR2 | -0.57 | 5.49E-04 | | -0.05 | | 9.23E-01 | 0.05 | 9.36E-01 | |
| ZNF613 | 0.75 | | 2.63E-03 | -0.36 | 5.71E-01 | | -0.16 | 8.13E-01 | |  | | SRSF2 | -0.57 | 8.31E-06 | | -0.23 | | 4.68E-01 | -0.25 | 6.04E-01 | |
| RFFL | 0.75 | | 1.79E-05 | 0.11 | 8.55E-01 | | 0.21 | 6.57E-01 | |  | | SFXN2 | -0.57 | 6.99E-04 | | -0.18 | | 6.40E-01 | -0.28 | 4.25E-01 | |
| EIF2S2 | 0.75 | | 1.21E-05 | 0.18 | 6.75E-01 | | 0.20 | 7.02E-01 | |  | | GRHPR | -0.57 | 3.90E-04 | | -0.24 | | 4.26E-01 | -0.21 | 6.51E-01 | |
| GATAD1 | 0.75 | | 2.12E-05 | 0.24 | 4.85E-01 | | 0.18 | 7.48E-01 | |  | | RPL31 | -0.57 | 3.19E-03 | | 0.18 | | 6.63E-01 | 0.04 | 9.49E-01 | |
| PIM1 | 0.75 | | 4.54E-06 | 0.26 | 3.98E-01 | | 0.41 | 1.64E-01 | |  | | WDR6 | -0.57 | 3.86E-04 | | -0.08 | | 8.83E-01 | 0.17 | 7.31E-01 | |
| POC5 | 0.75 | | 4.78E-07 | -0.05 | 9.40E-01 | | 0.05 | 9.30E-01 | |  | | UBA52 | -0.57 | 2.91E-03 | | 0.23 | | 6.06E-01 | 0.23 | 6.42E-01 | |
| ZNF506 | 0.75 | | 4.44E-03 | -0.32 | 5.74E-01 | | -0.20 | 7.70E-01 | |  | | TACSTD2 | -0.58 | 1.02E-04 | | 0.13 | | 7.43E-01 | 0.36 | 2.75E-01 | |
| PNPLA8 | 0.75 | | 6.27E-06 | 0.05 | 9.47E-01 | | 0.18 | 7.86E-01 | |  | | RPS25 | -0.58 | 5.26E-03 | | 0.25 | | 4.95E-01 | 0.10 | 8.50E-01 | |
| THAP6 | 0.75 | | 2.14E-04 | 0.07 | 9.29E-01 | | 0.02 | 9.83E-01 | |  | | PIM2 | -0.58 | 2.59E-03 | | 0.17 | | 6.86E-01 | 0.20 | 6.51E-01 | |
| THAP1 | 0.75 | | 1.92E-05 | 0.09 | 8.98E-01 | | 0.08 | 8.97E-01 | |  | | RAB4A | -0.58 | 1.08E-04 | | -0.23 | | 5.08E-01 | -0.44 | 1.47E-01 | |
| PLCXD2 | 0.75 | | 6.51E-03 | -0.13 | 8.48E-01 | | -0.07 | 9.23E-01 | |  | | FZD1 | -0.58 | 3.44E-03 | | -0.69 | | 2.78E-03 | -0.41 | 2.94E-01 | |
| CDC37L1 | 0.74 | | 3.90E-04 | -0.34 | 4.85E-01 | | -0.49 | 2.50E-01 | |  | | PTOV1 | -0.58 | 2.08E-03 | | -0.05 | | 9.47E-01 | 0.01 | 9.91E-01 | |
| RAB9A | 0.74 | | 2.87E-05 | -0.08 | 8.85E-01 | | -0.22 | 5.88E-01 | |  | | THOC3 | -0.58 | 8.05E-04 | | -0.09 | | 8.55E-01 | -0.19 | 7.39E-01 | |
| RAB27B | 0.74 | | 9.56E-05 | 0.17 | 7.78E-01 | | 0.52 | 3.14E-01 | |  | | NDUFA8 | -0.58 | 1.21E-05 | | -0.20 | | 4.63E-01 | -0.35 | 2.02E-01 | |
| SOWAHC | 0.74 | | 5.08E-05 | -0.05 | 9.39E-01 | | 0.02 | 9.78E-01 | |  | | TCIRG1 | -0.58 | 2.65E-04 | | 0.10 | | 8.72E-01 | 0.31 | 4.56E-01 | |
| PARP6 | 0.74 | | 3.69E-06 | -0.01 | 9.80E-01 | | 0.15 | 7.07E-01 | |  | | VAT1 | -0.58 | 5.34E-05 | | 0.09 | | 8.50E-01 | 0.05 | 9.45E-01 | |
| FMNL2 | 0.74 | | 1.34E-04 | 0.57 | 2.00E-02 | | 0.54 | 1.29E-01 | |  | | SMIM3 | -0.58 | 2.11E-03 | | 0.41 | | 9.99E-02 | 0.33 | 3.10E-01 | |
| P2RX4 | 0.74 | | 6.36E-03 | -0.24 | 7.27E-01 | | 0.19 | 8.02E-01 | |  | | VDAC3 | -0.58 | 5.17E-06 | | -0.26 | | 3.87E-01 | -0.29 | 2.90E-01 | |
| MAFG | 0.74 | | 2.38E-07 | 0.21 | 6.00E-01 | | 0.32 | 3.23E-01 | |  | | AGO1 | -0.58 | 1.59E-03 | | -0.04 | | 9.52E-01 | -0.22 | 6.09E-01 | |
| GGNBP2 | 0.74 | | 4.12E-08 | -0.02 | 9.68E-01 | | -0.08 | 8.69E-01 | |  | | LRRC45 | -0.59 | 5.37E-04 | | 0.00 | | 9.96E-01 | 0.15 | 8.16E-01 | |
| APPBP2 | 0.74 | | 5.37E-05 | -0.11 | 8.53E-01 | | 0.02 | 9.82E-01 | |  | | OAT | -0.59 | 4.59E-06 | | 0.09 | | 8.41E-01 | 0.01 | 9.85E-01 | |
| SMOX | 0.74 | | 4.35E-07 | 0.51 | 1.84E-02 | | 0.15 | 7.88E-01 | |  | | TMX4 | -0.59 | 1.25E-03 | | 0.03 | | 9.67E-01 | -0.14 | 7.52E-01 | |
| ANKS3 | 0.73 | | 6.51E-05 | 0.20 | 6.77E-01 | | 0.26 | 6.64E-01 | |  | | DGAT1 | -0.59 | 2.66E-03 | | 0.25 | | 5.40E-01 | 0.21 | 6.88E-01 | |
| ZNF195 | 0.73 | | 1.02E-06 | -0.03 | 9.63E-01 | | 0.09 | 8.61E-01 | |  | | AHSA2P | -0.59 | 4.37E-03 | | 0.32 | | 3.48E-01 | 0.03 | 9.66E-01 | |
| NABP1 | 0.73 | | 2.85E-06 | 0.25 | 6.46E-01 | | 0.36 | 5.25E-01 | |  | | EMC9 | -0.59 | 5.27E-04 | | 0.01 | | 9.93E-01 | -0.27 | 5.38E-01 | |
| C10orf88 | 0.73 | | 8.76E-04 | -0.01 | 9.92E-01 | | 0.21 | 7.52E-01 | |  | | BCAS4 | -0.59 | 1.65E-03 | | -0.07 | | 9.06E-01 | -0.16 | 7.64E-01 | |
| RUNX2 | 0.73 | | 4.05E-03 | 0.23 | 6.81E-01 | | 0.05 | 9.50E-01 | |  | | SLC26A6 | -0.59 | 1.49E-03 | | 0.15 | | 7.28E-01 | 0.18 | 7.30E-01 | |
| INTS6 | 0.73 | | 1.04E-05 | -0.19 | 7.15E-01 | | -0.14 | 8.14E-01 | |  | | SEMA4D | -0.59 | 8.22E-03 | | 0.35 | | 2.84E-01 | 0.34 | 4.14E-01 | |
| TAF1A | 0.73 | | 1.76E-05 | 0.52 | 6.16E-02 | | 0.43 | 3.36E-01 | |  | | TMEM97 | -0.59 | 4.38E-04 | | -0.10 | | 8.20E-01 | -0.10 | 8.32E-01 | |
| UBA6 | 0.73 | | 7.75E-05 | 0.01 | 9.90E-01 | | 0.54 | 1.89E-01 | |  | | FAM20C | -0.59 | 8.96E-03 | | 0.16 | | 7.77E-01 | -0.03 | 9.67E-01 | |
| RSRC2 | 0.73 | | 6.98E-07 | -0.02 | 9.74E-01 | | 0.02 | 9.76E-01 | |  | | UXS1 | -0.60 | 2.89E-04 | | 0.06 | | 9.04E-01 | -0.10 | 8.17E-01 | |
| VAMP4 | 0.73 | | 1.90E-03 | 0.41 | 3.23E-01 | | 0.32 | 6.20E-01 | |  | | PRMT7 | -0.60 | 1.23E-04 | | 0.02 | | 9.74E-01 | 0.02 | 9.71E-01 | |
| EAF1 | 0.73 | | 5.11E-04 | 0.32 | 4.04E-01 | | 0.43 | 3.26E-01 | |  | | C12orf49 | -0.60 | 6.95E-05 | | -0.15 | | 6.83E-01 | -0.18 | 6.62E-01 | |
| AVL9 | 0.73 | | 8.43E-05 | 0.20 | 6.44E-01 | | 0.29 | 4.59E-01 | |  | | SNRNP25 | -0.60 | 6.29E-05 | | -0.04 | | 9.52E-01 | -0.09 | 8.73E-01 | |
| SAMD9 | 0.73 | | 3.11E-08 | -0.26 | 3.69E-01 | | 0.51 | 1.83E-01 | |  | | MT-CYB | -0.60 | 1.18E-03 | | 0.14 | | 7.66E-01 | -0.25 | 4.96E-01 | |
| RC3H2 | 0.73 | | 1.74E-07 | -0.03 | 9.59E-01 | | 0.15 | 7.26E-01 | |  | | KCNJ15 | -0.60 | 4.51E-03 | | -0.19 | | 7.45E-01 | -0.03 | 9.63E-01 | |
| ZNF215 | 0.73 | | 1.22E-04 | 0.40 | 1.95E-01 | | 0.37 | 3.81E-01 | |  | | CACYBP | -0.60 | 1.79E-04 | | 0.11 | | 7.72E-01 | -0.13 | 7.91E-01 | |
| FBLIM1 | 0.73 | | 1.70E-05 | 0.38 | 1.88E-01 | | 0.48 | 2.07E-01 | |  | | R3HCC1 | -0.60 | 2.43E-04 | | -0.24 | | 4.88E-01 | -0.35 | 3.66E-01 | |
| SECISBP2 | 0.72 | | 4.85E-06 | 0.27 | 3.88E-01 | | 0.27 | 4.87E-01 | |  | | DNAJA1 | -0.60 | 1.50E-05 | | -0.17 | | 6.95E-01 | 0.35 | 2.50E-01 | |
| NHS | 0.72 | | 4.29E-04 | -0.27 | 4.83E-01 | | -0.38 | 2.85E-01 | |  | | ENDOD1 | -0.60 | 8.43E-05 | | -0.13 | | 7.52E-01 | -0.10 | 8.36E-01 | |
| TMEM40 | 0.72 | | 9.70E-06 | 0.27 | 4.45E-01 | | 0.39 | 3.04E-01 | |  | | MSH2 | -0.60 | 2.24E-05 | | -0.28 | | 3.59E-01 | -0.44 | 1.87E-01 | |
| MSL2 | 0.72 | | 4.83E-06 | -0.03 | 9.59E-01 | | -0.15 | 7.70E-01 | |  | | LTA4H | -0.60 | 2.18E-04 | | 0.12 | | 7.59E-01 | 0.02 | 9.68E-01 | |
| TRAF4 | 0.72 | | 1.74E-04 | 0.06 | 9.31E-01 | | 0.10 | 8.98E-01 | |  | | TAF6L | -0.60 | 9.25E-04 | | 0.08 | | 8.85E-01 | 0.23 | 6.38E-01 | |
| TMEM268 | 0.72 | | 4.68E-05 | -0.25 | 4.99E-01 | | -0.28 | 4.62E-01 | |  | | KHK | -0.60 | 5.15E-03 | | -0.21 | | 6.00E-01 | -0.16 | 7.61E-01 | |
| TRMT10B | 0.72 | | 6.29E-03 | -0.04 | 9.67E-01 | | -0.06 | 9.38E-01 | |  | | SDF2L1 | -0.61 | 2.06E-04 | | 0.31 | | 3.72E-01 | 0.53 | 1.23E-01 | |
| GTF2IRD1 | 0.72 | | 1.46E-04 | -0.03 | 9.62E-01 | | 0.30 | 5.06E-01 | |  | | HS2ST1 | -0.61 | 5.14E-03 | | -0.23 | | 7.05E-01 | -0.37 | 5.32E-01 | |
| ESCO1 | 0.72 | | 2.30E-03 | 0.02 | 9.85E-01 | | -0.09 | 9.25E-01 | |  | | ACSF2 | -0.61 | 9.53E-03 | | -0.48 | | 1.43E-01 | -0.33 | 4.81E-01 | |
| AMPD3 | 0.72 | | 2.65E-04 | 0.35 | 2.74E-01 | | 0.49 | 1.13E-01 | |  | | RNASEH2C | -0.61 | 1.44E-04 | | 0.12 | | 8.12E-01 | 0.19 | 7.44E-01 | |
| C1orf109 | 0.72 | | 6.09E-07 | 0.13 | 7.52E-01 | | 0.15 | 7.40E-01 | |  | | ANLN | -0.61 | 1.05E-04 | | -0.40 | | 1.61E-01 | -0.46 | 1.55E-01 | |
| ZNF544 | 0.72 | | 7.72E-05 | -0.07 | 9.10E-01 | | -0.04 | 9.49E-01 | |  | | PSMD12 | -0.61 | 9.61E-06 | | 0.01 | | 9.92E-01 | -0.11 | 8.38E-01 | |
| HSBP1L1 | 0.72 | | 1.12E-05 | -0.21 | 5.59E-01 | | -0.15 | 7.45E-01 | |  | | NDUFA1 | -0.61 | 1.52E-03 | | 0.04 | | 9.55E-01 | -0.32 | 3.11E-01 | |
| PRR3 | 0.72 | | 9.49E-04 | -0.17 | 7.79E-01 | | 0.16 | 7.56E-01 | |  | | SAFB | -0.61 | 1.88E-04 | | 0.04 | | 9.54E-01 | 0.08 | 8.75E-01 | |
| FAM117B | 0.72 | | 6.18E-04 | 0.00 | 9.98E-01 | | 0.50 | 1.43E-01 | |  | | TUBB2A | -0.61 | 2.44E-03 | | -0.24 | | 5.49E-01 | -0.14 | 7.91E-01 | |
| ZSCAN29 | 0.72 | | 1.21E-04 | -0.02 | 9.81E-01 | | 0.11 | 8.42E-01 | |  | | SGSM3 | -0.61 | 1.65E-04 | | -0.25 | | 4.93E-01 | -0.10 | 8.73E-01 | |
| DET1 | 0.72 | | 6.07E-03 | -0.07 | 9.40E-01 | | -0.13 | 8.64E-01 | |  | | ATP6V0A1 | -0.61 | 2.18E-04 | | -0.15 | | 7.11E-01 | -0.14 | 7.39E-01 | |
| DRAM1 | 0.72 | | 2.32E-06 | -0.42 | 6.16E-02 | | 0.21 | 6.07E-01 | |  | | HILPDA | -0.61 | 7.62E-03 | | -0.01 | | 9.89E-01 | -0.23 | 6.06E-01 | |
| ZNF343 | 0.72 | | 2.43E-04 | -0.06 | 9.30E-01 | | 0.00 | 9.95E-01 | |  | | PLA2G12A | -0.61 | 6.78E-04 | | -0.16 | | 7.03E-01 | -0.35 | 2.77E-01 | |
| NOC3L | 0.71 | | 2.10E-06 | 0.23 | 4.64E-01 | | 0.04 | 9.61E-01 | |  | | POMGNT1 | -0.61 | 3.74E-04 | | -0.19 | | 6.12E-01 | -0.31 | 3.94E-01 | |
| PTCD2 | 0.71 | | 2.87E-05 | -0.04 | 9.48E-01 | | -0.17 | 7.52E-01 | |  | | MALL | -0.61 | 3.69E-03 | | -0.24 | | 5.66E-01 | -0.27 | 5.12E-01 | |
| AP4E1 | 0.71 | | 1.17E-03 | -0.18 | 7.60E-01 | | -0.14 | 8.48E-01 | |  | | NLRP2 | -0.61 | 2.45E-04 | | -0.14 | | 7.22E-01 | -0.12 | 7.98E-01 | |
| LNX2 | 0.71 | | 1.75E-04 | -0.28 | 5.30E-01 | | -0.25 | 6.07E-01 | |  | | PDSS1 | -0.61 | 1.06E-03 | | -0.09 | | 8.85E-01 | -0.21 | 5.90E-01 | |
| ZNF462 | 0.71 | | 1.13E-04 | 0.15 | 7.98E-01 | | 0.15 | 7.71E-01 | |  | | TCEAL9 | -0.62 | 8.60E-03 | | 0.17 | | 7.64E-01 | 0.11 | 8.80E-01 | |
| ALOXE3 | 0.71 | | 2.57E-03 | 0.55 | 5.19E-02 | | 0.21 | 7.22E-01 | |  | | SYNJ2BP | -0.62 | 7.31E-03 | | 0.05 | | 9.47E-01 | -0.27 | 6.51E-01 | |
| LATS1 | 0.71 | | 3.06E-04 | -0.03 | 9.71E-01 | | -0.16 | 8.25E-01 | |  | | PRDX1 | -0.62 | 2.42E-04 | | -0.02 | | 9.71E-01 | -0.09 | 8.44E-01 | |
| SLC4A7 | 0.71 | | 1.04E-03 | 0.27 | 5.82E-01 | | 0.46 | 2.84E-01 | |  | | MT-RNR1 | -0.62 | 6.51E-03 | | 0.33 | | 3.29E-01 | 0.09 | 8.65E-01 | |
| PVR | 0.71 | | 1.16E-05 | 0.32 | 1.93E-01 | | 0.44 | 1.22E-01 | |  | | COPRS | -0.62 | 2.91E-05 | | 0.00 | | 9.95E-01 | -0.24 | 4.74E-01 | |
| CDC42EP4 | 0.71 | | 1.33E-04 | 0.10 | 8.68E-01 | | 0.42 | 2.56E-01 | |  | | MPST | -0.62 | 3.64E-03 | | -0.01 | | 9.88E-01 | -0.11 | 8.64E-01 | |
| ZNF614 | 0.71 | | 5.06E-03 | -0.05 | 9.59E-01 | | 0.32 | 5.41E-01 | |  | | S100A10 | -0.62 | 8.28E-04 | | -0.03 | | 9.62E-01 | -0.08 | 8.94E-01 | |
| CNST | 0.71 | | 2.19E-06 | 0.17 | 6.37E-01 | | 0.10 | 8.35E-01 | |  | | HELLS | -0.62 | 8.96E-06 | | -0.13 | | 7.61E-01 | -0.34 | 4.03E-01 | |
| SCAF8 | 0.71 | | 2.01E-04 | -0.02 | 9.74E-01 | | -0.12 | 8.51E-01 | |  | | CALM2 | -0.62 | 4.55E-06 | | 0.08 | | 8.37E-01 | -0.14 | 7.95E-01 | |
| MTHFD1L | 0.71 | | 1.70E-05 | 0.05 | 9.29E-01 | | 0.04 | 9.36E-01 | |  | | FOXRED2 | -0.62 | 1.35E-04 | | -0.14 | | 7.82E-01 | -0.14 | 7.68E-01 | |
| FOXO3 | 0.71 | | 1.35E-05 | -0.03 | 9.70E-01 | | 0.00 | 9.96E-01 | |  | | RNF26 | -0.62 | 6.63E-06 | | -0.18 | | 6.38E-01 | -0.13 | 7.98E-01 | |
| LRCH1 | 0.70 | | 1.33E-04 | 0.25 | 5.65E-01 | | 0.48 | 1.28E-01 | |  | | RPS27 | -0.62 | 5.93E-03 | | 0.31 | | 3.95E-01 | 0.20 | 6.77E-01 | |
| RSPRY1 | 0.70 | | 3.35E-07 | 0.11 | 8.02E-01 | | 0.40 | 1.45E-01 | |  | | SNX21 | -0.62 | 5.64E-03 | | -0.27 | | 5.95E-01 | -0.34 | 5.09E-01 | |
| MCPH1 | 0.70 | | 3.92E-06 | 0.22 | 5.70E-01 | | 0.18 | 7.04E-01 | |  | | DOLK | -0.62 | 9.28E-06 | | 0.03 | | 9.58E-01 | 0.03 | 9.58E-01 | |
| ZNF561 | 0.70 | | 1.34E-03 | 0.25 | 5.45E-01 | | 0.09 | 8.94E-01 | |  | | ACOX1 | -0.62 | 2.13E-04 | | -0.30 | | 3.30E-01 | -0.45 | 1.06E-01 | |
| APBB2 | 0.70 | | 2.42E-04 | 0.01 | 9.86E-01 | | 0.25 | 5.72E-01 | |  | | RPL21 | -0.63 | 5.38E-04 | | 0.12 | | 7.85E-01 | -0.09 | 8.60E-01 | |
| RWDD2B | 0.70 | | 4.45E-05 | 0.22 | 5.98E-01 | | 0.22 | 6.48E-01 | |  | | SLC6A11 | -0.63 | 2.37E-04 | | -0.18 | | 6.35E-01 | -0.19 | 6.43E-01 | |
| VAV2 | 0.70 | | 2.12E-06 | 0.07 | 8.97E-01 | | 0.31 | 3.86E-01 | |  | | FAM102A | -0.63 | 7.28E-05 | | -0.30 | | 2.95E-01 | -0.37 | 3.37E-01 | |
| PCED1B | 0.70 | | 2.10E-03 | 0.01 | 9.92E-01 | | 0.15 | 8.19E-01 | |  | | CENPV | -0.63 | 3.89E-03 | | 0.38 | | 1.80E-01 | -0.01 | 9.87E-01 | |
| KDM3A | 0.70 | | 3.19E-04 | -0.31 | 3.55E-01 | | -0.26 | 5.74E-01 | |  | | CYP27C1 | -0.63 | 3.04E-03 | | -0.21 | | 6.35E-01 | -0.45 | 1.72E-01 | |
| SH3BP4 | 0.70 | | 1.77E-04 | -0.03 | 9.59E-01 | | 0.00 | 9.96E-01 | |  | | RNPEP | -0.63 | 4.86E-07 | | -0.25 | | 2.95E-01 | -0.25 | 4.59E-01 | |
| CD47 | 0.70 | | 1.30E-07 | -0.44 | 1.82E-01 | | 0.42 | 2.68E-01 | |  | | COX6B1 | -0.63 | 3.37E-03 | | 0.15 | | 7.97E-01 | 0.09 | 9.01E-01 | |
| FAM200A | 0.70 | | 3.09E-03 | -0.01 | 9.94E-01 | | 0.01 | 9.93E-01 | |  | | CPNE2 | -0.63 | 1.81E-05 | | 0.12 | | 7.72E-01 | -0.14 | 7.63E-01 | |
| VEZF1 | 0.70 | | 5.43E-06 | -0.10 | 8.63E-01 | | -0.06 | 9.25E-01 | |  | | MAEA | -0.63 | 1.01E-04 | | -0.23 | | 4.50E-01 | -0.09 | 8.78E-01 | |
| TOP3A | 0.70 | | 1.33E-05 | 0.05 | 9.33E-01 | | 0.21 | 5.85E-01 | |  | | LYPD3 | -0.63 | 1.15E-04 | | 0.27 | | 3.39E-01 | 0.46 | 1.10E-01 | |
| ZHX2 | 0.70 | | 3.35E-03 | -0.05 | 9.53E-01 | | 0.03 | 9.69E-01 | |  | | SUN2 | -0.63 | 3.32E-05 | | -0.21 | | 5.70E-01 | -0.18 | 6.98E-01 | |
| CDV3 | 0.70 | | 3.57E-05 | -0.08 | 8.89E-01 | | -0.01 | 9.94E-01 | |  | | LRRC20 | -0.63 | 1.38E-03 | | 0.09 | | 8.80E-01 | -0.24 | 5.98E-01 | |
| MOB3C | 0.70 | | 1.28E-05 | -0.02 | 9.79E-01 | | 0.48 | 1.20E-01 | |  | | TTC38 | -0.63 | 6.07E-04 | | -0.27 | | 4.52E-01 | -0.03 | 9.65E-01 | |
| CAB39 | 0.70 | | 1.32E-06 | 0.07 | 9.20E-01 | | 0.43 | 2.14E-01 | |  | | FAM168A | -0.64 | 5.47E-03 | | -0.27 | | 5.20E-01 | -0.26 | 5.78E-01 | |
| ADA | 0.69 | | 6.44E-04 | -0.13 | 8.44E-01 | | 0.10 | 8.69E-01 | |  | | BBS9 | -0.64 | 7.76E-03 | | -0.43 | | 2.91E-01 | -0.53 | 1.25E-01 | |
| TJAP1 | 0.69 | | 1.18E-04 | 0.18 | 6.73E-01 | | 0.27 | 5.25E-01 | |  | | PTPRF | -0.64 | 9.60E-05 | | 0.35 | | 1.45E-01 | 0.41 | 1.85E-01 | |
| RCHY1 | 0.69 | | 1.34E-06 | -0.12 | 8.04E-01 | | -0.19 | 7.06E-01 | |  | | ENGASE | -0.64 | 8.67E-04 | | 0.05 | | 9.42E-01 | 0.01 | 9.84E-01 | |
| UBAP1 | 0.69 | | 9.70E-06 | -0.20 | 5.53E-01 | | -0.21 | 5.85E-01 | |  | | ERG28 | -0.64 | 1.75E-04 | | -0.12 | | 7.86E-01 | -0.08 | 8.68E-01 | |
| PIGBOS1 | 0.69 | | 2.16E-03 | 0.14 | 8.17E-01 | | 0.10 | 8.81E-01 | |  | | SPAG5 | -0.64 | 8.39E-05 | | -0.40 | | 1.16E-01 | -0.39 | 2.00E-01 | |
| ZKSCAN5 | 0.69 | | 2.47E-05 | -0.02 | 9.75E-01 | | 0.06 | 9.07E-01 | |  | | FAM114A1 | -0.64 | 5.18E-03 | | -0.23 | | 6.11E-01 | -0.30 | 4.85E-01 | |
| TAF1D | 0.69 | | 2.34E-05 | 0.27 | 3.94E-01 | | 0.21 | 7.16E-01 | |  | | ALDH1B1 | -0.64 | 8.44E-05 | | -0.17 | | 7.03E-01 | -0.18 | 6.81E-01 | |
| TYW5 | 0.69 | | 2.88E-04 | 0.22 | 6.10E-01 | | 0.17 | 7.97E-01 | |  | | RANBP6 | -0.64 | 1.26E-03 | | -0.17 | | 7.29E-01 | -0.10 | 8.90E-01 | |
| SYN1 | 0.69 | | 6.29E-03 | 0.56 | 1.32E-01 | | 0.43 | 3.35E-01 | |  | | RPL23A | -0.64 | 9.31E-04 | | 0.17 | | 6.74E-01 | 0.12 | 8.28E-01 | |
| MAP1LC3B | 0.69 | | 3.63E-05 | 0.10 | 7.91E-01 | | 0.03 | 9.60E-01 | |  | | CRELD2 | -0.65 | 1.62E-05 | | -0.01 | | 9.83E-01 | 0.10 | 8.63E-01 | |
| SNHG32 | 0.69 | | 5.56E-04 | 0.05 | 9.48E-01 | | -0.12 | 8.35E-01 | |  | | RPL34 | -0.65 | 1.43E-03 | | 0.30 | | 3.46E-01 | 0.24 | 5.92E-01 | |
| INO80 | 0.69 | | 7.64E-05 | 0.11 | 8.33E-01 | | 0.24 | 5.61E-01 | |  | | SQLE | -0.65 | 2.98E-06 | | -0.57 | | 3.57E-03 | -0.37 | 2.39E-01 | |
| RPS6KA5 | 0.69 | | 5.41E-05 | -0.14 | 7.81E-01 | | -0.14 | 8.11E-01 | |  | | TICRR | -0.65 | 7.07E-05 | | -0.16 | | 6.95E-01 | -0.22 | 5.70E-01 | |
| TRIM35 | 0.69 | | 8.72E-05 | 0.21 | 5.66E-01 | | 0.32 | 4.12E-01 | |  | | PCDHGC3 | -0.65 | 2.42E-04 | | 0.36 | | 1.98E-01 | 0.45 | 1.01E-01 | |
| RNF216P1 | 0.69 | | 4.52E-04 | 0.26 | 4.98E-01 | | 0.34 | 3.39E-01 | |  | | ALDH1A3 | -0.65 | 5.74E-05 | | 0.20 | | 5.96E-01 | 0.07 | 8.99E-01 | |
| CRAMP1 | 0.69 | | 2.36E-03 | 0.31 | 5.12E-01 | | 0.32 | 4.81E-01 | |  | | ANAPC15 | -0.65 | 9.65E-04 | | -0.30 | | 3.60E-01 | -0.54 | 1.10E-01 | |
| NEDD4 | 0.68 | | 1.10E-05 | -0.32 | 3.15E-01 | | -0.25 | 5.65E-01 | |  | | LXN | -0.65 | 6.98E-03 | | 0.05 | | 9.43E-01 | 0.06 | 9.38E-01 | |
| KCTD11 | 0.68 | | 1.86E-04 | -0.43 | 1.62E-01 | | -0.45 | 2.02E-01 | |  | | CCHCR1 | -0.65 | 2.80E-03 | | -0.22 | | 6.21E-01 | -0.10 | 8.90E-01 | |
| DYRK1A | 0.68 | | 5.54E-03 | 0.29 | 5.81E-01 | | -0.01 | 9.91E-01 | |  | | TP53I3 | -0.65 | 2.75E-03 | | 0.12 | | 8.24E-01 | -0.11 | 8.57E-01 | |
| MYCBP2 | 0.68 | | 3.34E-06 | 0.01 | 9.79E-01 | | 0.30 | 3.43E-01 | |  | | SEC61G | -0.65 | 9.50E-04 | | 0.40 | | 1.90E-01 | 0.08 | 9.13E-01 | |
| PRKAB1 | 0.68 | | 4.45E-05 | 0.12 | 7.88E-01 | | -0.05 | 9.36E-01 | |  | | MFSD5 | -0.66 | 8.30E-06 | | -0.16 | | 6.94E-01 | -0.07 | 9.12E-01 | |
| ARHGAP21 | 0.68 | | 1.34E-03 | -0.22 | 5.34E-01 | | -0.24 | 6.30E-01 | |  | | FBLN1 | -0.66 | 5.67E-06 | | -0.55 | | 1.16E-03 | -0.43 | 1.85E-01 | |
| ATF1 | 0.68 | | 2.08E-04 | 0.06 | 9.42E-01 | | 0.06 | 9.37E-01 | |  | | BRD3OS | -0.66 | 5.38E-05 | | -0.14 | | 7.40E-01 | -0.21 | 6.14E-01 | |
| IFRD1 | 0.68 | | 3.83E-05 | -0.18 | 7.65E-01 | | -0.19 | 6.51E-01 | |  | | RPL24 | -0.66 | 7.62E-04 | | 0.17 | | 7.06E-01 | -0.03 | 9.65E-01 | |
| EDN1 | 0.68 | | 1.27E-03 | -0.79 | 7.38E-03 | | -0.33 | 5.11E-01 | |  | | ERMP1 | -0.66 | 1.84E-05 | | -0.18 | | 6.87E-01 | -0.32 | 4.25E-01 | |
| FAM193A | 0.68 | | 2.72E-03 | -0.06 | 9.40E-01 | | 0.04 | 9.56E-01 | |  | | TPRG1L | -0.66 | 2.04E-03 | | -0.08 | | 9.08E-01 | -0.10 | 8.68E-01 | |
| COG3 | 0.68 | | 5.35E-05 | -0.04 | 9.62E-01 | | 0.12 | 8.12E-01 | |  | | EEF1A2 | -0.66 | 1.57E-04 | | -0.20 | | 6.69E-01 | -0.41 | 3.25E-01 | |
| LINC00909 | 0.68 | | 6.07E-03 | 0.09 | 9.22E-01 | | -0.12 | 8.69E-01 | |  | | GET1 | -0.66 | 1.62E-04 | | 0.18 | | 6.67E-01 | -0.15 | 7.50E-01 | |
| ZFP69 | 0.68 | | 2.12E-03 | -0.05 | 9.54E-01 | | -0.15 | 8.11E-01 | |  | | SLC19A1 | -0.67 | 6.52E-06 | | 0.21 | | 5.48E-01 | 0.09 | 8.97E-01 | |
| ARRDC2 | 0.68 | | 2.08E-04 | 0.13 | 8.24E-01 | | 0.32 | 4.61E-01 | |  | | CENPI | -0.67 | 4.09E-04 | | -0.27 | | 4.51E-01 | -0.29 | 4.96E-01 | |
| MCL1 | 0.68 | | 5.12E-06 | 0.14 | 7.66E-01 | | 0.25 | 6.02E-01 | |  | | CACNB3 | -0.67 | 7.43E-05 | | -0.16 | | 6.93E-01 | -0.26 | 5.43E-01 | |
| FKBP5 | 0.68 | | 5.24E-03 | -0.39 | 4.40E-01 | | 0.04 | 9.58E-01 | |  | | TYMS | -0.67 | 8.23E-05 | | -0.35 | | 2.68E-01 | -0.43 | 1.72E-01 | |
| PDCD11 | 0.67 | | 2.11E-06 | 0.29 | 2.09E-01 | | 0.38 | 1.28E-01 | |  | | JMJD6 | -0.67 | 1.43E-05 | | 0.21 | | 4.57E-01 | 0.21 | 5.67E-01 | |
| RHBDD1 | 0.67 | | 1.55E-04 | -0.11 | 8.37E-01 | | -0.04 | 9.46E-01 | |  | | LSM3 | -0.67 | 4.69E-03 | | 0.27 | | 5.22E-01 | 0.05 | 9.45E-01 | |
| CCDC82 | 0.67 | | 2.91E-05 | 0.04 | 9.54E-01 | | 0.32 | 5.58E-01 | |  | | INCENP | -0.67 | 2.13E-03 | | 0.13 | | 8.54E-01 | 0.19 | 7.32E-01 | |
| TRIM68 | 0.67 | | 3.07E-05 | -0.18 | 6.98E-01 | | -0.07 | 9.01E-01 | |  | | SLC25A44 | -0.67 | 1.01E-05 | | -0.20 | | 5.64E-01 | -0.19 | 6.31E-01 | |
| FBXO30 | 0.67 | | 8.76E-04 | -0.16 | 7.83E-01 | | -0.07 | 9.20E-01 | |  | | CRYZ | -0.67 | 2.58E-04 | | -0.19 | | 7.51E-01 | -0.35 | 3.39E-01 | |
| WDR41 | 0.67 | | 2.91E-06 | -0.20 | 5.59E-01 | | -0.06 | 9.20E-01 | |  | | AMOT | -0.67 | 3.94E-04 | | -0.81 | | 3.84E-05 | -0.31 | 4.23E-01 | |
| YTHDC1 | 0.67 | | 4.02E-05 | 0.03 | 9.65E-01 | | -0.02 | 9.73E-01 | |  | | PCDHGB5 | -0.67 | 7.96E-03 | | -0.20 | | 7.24E-01 | 0.20 | 6.84E-01 | |
| NUDT18 | 0.67 | | 7.21E-05 | -0.09 | 8.85E-01 | | 0.16 | 7.96E-01 | |  | | VANGL1 | -0.68 | 1.33E-05 | | -0.45 | | 3.41E-02 | -0.36 | 1.90E-01 | |
| DENND1A | 0.67 | | 8.78E-06 | -0.04 | 9.41E-01 | | 0.35 | 2.56E-01 | |  | | SGSM2 | -0.68 | 1.93E-05 | | -0.17 | | 6.70E-01 | -0.21 | 6.29E-01 | |
| FUT1 | 0.67 | | 2.81E-03 | 0.03 | 9.68E-01 | | 0.20 | 7.07E-01 | |  | | CDK2AP1 | -0.68 | 8.35E-07 | | -0.34 | | 9.56E-02 | -0.40 | 1.77E-01 | |
| ZBTB11 | 0.67 | | 6.35E-04 | -0.26 | 6.46E-01 | | -0.31 | 5.38E-01 | |  | | SPRYD4 | -0.68 | 2.16E-04 | | -0.05 | | 9.36E-01 | -0.23 | 5.78E-01 | |
| CD2AP | 0.66 | | 1.02E-05 | 0.04 | 9.60E-01 | | 0.25 | 6.60E-01 | |  | | TOP2B | -0.68 | 2.13E-05 | | -0.24 | | 4.72E-01 | -0.54 | 1.12E-01 | |
| FBXL4 | 0.66 | | 1.71E-03 | 0.31 | 4.55E-01 | | 0.10 | 8.86E-01 | |  | | SLC5A3 | -0.68 | 2.58E-03 | | -0.06 | | 9.40E-01 | -0.07 | 9.31E-01 | |
| DDX59 | 0.66 | | 1.04E-03 | -0.06 | 9.30E-01 | | -0.10 | 8.79E-01 | |  | | PIN4 | -0.68 | 4.57E-03 | | 0.26 | | 5.61E-01 | 0.18 | 7.25E-01 | |
| AP1AR | 0.66 | | 1.14E-04 | 0.12 | 8.64E-01 | | 0.11 | 8.78E-01 | |  | | GK5 | -0.69 | 2.22E-03 | | -0.09 | | 9.05E-01 | -0.30 | 5.67E-01 | |
| SHMT2 | 0.66 | | 2.47E-05 | -0.09 | 8.36E-01 | | 0.01 | 9.89E-01 | |  | | UFC1 | -0.69 | 8.75E-06 | | 0.06 | | 9.20E-01 | -0.23 | 5.27E-01 | |
| CAMSAP2 | 0.66 | | 3.29E-05 | 0.12 | 7.79E-01 | | 0.07 | 9.20E-01 | |  | | ELOVL6 | -0.69 | 1.69E-05 | | -0.01 | | 9.91E-01 | -0.02 | 9.66E-01 | |
| MEAK7 | 0.66 | | 1.10E-05 | 0.37 | 7.08E-02 | | 0.40 | 1.59E-01 | |  | | PASK | -0.69 | 2.94E-04 | | -0.22 | | 5.77E-01 | -0.12 | 8.35E-01 | |
| SNHG5 | 0.66 | | 4.35E-05 | 0.45 | 1.23E-01 | | 0.12 | 8.09E-01 | |  | | UQCRH | -0.69 | 4.93E-05 | | 0.08 | | 8.69E-01 | -0.18 | 6.49E-01 | |
| TWNK | 0.66 | | 1.82E-05 | 0.30 | 2.68E-01 | | 0.27 | 4.60E-01 | |  | | TRIM3 | -0.69 | 3.54E-03 | | 0.08 | | 9.15E-01 | 0.06 | 9.42E-01 | |
| ZNF592 | 0.66 | | 1.52E-04 | 0.20 | 5.79E-01 | | 0.29 | 4.86E-01 | |  | | ETNK2 | -0.69 | 7.67E-03 | | -0.32 | | 5.09E-01 | -0.58 | 1.13E-01 | |
| CREBBP | 0.66 | | 2.27E-03 | 0.13 | 8.37E-01 | | 0.22 | 6.78E-01 | |  | | STXBP4 | -0.69 | 1.90E-03 | | 0.07 | | 9.27E-01 | -0.44 | 3.23E-01 | |
| OARD1 | 0.66 | | 1.48E-04 | 0.18 | 6.72E-01 | | -0.14 | 8.07E-01 | |  | | OST4 | -0.69 | 3.15E-04 | | 0.36 | | 2.90E-01 | 0.16 | 7.85E-01 | |
| CEP95 | 0.66 | | 6.90E-05 | -0.07 | 9.10E-01 | | -0.22 | 6.49E-01 | |  | | HDHD3 | -0.70 | 6.32E-06 | | -0.12 | | 8.33E-01 | -0.13 | 8.09E-01 | |
| CCDC59 | 0.66 | | 3.78E-04 | 0.31 | 3.84E-01 | | 0.15 | 7.73E-01 | |  | | SNRPG | -0.70 | 6.65E-04 | | 0.38 | | 1.55E-01 | 0.18 | 7.30E-01 | |
| CCNJ | 0.66 | | 6.38E-03 | -0.17 | 8.18E-01 | | 0.06 | 9.39E-01 | |  | | SEMA4F | -0.70 | 1.98E-03 | | 0.19 | | 6.79E-01 | 0.18 | 7.26E-01 | |
| ZMYM5 | 0.66 | | 2.40E-04 | -0.21 | 6.11E-01 | | -0.39 | 2.98E-01 | |  | | COX7B | -0.70 | 3.59E-04 | | 0.00 | | 9.98E-01 | -0.25 | 4.98E-01 | |
| ABTB2 | 0.66 | | 7.25E-04 | -0.34 | 3.49E-01 | | -0.09 | 8.90E-01 | |  | | SCAND1 | -0.70 | 2.32E-05 | | 0.17 | | 7.98E-01 | 0.14 | 8.51E-01 | |
| AHR | 0.66 | | 2.80E-03 | 0.08 | 9.32E-01 | | -0.11 | 9.01E-01 | |  | | GPC1 | -0.70 | 2.26E-07 | | -0.26 | | 4.32E-01 | -0.25 | 5.95E-01 | |
| TMEM242 | 0.66 | | 6.24E-04 | 0.32 | 3.84E-01 | | 0.39 | 3.31E-01 | |  | | LPCAT1 | -0.70 | 5.69E-05 | | -0.03 | | 9.68E-01 | 0.38 | 2.00E-01 | |
| LNCAROD | 0.66 | | 2.15E-05 | 0.46 | 3.46E-02 | | 0.29 | 4.99E-01 | |  | | ABCE1 | -0.70 | 1.23E-07 | | -0.12 | | 8.19E-01 | -0.22 | 6.60E-01 | |
| MDM1 | 0.66 | | 4.50E-03 | -0.24 | 7.21E-01 | | -0.13 | 8.50E-01 | |  | | SUFU | -0.70 | 1.21E-03 | | 0.15 | | 7.70E-01 | -0.04 | 9.57E-01 | |
| TIMM9 | 0.66 | | 4.17E-04 | 0.32 | 3.55E-01 | | -0.04 | 9.61E-01 | |  | | GM2A | -0.70 | 3.37E-06 | | 0.24 | | 3.57E-01 | 0.22 | 5.00E-01 | |
| PPP3CA | 0.66 | | 6.28E-03 | 0.13 | 8.29E-01 | | 0.00 | 9.97E-01 | |  | | DNAJB1 | -0.71 | 8.31E-07 | | -0.15 | | 7.45E-01 | -0.19 | 6.66E-01 | |
| STX17 | 0.65 | | 1.64E-04 | -0.41 | 1.58E-01 | | -0.18 | 7.29E-01 | |  | | TUBB4B | -0.71 | 4.70E-06 | | 0.06 | | 9.25E-01 | 0.19 | 7.07E-01 | |
| RLIM | 0.65 | | 4.94E-03 | -0.22 | 6.86E-01 | | 0.09 | 9.01E-01 | |  | | COA3 | -0.71 | 1.02E-04 | | 0.22 | | 5.65E-01 | 0.22 | 6.52E-01 | |
| RIOK2 | 0.65 | | 1.15E-05 | 0.02 | 9.70E-01 | | -0.12 | 8.21E-01 | |  | | HMGB1P5 | -0.71 | 9.46E-03 | | 0.06 | | 9.49E-01 | -0.14 | 8.48E-01 | |
| MED13L | 0.65 | | 1.61E-04 | 0.25 | 4.63E-01 | | 0.23 | 5.99E-01 | |  | | MLEC | -0.71 | 1.73E-07 | | -0.05 | | 9.19E-01 | -0.06 | 9.02E-01 | |
| PPP1R15B | 0.65 | | 2.88E-04 | -0.23 | 5.42E-01 | | -0.02 | 9.74E-01 | |  | | MSMO1 | -0.72 | 7.71E-05 | | -0.49 | | 7.59E-02 | -0.28 | 5.74E-01 | |
| ACOT2 | 0.65 | | 1.61E-04 | 0.27 | 4.66E-01 | | 0.48 | 1.87E-01 | |  | | GALNT11 | -0.72 | 2.54E-03 | | -0.15 | | 7.51E-01 | -0.34 | 3.25E-01 | |
| TFAP2A | 0.65 | | 3.98E-04 | 0.11 | 8.43E-01 | | 0.47 | 1.38E-01 | |  | | RTN4R | -0.72 | 2.15E-03 | | -0.53 | | 8.91E-02 | -0.34 | 4.78E-01 | |
| WAC | 0.65 | | 2.16E-05 | -0.18 | 6.64E-01 | | -0.19 | 6.42E-01 | |  | | DAG1 | -0.72 | 1.57E-05 | | -0.36 | | 2.00E-01 | -0.43 | 1.82E-01 | |
| SNX33 | 0.65 | | 4.29E-05 | 0.30 | 2.34E-01 | | 0.41 | 1.74E-01 | |  | | MAGEF1 | -0.72 | 1.36E-07 | | -0.12 | | 7.52E-01 | -0.36 | 1.79E-01 | |
| IL6ST | 0.65 | | 4.63E-04 | -0.06 | 9.40E-01 | | 0.23 | 7.20E-01 | |  | | UBE4B | -0.72 | 1.02E-05 | | -0.18 | | 6.03E-01 | -0.16 | 6.98E-01 | |
| SMARCAD1 | 0.65 | | 3.10E-04 | 0.21 | 6.86E-01 | | 0.11 | 8.71E-01 | |  | | ALG1L | -0.72 | 2.46E-03 | | -0.33 | | 4.36E-01 | -0.42 | 3.41E-01 | |
| MR1 | 0.65 | | 4.73E-06 | 0.06 | 9.19E-01 | | 0.40 | 1.31E-01 | |  | | ACSL1 | -0.72 | 5.65E-05 | | -0.47 | | 6.15E-02 | -0.46 | 1.32E-01 | |
| CRCP | 0.65 | | 1.37E-05 | -0.11 | 7.92E-01 | | -0.13 | 7.69E-01 | |  | | UQCR10 | -0.72 | 1.51E-04 | | 0.01 | | 9.91E-01 | -0.16 | 7.59E-01 | |
| GOLT1B | 0.65 | | 1.13E-05 | 0.09 | 9.17E-01 | | 0.19 | 7.54E-01 | |  | | GLCE | -0.72 | 4.68E-04 | | -0.16 | | 7.99E-01 | -0.08 | 9.20E-01 | |
| ZNF121 | 0.64 | | 9.18E-04 | 0.25 | 5.39E-01 | | 0.04 | 9.66E-01 | |  | | CASP6 | -0.72 | 1.63E-04 | | -0.24 | | 5.51E-01 | -0.45 | 1.50E-01 | |
| GTPBP10 | 0.64 | | 3.63E-05 | 0.20 | 5.63E-01 | | 0.06 | 9.28E-01 | |  | | SYTL4 | -0.72 | 9.28E-03 | | 0.44 | | 1.94E-01 | 0.33 | 4.57E-01 | |
| MED10 | 0.64 | | 9.11E-06 | 0.19 | 5.85E-01 | | 0.25 | 4.58E-01 | |  | | CRACR2B | -0.73 | 3.61E-03 | | -0.36 | | 3.87E-01 | -0.36 | 4.22E-01 | |
| BNIP1 | 0.64 | | 1.14E-03 | -0.08 | 9.13E-01 | | -0.07 | 9.17E-01 | |  | | DPM3 | -0.73 | 5.80E-03 | | 0.07 | | 9.44E-01 | -0.14 | 8.57E-01 | |
| TRAK1 | 0.64 | | 1.43E-04 | 0.19 | 5.93E-01 | | 0.30 | 3.56E-01 | |  | | SMIM30 | -0.73 | 1.32E-03 | | 0.43 | | 1.32E-01 | 0.06 | 9.40E-01 | |
| LARP1 | 0.64 | | 1.89E-03 | 0.35 | 3.45E-01 | | 0.55 | 1.01E-01 | |  | | RHOV | -0.73 | 2.68E-04 | | -0.70 | | 9.58E-04 | -0.31 | 3.97E-01 | |
| MARS | 0.64 | | 2.11E-05 | -0.29 | 2.74E-01 | | -0.19 | 6.20E-01 | |  | | TAF9B | -0.73 | 1.85E-06 | | -0.36 | | 3.20E-01 | -0.55 | 1.82E-01 | |
| SDCCAG8 | 0.64 | | 2.92E-05 | 0.43 | 4.84E-02 | | 0.42 | 1.26E-01 | |  | | WSB2 | -0.73 | 4.11E-07 | | 0.05 | | 9.21E-01 | -0.12 | 7.88E-01 | |
| MET | 0.64 | | 2.24E-04 | 0.26 | 4.52E-01 | | 0.25 | 5.47E-01 | |  | | NTHL1 | -0.73 | 1.35E-05 | | 0.04 | | 9.54E-01 | 0.04 | 9.58E-01 | |
| LYPD6B | 0.64 | | 9.71E-05 | -0.67 | 1.93E-03 | | -0.30 | 3.98E-01 | |  | | PCNA | -0.73 | 8.51E-07 | | -0.18 | | 5.53E-01 | -0.07 | 8.83E-01 | |
| UPF2 | 0.64 | | 2.56E-04 | 0.04 | 9.59E-01 | | 0.14 | 8.07E-01 | |  | | PXDN | -0.73 | 1.15E-05 | | -0.22 | | 5.09E-01 | -0.32 | 3.57E-01 | |
| PTPDC1 | 0.64 | | 6.94E-03 | 0.01 | 9.87E-01 | | -0.01 | 9.82E-01 | |  | | LGALS1 | -0.73 | 5.70E-05 | | 0.24 | | 6.02E-01 | 0.03 | 9.73E-01 | |
| SNX11 | 0.64 | | 1.70E-04 | -0.12 | 8.19E-01 | | -0.14 | 7.65E-01 | |  | | POP4 | -0.73 | 1.83E-07 | | -0.04 | | 9.45E-01 | -0.07 | 8.78E-01 | |
| ZFP90 | 0.63 | | 1.27E-03 | -0.20 | 7.45E-01 | | -0.15 | 8.00E-01 | |  | | PRRC1 | -0.73 | 5.22E-06 | | -0.29 | | 3.97E-01 | -0.36 | 3.18E-01 | |
| CCSER2 | 0.63 | | 6.51E-05 | -0.36 | 1.51E-01 | | -0.47 | 1.43E-01 | |  | | TULP3 | -0.74 | 7.10E-06 | | -0.24 | | 4.20E-01 | -0.30 | 3.81E-01 | |
| MIS12 | 0.63 | | 5.73E-06 | -0.22 | 5.74E-01 | | -0.02 | 9.70E-01 | |  | | RBM12 | -0.74 | 6.01E-04 | | -0.28 | | 4.70E-01 | -0.23 | 6.45E-01 | |
| SDR42E1 | 0.63 | | 1.04E-04 | -0.13 | 8.04E-01 | | 0.06 | 9.28E-01 | |  | | BRK1 | -0.74 | 1.05E-07 | | -0.01 | | 9.83E-01 | -0.20 | 5.86E-01 | |
| BRCA2 | 0.63 | | 3.45E-05 | 0.19 | 5.97E-01 | | 0.20 | 6.87E-01 | |  | | CCNE1 | -0.74 | 7.97E-06 | | 0.13 | | 7.73E-01 | 0.33 | 2.67E-01 | |
| RIN2 | 0.63 | | 1.57E-04 | -0.15 | 7.96E-01 | | 0.12 | 8.07E-01 | |  | | GALNT5 | -0.74 | 4.39E-07 | | -0.37 | | 9.57E-02 | -0.30 | 3.46E-01 | |
| KPNA4 | 0.63 | | 3.52E-04 | 0.21 | 5.89E-01 | | 0.07 | 9.24E-01 | |  | | PHTF2 | -0.74 | 5.27E-07 | | -0.27 | | 5.53E-01 | -0.55 | 1.10E-01 | |
| NHSL1 | 0.63 | | 3.28E-03 | 0.21 | 5.67E-01 | | 0.18 | 6.92E-01 | |  | | TUBB | -0.74 | 1.68E-06 | | -0.11 | | 7.72E-01 | -0.13 | 7.60E-01 | |
| NOD1 | 0.63 | | 1.44E-03 | -0.15 | 7.94E-01 | | 0.40 | 2.74E-01 | |  | | SLFN11 | -0.74 | 3.02E-05 | | -0.09 | | 8.89E-01 | 0.45 | 1.47E-01 | |
| BIRC2 | 0.63 | | 6.50E-04 | -0.46 | 1.59E-01 | | -0.44 | 3.43E-01 | |  | | CDCA3 | -0.75 | 1.59E-06 | | -0.10 | | 7.98E-01 | -0.09 | 8.62E-01 | |
| ELAC1 | 0.63 | | 6.74E-04 | 0.07 | 9.20E-01 | | -0.12 | 8.44E-01 | |  | | SOCS2 | -0.75 | 2.18E-05 | | 0.54 | | 1.85E-02 | 0.26 | 4.88E-01 | |
| SIAH2 | 0.63 | | 5.03E-06 | -0.25 | 3.80E-01 | | -0.33 | 2.31E-01 | |  | | AIF1L | -0.75 | 1.43E-03 | | 0.03 | | 9.70E-01 | -0.30 | 5.29E-01 | |
| CNOT6L | 0.63 | | 4.99E-03 | 0.20 | 7.28E-01 | | 0.00 | 9.98E-01 | |  | | RPL23 | -0.75 | 2.16E-04 | | 0.31 | | 3.71E-01 | 0.13 | 7.97E-01 | |
| RBAK | 0.63 | | 5.62E-04 | -0.19 | 6.07E-01 | | -0.13 | 8.50E-01 | |  | | EPHA1 | -0.75 | 2.38E-06 | | 0.13 | | 7.38E-01 | 0.13 | 7.86E-01 | |
| MTF1 | 0.63 | | 1.21E-04 | 0.09 | 8.64E-01 | | 0.28 | 3.94E-01 | |  | | FASTKD1 | -0.75 | 5.65E-08 | | -0.16 | | 7.58E-01 | -0.30 | 5.08E-01 | |
| FABP5 | 0.63 | | 3.17E-03 | 0.70 | 3.30E-03 | | 0.46 | 2.59E-01 | |  | | POLR2L | -0.76 | 2.72E-04 | | 0.29 | | 4.70E-01 | 0.42 | 2.81E-01 | |
| TOGARAM1 | 0.63 | | 3.28E-03 | -0.20 | 6.70E-01 | | -0.27 | 6.13E-01 | |  | | RTL8C | -0.76 | 2.50E-05 | | 0.06 | | 9.30E-01 | -0.08 | 8.87E-01 | |
| NME6 | 0.63 | | 2.16E-04 | -0.01 | 9.84E-01 | | 0.17 | 7.59E-01 | |  | | ATP5MPL | -0.76 | 6.67E-06 | | 0.01 | | 9.88E-01 | -0.30 | 3.76E-01 | |
| IGF1R | 0.63 | | 2.63E-03 | -0.25 | 5.27E-01 | | 0.05 | 9.33E-01 | |  | | NDUFB4 | -0.76 | 6.15E-04 | | 0.06 | | 9.36E-01 | -0.04 | 9.54E-01 | |
| KIAA0753 | 0.63 | | 2.59E-04 | -0.06 | 9.25E-01 | | -0.03 | 9.54E-01 | |  | | HCN3 | -0.76 | 5.58E-03 | | -0.33 | | 4.82E-01 | -0.36 | 4.76E-01 | |
| ZNF131 | 0.63 | | 1.28E-04 | 0.06 | 9.19E-01 | | -0.10 | 8.47E-01 | |  | | MLLT11 | -0.77 | 2.22E-04 | | 0.16 | | 7.36E-01 | -0.04 | 9.50E-01 | |
| SPOPL | 0.63 | | 2.45E-03 | 0.13 | 8.69E-01 | | 0.18 | 8.09E-01 | |  | | EIF4E2 | -0.77 | 1.01E-07 | | -0.28 | | 2.15E-01 | -0.33 | 2.12E-01 | |
| ACSS1 | 0.62 | | 1.03E-03 | -0.63 | 1.51E-02 | | 0.39 | 2.92E-01 | |  | | RAB7B | -0.77 | 7.74E-05 | | 0.10 | | 8.48E-01 | -0.31 | 4.17E-01 | |
| LRRC8B | 0.62 | | 5.90E-04 | 0.16 | 7.36E-01 | | 0.40 | 2.20E-01 | |  | | CCNG1 | -0.77 | 3.49E-07 | | -0.12 | | 7.85E-01 | -0.27 | 5.45E-01 | |
| H1FX | 0.62 | | 7.54E-03 | 0.23 | 7.04E-01 | | 0.23 | 7.22E-01 | |  | | HSPB1 | -0.77 | 2.64E-04 | | -0.50 | | 1.48E-01 | -0.43 | 2.80E-01 | |
| ZNF33B | 0.62 | | 2.05E-04 | 0.09 | 8.80E-01 | | -0.04 | 9.49E-01 | |  | | DNAJA4 | -0.77 | 5.72E-06 | | 0.31 | | 2.84E-01 | 0.43 | 1.21E-01 | |
| CGGBP1 | 0.62 | | 3.57E-05 | -0.16 | 7.45E-01 | | -0.12 | 8.33E-01 | |  | | MACC1 | -0.77 | 3.53E-05 | | -0.23 | | 5.68E-01 | -0.41 | 3.39E-01 | |
| DMTF1 | 0.62 | | 2.27E-04 | -0.06 | 9.36E-01 | | 0.01 | 9.89E-01 | |  | | RECQL4 | -0.77 | 9.18E-07 | | -0.12 | | 8.12E-01 | -0.06 | 9.25E-01 | |
| OBI1 | 0.62 | | 4.99E-04 | -0.04 | 9.59E-01 | | 0.03 | 9.71E-01 | |  | | KBTBD7 | -0.77 | 1.27E-03 | | -0.07 | | 9.26E-01 | 0.11 | 8.42E-01 | |
| RASEF | 0.62 | | 7.54E-04 | -0.04 | 9.46E-01 | | 0.19 | 6.67E-01 | |  | | DPY19L1 | -0.77 | 3.87E-05 | | -0.11 | | 8.84E-01 | -0.17 | 7.89E-01 | |
| TIMM23B | 0.62 | | 2.92E-03 | 0.07 | 9.22E-01 | | 0.14 | 8.23E-01 | |  | | RBX1 | -0.77 | 1.65E-05 | | 0.12 | | 8.11E-01 | 0.01 | 9.82E-01 | |
| DDI2 | 0.62 | | 4.80E-05 | 0.05 | 9.30E-01 | | 0.10 | 8.56E-01 | |  | | FDXACB1 | -0.77 | 6.29E-03 | | -0.03 | | 9.74E-01 | -0.10 | 9.12E-01 | |
| IQCB1 | 0.62 | | 1.70E-05 | 0.03 | 9.68E-01 | | 0.00 | 9.99E-01 | |  | | SLC35G1 | -0.78 | 9.27E-06 | | -0.12 | | 8.53E-01 | -0.27 | 5.36E-01 | |
| CMTR1 | 0.62 | | 1.14E-04 | -0.03 | 9.67E-01 | | 0.44 | 1.32E-01 | |  | | KRT19 | -0.78 | 2.45E-09 | | -0.42 | | 2.82E-02 | -0.35 | 3.99E-01 | |
| CEP135 | 0.62 | | 6.46E-03 | 0.06 | 9.42E-01 | | 0.03 | 9.71E-01 | |  | | FAM8A1 | -0.78 | 1.88E-03 | | -0.18 | | 8.13E-01 | -0.60 | 1.23E-01 | |
| RNF19A | 0.62 | | 1.27E-03 | -0.25 | 6.10E-01 | | -0.03 | 9.65E-01 | |  | | ATP6V0E2 | -0.78 | 4.52E-08 | | -0.15 | | 7.34E-01 | -0.30 | 3.68E-01 | |
| FAM83F | 0.62 | | 3.28E-04 | 0.04 | 9.58E-01 | | 0.19 | 6.89E-01 | |  | | GGT1 | -0.78 | 8.40E-03 | | -0.02 | | 9.84E-01 | -0.25 | 7.12E-01 | |
| RAB11FIP2 | 0.62 | | 9.54E-03 | 0.11 | 8.84E-01 | | 0.15 | 8.36E-01 | |  | | SLC7A8 | -0.78 | 1.62E-04 | | 0.12 | | 8.26E-01 | 0.06 | 9.27E-01 | |
| ZFYVE9 | 0.61 | | 1.80E-03 | 0.46 | 8.69E-02 | | 0.37 | 3.25E-01 | |  | | ATP13A2 | -0.78 | 2.68E-08 | | 0.26 | | 4.09E-01 | 0.41 | 2.56E-01 | |
| POGZ | 0.61 | | 4.95E-04 | 0.12 | 8.00E-01 | | 0.05 | 9.44E-01 | |  | | ALAD | -0.78 | 4.63E-06 | | -0.41 | | 1.26E-01 | -0.43 | 2.12E-01 | |
| MBD1 | 0.61 | | 1.67E-05 | 0.09 | 8.33E-01 | | 0.22 | 5.74E-01 | |  | | PLIN3 | -0.78 | 1.53E-06 | | 0.36 | | 2.03E-01 | 0.51 | 1.59E-01 | |
| ZFP62 | 0.61 | | 1.52E-04 | -0.12 | 8.47E-01 | | -0.28 | 5.43E-01 | |  | | CAMK2N1 | -0.78 | 1.09E-05 | | 0.41 | | 1.07E-01 | 0.01 | 9.85E-01 | |
| C19orf48 | 0.61 | | 8.62E-07 | 0.25 | 4.61E-01 | | 0.42 | 2.92E-01 | |  | | CUL3 | -0.78 | 1.84E-09 | | -0.25 | | 4.52E-01 | -0.34 | 3.62E-01 | |
| ZNF600 | 0.61 | | 7.71E-04 | -0.06 | 9.24E-01 | | -0.24 | 5.52E-01 | |  | | TWSG1 | -0.79 | 8.29E-06 | | -0.48 | | 1.67E-01 | -0.51 | 1.13E-01 | |
| LRRFIP1 | 0.61 | | 4.01E-06 | 0.16 | 5.81E-01 | | 0.15 | 6.76E-01 | |  | | MT1X | -0.79 | 2.29E-04 | | 0.21 | | 6.26E-01 | 0.05 | 9.45E-01 | |
| GTPBP1 | 0.61 | | 1.35E-03 | 0.02 | 9.72E-01 | | 0.47 | 1.50E-01 | |  | | SPOCK1 | -0.79 | 5.05E-05 | | -0.05 | | 9.39E-01 | -0.41 | 1.97E-01 | |
| HSPBAP1 | 0.61 | | 3.62E-04 | 0.02 | 9.76E-01 | | 0.14 | 7.81E-01 | |  | | TTYH3 | -0.79 | 4.69E-03 | | 0.34 | | 5.29E-01 | 0.18 | 8.02E-01 | |
| CDK8 | 0.61 | | 6.99E-03 | 0.26 | 6.21E-01 | | 0.32 | 4.96E-01 | |  | | FSD1 | -0.79 | 4.16E-04 | | -0.05 | | 9.48E-01 | -0.08 | 9.12E-01 | |
| BRD4 | 0.61 | | 6.30E-03 | 0.17 | 7.66E-01 | | 0.22 | 6.81E-01 | |  | | PTGES | -0.79 | 2.85E-09 | | 0.38 | | 1.05E-01 | -0.16 | 7.18E-01 | |
| OSBPL10 | 0.61 | | 1.27E-04 | 0.39 | 8.52E-02 | | 0.39 | 1.54E-01 | |  | | LAMTOR5 | -0.79 | 5.37E-05 | | 0.20 | | 6.18E-01 | -0.17 | 7.41E-01 | |
| NEK9 | 0.61 | | 2.57E-03 | 0.17 | 7.51E-01 | | 0.21 | 6.45E-01 | |  | | KPNA2 | -0.79 | 1.17E-08 | | -0.23 | | 4.02E-01 | -0.20 | 5.51E-01 | |
| VPS37A | 0.61 | | 6.05E-05 | 0.09 | 8.99E-01 | | 0.16 | 7.64E-01 | |  | | HBS1L | -0.79 | 1.33E-07 | | -0.39 | | 1.63E-01 | -0.50 | 1.10E-01 | |
| RPGR | 0.61 | | 4.20E-03 | 0.31 | 4.51E-01 | | 0.32 | 4.79E-01 | |  | | MUC20-OT1 | -0.79 | 3.97E-03 | | -0.28 | | 5.53E-01 | -0.43 | 2.85E-01 | |
| CDYL | 0.61 | | 1.43E-03 | 0.04 | 9.58E-01 | | 0.14 | 7.82E-01 | |  | | UNC119B | -0.79 | 2.07E-05 | | 0.09 | | 8.58E-01 | -0.10 | 8.41E-01 | |
| ZNF268 | 0.61 | | 2.82E-03 | -0.12 | 8.57E-01 | | -0.09 | 9.08E-01 | |  | | NDUFS5 | -0.80 | 3.53E-05 | | 0.13 | | 8.00E-01 | -0.12 | 8.20E-01 | |
| ZNF24 | 0.61 | | 8.73E-06 | -0.12 | 7.72E-01 | | -0.04 | 9.42E-01 | |  | | ARSB | -0.80 | 4.68E-05 | | -0.02 | | 9.79E-01 | -0.01 | 9.93E-01 | |
| WRN | 0.61 | | 6.94E-03 | 0.12 | 8.63E-01 | | -0.26 | 6.46E-01 | |  | | TNRC6B | -0.80 | 1.55E-05 | | -0.23 | | 5.26E-01 | -0.35 | 3.01E-01 | |
| GOSR2 | 0.60 | | 5.14E-04 | -0.21 | 6.08E-01 | | 0.17 | 7.04E-01 | |  | | KANK2 | -0.80 | 7.85E-07 | | -0.41 | | 7.59E-02 | -0.31 | 4.70E-01 | |
| CEBPZ | 0.60 | | 2.98E-05 | 0.12 | 8.06E-01 | | 0.18 | 6.83E-01 | |  | | HSPA4L | -0.80 | 1.94E-06 | | -0.34 | | 3.53E-01 | -0.24 | 5.79E-01 | |
| TSC22D2 | 0.60 | | 1.41E-04 | -0.15 | 7.20E-01 | | 0.33 | 2.34E-01 | |  | | PAXIP1-AS1 | -0.80 | 4.20E-03 | | 0.05 | | 9.59E-01 | -0.49 | 2.24E-01 | |
| ORC5 | 0.60 | | 2.29E-05 | 0.01 | 9.90E-01 | | -0.17 | 7.50E-01 | |  | | PDK3 | -0.80 | 4.96E-04 | | -0.19 | | 6.98E-01 | -0.39 | 2.94E-01 | |
| TMEM170A | 0.60 | | 1.19E-03 | 0.45 | 7.73E-02 | | 0.58 | 1.02E-01 | |  | | SNRPD2 | -0.80 | 3.07E-06 | | 0.23 | | 5.29E-01 | 0.16 | 7.64E-01 | |
| LMO7 | 0.60 | | 1.92E-04 | -0.31 | 3.47E-01 | | 0.13 | 7.92E-01 | |  | | ALG6 | -0.81 | 8.66E-06 | | 0.02 | | 9.71E-01 | -0.10 | 8.86E-01 | |
| SELENOS | 0.60 | | 2.95E-04 | 0.19 | 6.12E-01 | | 0.35 | 2.48E-01 | |  | | PEX2 | -0.81 | 1.02E-05 | | -0.31 | | 2.95E-01 | -0.40 | 2.09E-01 | |
| PPP1R21 | 0.60 | | 1.18E-03 | 0.03 | 9.71E-01 | | 0.20 | 6.70E-01 | |  | | TMED9 | -0.81 | 4.75E-07 | | 0.22 | | 5.91E-01 | 0.02 | 9.65E-01 | |
| GAREM1 | 0.60 | | 1.62E-03 | 0.13 | 8.19E-01 | | 0.10 | 8.56E-01 | |  | | NUDT9 | -0.81 | 7.62E-05 | | -0.02 | | 9.76E-01 | -0.07 | 8.98E-01 | |
| SNX24 | 0.60 | | 5.66E-03 | 0.01 | 9.87E-01 | | -0.26 | 6.59E-01 | |  | | CHRNA5 | -0.81 | 1.27E-03 | | 0.15 | | 7.92E-01 | 0.17 | 7.86E-01 | |
| FBXL3 | 0.60 | | 1.33E-03 | -0.12 | 8.71E-01 | | -0.15 | 8.30E-01 | |  | | FEN1 | -0.81 | 1.44E-07 | | -0.20 | | 5.66E-01 | -0.13 | 8.03E-01 | |
| ZNF766 | 0.60 | | 5.06E-04 | -0.33 | 2.61E-01 | | -0.40 | 2.45E-01 | |  | | SYNM | -0.82 | 6.69E-03 | | -1.03 | | 1.94E-03 | -0.63 | 1.42E-01 | |
| REV3L | 0.59 | | 3.52E-04 | 0.00 | 9.98E-01 | | -0.08 | 9.01E-01 | |  | | SPC25 | -0.82 | 2.71E-05 | | -0.13 | | 7.81E-01 | -0.25 | 6.18E-01 | |
| ITFG2 | 0.59 | | 1.34E-04 | -0.08 | 8.74E-01 | | -0.18 | 7.05E-01 | |  | | ENO2 | -0.82 | 1.48E-03 | | -0.17 | | 7.78E-01 | 0.05 | 9.50E-01 | |
| NFX1 | 0.59 | | 1.42E-04 | -0.20 | 5.75E-01 | | -0.25 | 4.87E-01 | |  | | SLC4A3 | -0.82 | 3.39E-03 | | -0.02 | | 9.81E-01 | -0.10 | 8.95E-01 | |
| DNAJC1 | 0.59 | | 1.38E-03 | 0.14 | 7.95E-01 | | 0.31 | 4.23E-01 | |  | | SLC23A2 | -0.82 | 3.27E-06 | | 0.12 | | 7.90E-01 | 0.01 | 9.89E-01 | |
| POLR2M | 0.59 | | 4.50E-03 | -0.07 | 9.67E-01 | | 0.01 | 9.95E-01 | |  | | EID1 | -0.82 | 1.98E-08 | | 0.04 | | 9.45E-01 | -0.22 | 5.21E-01 | |
| LONP1 | 0.59 | | 2.47E-05 | -0.14 | 7.27E-01 | | 0.09 | 8.69E-01 | |  | | PHF10 | -0.82 | 3.85E-06 | | 0.09 | | 8.72E-01 | -0.37 | 2.85E-01 | |
| CFAP97 | 0.59 | | 4.74E-04 | 0.23 | 5.29E-01 | | 0.13 | 8.31E-01 | |  | | ATPSCKMT | -0.82 | 7.71E-05 | | -0.01 | | 9.87E-01 | -0.34 | 4.15E-01 | |
| CRTC2 | 0.59 | | 2.22E-04 | 0.23 | 4.98E-01 | | 0.46 | 1.13E-01 | |  | | CAPN8 | -0.82 | 5.41E-03 | | 1.46 | | 9.73E-14 | 0.47 | 2.73E-01 | |
| EIF5 | 0.59 | | 3.15E-04 | 0.14 | 7.55E-01 | | -0.17 | 7.00E-01 | |  | | EFNA4 | -0.82 | 1.64E-04 | | -0.37 | | 3.20E-01 | -0.23 | 6.64E-01 | |
| TASOR2 | 0.59 | | 5.48E-04 | -0.10 | 8.58E-01 | | 0.18 | 7.14E-01 | |  | | ZDHHC20 | -0.83 | 6.50E-04 | | -0.21 | | 9.06E-01 | -0.32 | 6.18E-01 | |
| PATZ1 | 0.59 | | 3.47E-03 | 0.17 | 7.43E-01 | | 0.38 | 2.90E-01 | |  | | RBIS | -0.83 | 1.71E-03 | | 0.18 | | 7.74E-01 | -0.02 | 9.78E-01 | |
| YAE1 | 0.59 | | 2.39E-03 | 0.23 | 5.59E-01 | | 0.13 | 8.26E-01 | |  | | SDHAP1 | -0.83 | 2.50E-05 | | -0.35 | | 3.23E-01 | -0.28 | 5.54E-01 | |
| NEK7 | 0.59 | | 1.99E-03 | -0.27 | 8.47E-01 | | 0.23 | 7.44E-01 | |  | | MRPL36 | -0.83 | 5.07E-06 | | 0.24 | | 5.20E-01 | 0.01 | 9.85E-01 | |
| MORC3 | 0.59 | | 1.00E-04 | 0.02 | 9.76E-01 | | 0.42 | 2.83E-01 | |  | | LRRC49 | -0.83 | 9.59E-04 | | -0.01 | | 9.87E-01 | -0.32 | 5.33E-01 | |
| RBM48 | 0.59 | | 8.87E-04 | -0.05 | 9.44E-01 | | -0.02 | 9.76E-01 | |  | | OBSL1 | -0.84 | 4.09E-05 | | -0.23 | | 6.53E-01 | -0.34 | 5.33E-01 | |
| ZNF41 | 0.59 | | 2.86E-03 | -0.20 | 7.16E-01 | | -0.11 | 8.70E-01 | |  | | TCEAL1 | -0.84 | 1.62E-04 | | -0.04 | | 9.59E-01 | -0.34 | 3.75E-01 | |
| INPP5E | 0.59 | | 2.99E-04 | 0.21 | 6.39E-01 | | 0.29 | 5.38E-01 | |  | | UBTD1 | -0.84 | 2.70E-06 | | 0.21 | | 6.65E-01 | 0.16 | 7.79E-01 | |
| DNASE2 | 0.59 | | 7.59E-05 | -0.21 | 5.15E-01 | | -0.16 | 7.47E-01 | |  | | SLC6A6 | -0.84 | 1.57E-04 | | -0.06 | | 9.38E-01 | 0.03 | 9.65E-01 | |
| SLC9A8 | 0.59 | | 3.54E-03 | -0.08 | 9.19E-01 | | 0.14 | 7.97E-01 | |  | | FASTKD5 | -0.84 | 1.50E-07 | | -0.19 | | 6.50E-01 | -0.15 | 7.57E-01 | |
| BET1 | 0.58 | | 8.89E-03 | 0.53 | 1.06E-01 | | 0.44 | 3.95E-01 | |  | | LINC02210 | -0.84 | 2.01E-03 | | -0.07 | | 9.39E-01 | -0.30 | 5.56E-01 | |
| EIF4A2 | 0.58 | | 1.24E-04 | 0.04 | 9.40E-01 | | 0.07 | 9.21E-01 | |  | | CBX1 | -0.84 | 1.21E-06 | | -0.12 | | 7.88E-01 | -0.32 | 2.75E-01 | |
| IBTK | 0.58 | | 3.83E-04 | -0.12 | 8.05E-01 | | -0.25 | 6.18E-01 | |  | | SPC24 | -0.84 | 4.68E-05 | | -0.34 | | 3.14E-01 | -0.18 | 7.32E-01 | |
| RNF168 | 0.58 | | 7.96E-05 | -0.26 | 3.42E-01 | | -0.30 | 3.43E-01 | |  | | KBTBD4 | -0.85 | 1.77E-04 | | -0.25 | | 5.67E-01 | -0.16 | 7.81E-01 | |
| RRN3 | 0.58 | | 1.60E-04 | -0.08 | 8.73E-01 | | 0.03 | 9.65E-01 | |  | | S1PR5 | -0.85 | 2.70E-05 | | -0.30 | | 3.37E-01 | -0.18 | 7.34E-01 | |
| C9orf85 | 0.58 | | 4.25E-03 | -0.22 | 6.24E-01 | | -0.24 | 6.46E-01 | |  | | RPS28 | -0.85 | 3.86E-04 | | 0.38 | | 3.21E-01 | 0.47 | 2.67E-01 | |
| SCYL3 | 0.58 | | 3.54E-03 | -0.24 | 6.04E-01 | | -0.09 | 8.93E-01 | |  | | AGPAT5 | -0.85 | 3.32E-11 | | 0.06 | | 9.19E-01 | -0.09 | 8.62E-01 | |
| PHF21A | 0.58 | | 3.93E-03 | 0.06 | 9.34E-01 | | -0.34 | 4.23E-01 | |  | | MAGED1 | -0.86 | 2.85E-07 | | -0.28 | | 3.49E-01 | -0.37 | 2.27E-01 | |
| TADA2A | 0.58 | | 7.28E-04 | -0.07 | 8.99E-01 | | -0.09 | 8.68E-01 | |  | | TIMM21 | -0.86 | 9.38E-10 | | -0.08 | | 8.76E-01 | -0.31 | 3.43E-01 | |
| ZNF639 | 0.58 | | 1.29E-04 | -0.02 | 9.75E-01 | | 0.07 | 9.12E-01 | |  | | ATP5MC1 | -0.86 | 4.91E-06 | | 0.07 | | 9.19E-01 | -0.01 | 9.90E-01 | |
| DPYD | 0.58 | | 6.04E-04 | -0.28 | 4.44E-01 | | 0.10 | 8.62E-01 | |  | | ABHD17A | -0.86 | 3.09E-05 | | 0.29 | | 4.83E-01 | 0.46 | 2.63E-01 | |
| SAMD4A | 0.58 | | 4.82E-03 | 0.32 | 3.34E-01 | | 0.24 | 5.60E-01 | |  | | PHF13 | -0.86 | 2.53E-06 | | -0.49 | | 4.12E-02 | -0.44 | 1.43E-01 | |
| RAPGEF1 | 0.58 | | 1.13E-03 | -0.08 | 8.71E-01 | | 0.01 | 9.88E-01 | |  | | LOXL1 | -0.87 | 1.17E-07 | | -0.12 | | 8.07E-01 | -0.23 | 6.37E-01 | |
| COQ10B | 0.58 | | 1.17E-03 | 0.14 | 7.91E-01 | | 0.41 | 3.28E-01 | |  | | ACPP | -0.87 | 3.36E-03 | | -0.64 | | 1.28E-01 | -0.45 | 3.43E-01 | |
| SUPT20H | 0.58 | | 3.97E-04 | -0.01 | 9.93E-01 | | 0.13 | 7.88E-01 | |  | | GINS1 | -0.87 | 1.61E-06 | | -0.40 | | 1.91E-01 | -0.37 | 3.68E-01 | |
| PPP1R12A | 0.57 | | 1.88E-05 | -0.19 | 5.34E-01 | | 0.02 | 9.78E-01 | |  | | ABCB9 | -0.87 | 1.07E-03 | | 0.11 | | 8.68E-01 | 0.18 | 7.83E-01 | |
| RAB38 | 0.57 | | 4.08E-05 | 0.24 | 4.09E-01 | | 0.28 | 3.09E-01 | |  | | ANKRD65 | -0.87 | 8.17E-04 | | 0.37 | | 3.56E-01 | 0.42 | 3.59E-01 | |
| ADGRG6 | 0.57 | | 1.90E-03 | 0.13 | 8.23E-01 | | 0.25 | 6.43E-01 | |  | | LSS | -0.87 | 5.32E-10 | | -0.42 | | 5.81E-02 | -0.46 | 1.48E-01 | |
| INTS12 | 0.57 | | 1.40E-03 | -0.07 | 9.22E-01 | | -0.13 | 8.16E-01 | |  | | DFFB | -0.87 | 8.87E-04 | | -0.02 | | 9.76E-01 | -0.13 | 8.32E-01 | |
| RCOR3 | 0.57 | | 1.24E-04 | 0.05 | 9.27E-01 | | -0.19 | 6.62E-01 | |  | | RPS29 | -0.87 | 9.74E-05 | | 0.33 | | 4.22E-01 | 0.22 | 6.83E-01 | |
| NAT9 | 0.57 | | 3.51E-04 | 0.14 | 7.72E-01 | | 0.16 | 7.29E-01 | |  | | CDH24 | -0.87 | 3.87E-09 | | -0.03 | | 9.58E-01 | -0.14 | 7.79E-01 | |
| POLK | 0.57 | | 6.46E-03 | 0.09 | 8.99E-01 | | 0.04 | 9.57E-01 | |  | | C9orf116 | -0.87 | 6.51E-03 | | -0.19 | | 7.95E-01 | -0.66 | 1.24E-01 | |
| SMIM14 | 0.57 | | 8.20E-03 | -0.64 | 2.22E-02 | | 0.05 | 9.42E-01 | |  | | VWA1 | -0.87 | 8.12E-03 | | 0.49 | | 2.52E-01 | 0.37 | 5.08E-01 | |
| EIF1 | 0.57 | | 8.54E-05 | 0.22 | 4.84E-01 | | 0.10 | 8.35E-01 | |  | | SH3PXD2B | -0.88 | 6.65E-03 | | 0.18 | | 7.89E-01 | 0.05 | 9.50E-01 | |
| EPRS | 0.57 | | 1.92E-05 | -0.16 | 5.89E-01 | | -0.30 | 4.15E-01 | |  | | GALK1 | -0.88 | 3.09E-05 | | -0.54 | | 8.66E-02 | -0.51 | 2.86E-01 | |
| LRATD2 | 0.57 | | 7.88E-05 | 0.20 | 5.39E-01 | | 0.18 | 6.50E-01 | |  | | GRHL3 | -0.88 | 4.70E-09 | | -0.28 | | 3.03E-01 | -0.13 | 7.86E-01 | |
| GATAD2B | 0.57 | | 7.92E-03 | 0.05 | 9.44E-01 | | 0.07 | 9.20E-01 | |  | | CPOX | -0.88 | 4.40E-05 | | -0.19 | | 7.25E-01 | -0.21 | 7.06E-01 | |
| MEF2A | 0.57 | | 2.87E-04 | 0.02 | 9.76E-01 | | -0.05 | 9.32E-01 | |  | | MICA | -0.89 | 2.17E-04 | | 0.26 | | 5.53E-01 | -0.20 | 7.19E-01 | |
| ATF2 | 0.57 | | 6.35E-04 | -0.35 | 4.52E-01 | | -0.13 | 8.20E-01 | |  | | POMT1 | -0.89 | 1.30E-07 | | -0.42 | | 9.27E-02 | -0.47 | 1.53E-01 | |
| OTUD6B | 0.57 | | 4.22E-03 | 0.31 | 3.96E-01 | | 0.17 | 8.26E-01 | |  | | MYL6 | -0.89 | 1.25E-08 | | 0.10 | | 8.44E-01 | 0.14 | 7.82E-01 | |
| ZBTB21 | 0.57 | | 7.86E-03 | -0.27 | 5.71E-01 | | -0.21 | 7.52E-01 | |  | | RPL38 | -0.89 | 1.65E-05 | | 0.31 | | 3.86E-01 | 0.19 | 6.82E-01 | |
| OGA | 0.57 | | 1.64E-04 | -0.08 | 8.68E-01 | | -0.06 | 9.23E-01 | |  | | ARHGEF10L | -0.89 | 7.51E-05 | | -0.64 | | 1.87E-02 | -0.59 | 1.29E-01 | |
| E2F7 | 0.57 | | 5.50E-04 | 0.46 | 6.20E-02 | | 0.33 | 3.37E-01 | |  | | ATP11A | -0.89 | 4.09E-07 | | -0.01 | | 9.81E-01 | -0.08 | 8.82E-01 | |
| PIP5K1A | 0.57 | | 2.30E-04 | -0.07 | 8.96E-01 | | 0.04 | 9.46E-01 | |  | | RHPN1 | -0.89 | 7.03E-03 | | -0.23 | | 7.91E-01 | -0.11 | 8.89E-01 | |
| DENND2D | 0.57 | | 3.92E-04 | -0.36 | 1.62E-01 | | 0.38 | 1.92E-01 | |  | | GCSH | -0.90 | 2.06E-05 | | -0.13 | | 8.30E-01 | -0.42 | 3.31E-01 | |
| ADIRF-AS1 | 0.57 | | 7.14E-03 | 0.60 | 8.72E-03 | | 0.47 | 1.40E-01 | |  | | CABLES2 | -0.90 | 2.22E-07 | | -0.31 | | 2.75E-01 | -0.07 | 9.19E-01 | |
| AASDHPPT | 0.56 | | 7.04E-05 | 0.19 | 6.67E-01 | | 0.13 | 8.28E-01 | |  | | TMSB4X | -0.90 | 1.02E-10 | | -0.03 | | 9.67E-01 | -0.34 | 2.23E-01 | |
| BMP2 | 0.56 | | 3.06E-03 | -0.91 | 8.14E-05 | | -0.45 | 1.44E-01 | |  | | ALOX12-AS1 | -0.90 | 6.16E-03 | | 0.05 | | 9.59E-01 | -0.32 | 5.91E-01 | |
| DGLUCY | 0.56 | | 2.18E-04 | -0.20 | 5.71E-01 | | 0.13 | 7.91E-01 | |  | | DBI | -0.90 | 1.72E-06 | | -0.06 | | 9.29E-01 | -0.36 | 2.90E-01 | |
| PHGDH | 0.56 | | 2.39E-04 | -0.53 | 1.54E-02 | | -0.51 | 1.03E-01 | |  | | LSM7 | -0.90 | 1.64E-06 | | 0.21 | | 7.00E-01 | 0.15 | 8.11E-01 | |
| ZC3H7B | 0.56 | | 8.19E-03 | 0.32 | 3.93E-01 | | 0.48 | 1.92E-01 | |  | | ERVK13-1 | -0.90 | 1.15E-03 | | -0.07 | | 9.34E-01 | -0.32 | 5.47E-01 | |
| CKMT1A | 0.56 | | 5.30E-04 | 0.15 | 7.32E-01 | | 0.41 | 1.55E-01 | |  | | POLE2 | -0.90 | 2.99E-08 | | -0.05 | | 9.30E-01 | -0.27 | 5.47E-01 | |
| CHMP4C | 0.56 | | 1.20E-03 | 0.26 | 4.64E-01 | | 0.09 | 8.77E-01 | |  | | ATP5MC3 | -0.91 | 7.94E-08 | | -0.13 | | 7.63E-01 | -0.29 | 3.81E-01 | |
| PTPN14 | 0.56 | | 6.24E-04 | 0.09 | 8.20E-01 | | 0.02 | 9.64E-01 | |  | | ISYNA1 | -0.91 | 1.97E-09 | | -0.25 | | 5.27E-01 | -0.30 | 5.78E-01 | |
| SINHCAF | 0.56 | | 1.91E-04 | 0.16 | 6.93E-01 | | 0.14 | 7.82E-01 | |  | | SPATC1L | -0.91 | 6.26E-05 | | 0.34 | | 4.61E-01 | 0.15 | 8.26E-01 | |
| MKKS | 0.56 | | 1.28E-04 | -0.01 | 9.81E-01 | | -0.29 | 3.43E-01 | |  | | SIRPA | -0.91 | 1.95E-03 | | 0.35 | | 4.67E-01 | 0.33 | 5.19E-01 | |
| PICALM | 0.56 | | 1.68E-04 | 0.16 | 7.09E-01 | | 0.20 | 7.03E-01 | |  | | CD1D | -0.92 | 4.67E-04 | | 0.20 | | 7.15E-01 | -0.54 | 1.61E-01 | |
| ORC6 | 0.56 | | 4.58E-04 | 0.06 | 9.19E-01 | | 0.08 | 8.73E-01 | |  | | CCDC88B | -0.92 | 2.34E-03 | | -0.29 | | 6.35E-01 | -0.33 | 5.66E-01 | |
| DTWD1 | 0.56 | | 2.57E-03 | 0.14 | 7.84E-01 | | -0.29 | 5.97E-01 | |  | | FAM217B | -0.93 | 3.38E-08 | | 0.12 | | 7.94E-01 | 0.01 | 9.85E-01 | |
| ZBTB2 | 0.56 | | 1.82E-03 | -0.30 | 4.63E-01 | | -0.23 | 6.11E-01 | |  | | PNPLA6 | -0.93 | 4.78E-05 | | 0.15 | | 8.05E-01 | 0.37 | 5.08E-01 | |
| SMC6 | 0.56 | | 1.32E-04 | -0.09 | 8.61E-01 | | -0.14 | 7.83E-01 | |  | | BRI3BP | -0.93 | 1.42E-12 | | 0.06 | | 9.17E-01 | 0.08 | 8.64E-01 | |
| ANKRD11 | 0.56 | | 1.25E-05 | 0.09 | 8.56E-01 | | 0.18 | 6.50E-01 | |  | | NDUFA2 | -0.93 | 1.89E-06 | | -0.05 | | 9.46E-01 | -0.05 | 9.38E-01 | |
| ZNF79 | 0.56 | | 6.58E-03 | -0.07 | 9.25E-01 | | 0.03 | 9.67E-01 | |  | | ENTPD4 | -0.93 | 2.25E-08 | | -0.01 | | 9.87E-01 | -0.12 | 8.12E-01 | |
| IER2 | 0.55 | | 5.70E-04 | 0.04 | 9.58E-01 | | 0.14 | 7.51E-01 | |  | | PCYOX1 | -0.93 | 5.14E-11 | | -0.16 | | 7.05E-01 | -0.38 | 2.38E-01 | |
| ZNF473 | 0.55 | | 1.36E-03 | 0.07 | 9.02E-01 | | 0.07 | 9.02E-01 | |  | | RPL7A | -0.94 | 2.01E-09 | | -0.01 | | 9.79E-01 | -0.09 | 8.65E-01 | |
| PWWP2B | 0.55 | | 3.27E-03 | 0.27 | 5.50E-01 | | 0.40 | 3.61E-01 | |  | | HIST1H1A | -0.94 | 1.41E-03 | | 0.23 | | 6.70E-01 | -0.04 | 9.62E-01 | |
| CCDC50 | 0.55 | | 8.00E-05 | -0.18 | 5.87E-01 | | -0.11 | 8.23E-01 | |  | | MAT2A | -0.94 | 2.21E-10 | | -0.02 | | 9.76E-01 | -0.07 | 8.89E-01 | |
| SYTL1 | 0.55 | | 2.85E-04 | -0.16 | 7.24E-01 | | 0.00 | 9.98E-01 | |  | | SERPINH1 | -0.95 | 4.45E-11 | | -0.38 | | 5.66E-02 | -0.42 | 1.74E-01 | |
| CIR1 | 0.55 | | 4.30E-03 | -0.11 | 8.46E-01 | | 0.07 | 9.06E-01 | |  | | SYT8 | -0.95 | 1.58E-03 | | -0.16 | | 8.48E-01 | -0.77 | 1.05E-01 | |
| TMEM216 | 0.55 | | 4.29E-04 | 0.31 | 2.66E-01 | | 0.27 | 4.58E-01 | |  | | FZD2 | -0.95 | 2.30E-04 | | -0.14 | | 8.48E-01 | -0.25 | 6.98E-01 | |
| LINS1 | 0.55 | | 4.83E-04 | -0.07 | 9.26E-01 | | 0.21 | 7.03E-01 | |  | | PXMP4 | -0.95 | 1.23E-07 | | -0.39 | | 1.47E-01 | -0.50 | 1.09E-01 | |
| ODR4 | 0.55 | | 4.35E-04 | -0.02 | 9.73E-01 | | 0.06 | 9.32E-01 | |  | | DLEU1 | -0.95 | 1.09E-03 | | 0.05 | | 9.59E-01 | -0.53 | 2.35E-01 | |
| CKAP2L | 0.55 | | 8.54E-05 | -0.03 | 9.54E-01 | | -0.27 | 5.22E-01 | |  | | ZNF287 | -0.95 | 1.77E-04 | | -0.17 | | 8.03E-01 | -0.24 | 6.49E-01 | |
| ZNF213 | 0.55 | | 2.80E-03 | 0.15 | 7.86E-01 | | 0.32 | 4.95E-01 | |  | | SLC26A2 | -0.96 | 2.00E-06 | | -0.19 | | 7.20E-01 | -0.53 | 1.93E-01 | |
| BET1L | 0.55 | | 4.70E-03 | 0.36 | 2.73E-01 | | 0.31 | 4.72E-01 | |  | | PROM2 | -0.96 | 6.13E-09 | | -0.05 | | 9.44E-01 | 0.00 | 9.95E-01 | |
| SETD5 | 0.55 | | 6.09E-04 | -0.10 | 8.19E-01 | | -0.20 | 6.05E-01 | |  | | SNHG10 | -0.97 | 1.39E-04 | | 0.02 | | 9.79E-01 | -0.31 | 5.27E-01 | |
| BMI1 | 0.55 | | 6.59E-03 | 0.02 | 9.85E-01 | | -0.03 | 9.69E-01 | |  | | MAGEH1 | -0.97 | 2.43E-03 | | 0.14 | | 8.51E-01 | -0.05 | 9.53E-01 | |
| SMG1 | 0.54 | | 4.60E-03 | -0.08 | 8.92E-01 | | 0.12 | 8.36E-01 | |  | | NDN | -0.97 | 3.77E-03 | | -0.49 | | 3.38E-01 | -0.11 | 8.92E-01 | |
| CSNK1G3 | 0.54 | | 3.03E-04 | -0.18 | 7.42E-01 | | -0.05 | 9.49E-01 | |  | | NMB | -0.97 | 7.81E-05 | | 0.31 | | 4.39E-01 | 0.28 | 5.67E-01 | |
| NMRK1 | 0.54 | | 1.36E-03 | 0.22 | 5.29E-01 | | 0.11 | 8.37E-01 | |  | | HTR1D | -0.98 | 4.02E-03 | | -0.82 | | 3.13E-02 | -0.54 | 2.95E-01 | |
| ESRP1 | 0.54 | | 1.73E-03 | -0.17 | 7.06E-01 | | -0.14 | 7.44E-01 | |  | | MXD3 | -0.98 | 1.48E-03 | | -0.39 | | 4.75E-01 | -0.56 | 2.24E-01 | |
| TMEM42 | 0.54 | | 7.99E-03 | 0.08 | 9.15E-01 | | -0.05 | 9.50E-01 | |  | | TNFRSF10D | -0.98 | 4.24E-03 | | 1.01 | | 2.41E-04 | 0.29 | 6.46E-01 | |
| ITPKC | 0.54 | | 4.92E-04 | -0.44 | 5.00E-02 | | -0.06 | 9.30E-01 | |  | | SLC12A2 | -0.98 | 3.94E-11 | | -0.31 | | 2.87E-01 | -0.46 | 1.54E-01 | |
| ZNF142 | 0.54 | | 3.01E-03 | 0.15 | 7.17E-01 | | 0.39 | 1.95E-01 | |  | | CACFD1 | -0.99 | 7.54E-03 | | -0.44 | | 5.66E-01 | -0.71 | 2.26E-01 | |
| ANAPC10 | 0.54 | | 8.74E-03 | 0.25 | 5.44E-01 | | 0.18 | 7.59E-01 | |  | | SF3A3 | -1.00 | 2.83E-10 | | 0.06 | | 9.17E-01 | -0.02 | 9.66E-01 | |
| XPR1 | 0.54 | | 3.28E-04 | -0.29 | 3.68E-01 | | -0.04 | 9.50E-01 | |  | | BRAT1 | -1.00 | 6.01E-11 | | 0.01 | | 9.80E-01 | 0.07 | 9.20E-01 | |
| FAM83B | 0.54 | | 2.15E-03 | 0.07 | 9.19E-01 | | -0.05 | 9.48E-01 | |  | | C4orf3 | -1.00 | 1.29E-08 | | -0.09 | | 8.66E-01 | -0.48 | 2.04E-01 | |
| NFE2L1 | 0.54 | | 2.88E-04 | 0.01 | 9.86E-01 | | 0.20 | 6.06E-01 | |  | | RCN2 | -1.00 | 4.77E-14 | | 0.08 | | 8.59E-01 | -0.36 | 3.07E-01 | |
| ASB7 | 0.54 | | 4.00E-03 | -0.19 | 7.52E-01 | | 0.09 | 8.83E-01 | |  | | SESTD1 | -1.00 | 6.82E-06 | | -0.18 | | 7.41E-01 | -0.53 | 1.13E-01 | |
| SLC3A2 | 0.54 | | 1.62E-04 | -0.12 | 7.98E-01 | | -0.17 | 7.25E-01 | |  | | MED11 | -1.00 | 7.00E-07 | | 0.01 | | 9.90E-01 | 0.02 | 9.75E-01 | |
| INPP1 | 0.54 | | 9.27E-04 | 0.16 | 6.86E-01 | | 0.36 | 2.00E-01 | |  | | SLC35D1 | -1.01 | 4.16E-08 | | -0.16 | | 7.65E-01 | -0.11 | 8.68E-01 | |
| NUP50-DT | 0.54 | | 4.85E-03 | 0.10 | 8.66E-01 | | 0.15 | 7.80E-01 | |  | | PIMREG | -1.01 | 5.70E-12 | | -0.33 | | 1.78E-01 | -0.24 | 5.29E-01 | |
| SMG7 | 0.54 | | 2.55E-04 | -0.09 | 8.29E-01 | | 0.02 | 9.70E-01 | |  | | MAP6D1 | -1.01 | 5.35E-04 | | -0.02 | | 9.84E-01 | -0.24 | 6.76E-01 | |
| MOB3B | 0.54 | | 5.09E-03 | -0.31 | 3.74E-01 | | 0.05 | 9.36E-01 | |  | | CHST14 | -1.02 | 2.42E-04 | | 0.04 | | 9.64E-01 | -0.21 | 7.77E-01 | |
| TEAD4 | 0.54 | | 4.96E-05 | -0.04 | 9.47E-01 | | 0.23 | 6.06E-01 | |  | | EPHB2 | -1.02 | 1.47E-08 | | -0.38 | | 1.20E-01 | -0.34 | 3.43E-01 | |
| TRAF6 | 0.54 | | 9.75E-03 | -0.22 | 6.38E-01 | | 0.08 | 9.00E-01 | |  | | DPYSL2 | -1.02 | 1.98E-12 | | -0.07 | | 9.04E-01 | -0.37 | 1.80E-01 | |
| SPTLC3 | 0.54 | | 9.73E-04 | 0.03 | 9.70E-01 | | 0.34 | 2.57E-01 | |  | | FLRT3 | -1.02 | 2.38E-06 | | 0.31 | | 4.14E-01 | 0.38 | 3.35E-01 | |
| ZNF252P | 0.54 | | 5.28E-03 | -0.08 | 8.92E-01 | | -0.07 | 9.12E-01 | |  | | GALR2 | -1.03 | 4.67E-03 | | -0.70 | | 1.36E-01 | -0.54 | 3.84E-01 | |
| SIX4 | 0.54 | | 3.53E-03 | 0.04 | 9.67E-01 | | 0.32 | 4.74E-01 | |  | | POLH | -1.05 | 2.50E-06 | | -0.25 | | 4.85E-01 | -0.28 | 4.83E-01 | |
| FOXJ3 | 0.54 | | 1.95E-03 | 0.04 | 9.49E-01 | | 0.18 | 7.02E-01 | |  | | ERCC6L | -1.06 | 1.12E-07 | | -0.43 | | 2.90E-01 | -0.35 | 4.51E-01 | |
| HEATR6 | 0.54 | | 2.61E-03 | -0.01 | 9.87E-01 | | 0.30 | 4.52E-01 | |  | | MAPKAP1 | -1.07 | 1.23E-14 | | 0.06 | | 9.13E-01 | -0.16 | 6.98E-01 | |
| TTC9C | 0.54 | | 8.12E-04 | -0.08 | 8.99E-01 | | 0.06 | 9.21E-01 | |  | | ZBED5-AS1 | -1.07 | 3.00E-07 | | -0.15 | | 7.81E-01 | -0.50 | 1.02E-01 | |
| CLIP4 | 0.53 | | 1.91E-03 | -0.02 | 9.78E-01 | | 0.02 | 9.67E-01 | |  | | MIR22HG | -1.07 | 1.15E-05 | | 0.09 | | 8.99E-01 | 0.13 | 8.17E-01 | |
| KIAA0040 | 0.53 | | 3.88E-03 | 0.31 | 3.66E-01 | | 0.31 | 4.04E-01 | |  | | MED31 | -1.07 | 1.74E-06 | | 0.15 | | 7.78E-01 | -0.18 | 7.63E-01 | |
| MED22 | 0.53 | | 2.02E-04 | 0.04 | 9.40E-01 | | 0.06 | 9.08E-01 | |  | | LOC150051 | -1.08 | 3.14E-03 | | -0.59 | | 2.34E-01 | -0.59 | 2.77E-01 | |
| DYRK4 | 0.53 | | 2.04E-03 | 0.12 | 8.17E-01 | | 0.43 | 1.51E-01 | |  | | EPN3 | -1.08 | 6.54E-13 | | -0.11 | | 8.20E-01 | -0.38 | 2.48E-01 | |
| ARIH1 | 0.53 | | 3.17E-04 | -0.01 | 9.85E-01 | | 0.11 | 8.47E-01 | |  | | APCDD1L | -1.08 | 5.11E-05 | | 0.11 | | 8.89E-01 | -0.19 | 7.87E-01 | |
| GCC1 | 0.53 | | 1.90E-03 | -0.18 | 6.36E-01 | | -0.05 | 9.31E-01 | |  | | CBX6 | -1.08 | 3.99E-06 | | 0.42 | | 2.93E-01 | 0.46 | 2.59E-01 | |
| ZFX | 0.53 | | 2.17E-04 | -0.30 | 2.71E-01 | | -0.26 | 4.58E-01 | |  | | CLDN3 | -1.09 | 9.36E-03 | | -0.58 | | 3.64E-01 | -0.70 | 3.01E-01 | |
| ZFP91 | 0.53 | | 5.47E-03 | 0.00 | 9.98E-01 | | -0.03 | 9.69E-01 | |  | | CTSF | -1.10 | 5.18E-03 | | -0.28 | | 7.21E-01 | -0.45 | 4.65E-01 | |
| EBAG9 | 0.53 | | 1.88E-03 | 0.12 | 8.17E-01 | | 0.05 | 9.32E-01 | |  | | FAM111B | -1.10 | 5.61E-14 | | -0.07 | | 8.76E-01 | -0.10 | 8.57E-01 | |
| ZNF330 | 0.53 | | 3.60E-03 | 0.31 | 2.12E-01 | | 0.17 | 6.85E-01 | |  | | C19orf57 | -1.11 | 9.94E-03 | | 0.02 | | 9.87E-01 | -0.24 | 7.86E-01 | |
| AFF4 | 0.53 | | 2.04E-03 | 0.18 | 6.69E-01 | | 0.29 | 4.52E-01 | |  | | CCDC167 | -1.12 | 1.32E-04 | | 0.16 | | 8.38E-01 | -0.42 | 4.91E-01 | |
| RASSF1 | 0.53 | | 3.09E-04 | -0.19 | 6.24E-01 | | -0.24 | 5.46E-01 | |  | | PFKFB4 | -1.13 | 3.70E-03 | | -0.23 | | 7.83E-01 | -0.32 | 6.59E-01 | |
| EP300 | 0.53 | | 3.06E-03 | 0.03 | 9.59E-01 | | 0.12 | 7.90E-01 | |  | | EPPK1 | -1.13 | 5.06E-12 | | -0.44 | | 8.69E-02 | -0.14 | 8.00E-01 | |
| RAP2B | 0.53 | | 4.49E-04 | -0.36 | 1.22E-01 | | -0.01 | 9.91E-01 | |  | | TMEM168 | -1.14 | 7.24E-08 | | -0.40 | | 4.32E-01 | -0.59 | 1.62E-01 | |
| CHD1 | 0.53 | | 1.95E-03 | 0.08 | 8.99E-01 | | 0.10 | 8.69E-01 | |  | | HIST1H1B | -1.16 | 3.35E-05 | | 0.07 | | 9.44E-01 | -0.12 | 8.71E-01 | |
| USP31 | 0.53 | | 9.71E-04 | 0.04 | 9.43E-01 | | 0.15 | 7.09E-01 | |  | | ADGRA3 | -1.16 | 6.63E-09 | | -0.19 | | 7.37E-01 | -0.41 | 2.34E-01 | |
| NOP58 | 0.52 | | 2.83E-04 | 0.05 | 9.29E-01 | | 0.03 | 9.60E-01 | |  | | HIST1H3G | -1.17 | 1.56E-04 | | -0.17 | | 8.12E-01 | -0.38 | 4.92E-01 | |
| SOS1 | 0.52 | | 1.69E-03 | -0.27 | 5.16E-01 | | 0.01 | 9.86E-01 | |  | | ABCD3 | -1.18 | 5.19E-16 | | -0.30 | | 3.84E-01 | -0.47 | 2.01E-01 | |
| KIF2A | 0.52 | | 4.27E-03 | 0.10 | 8.61E-01 | | 0.12 | 8.50E-01 | |  | | PLAT | -1.18 | 2.10E-04 | | -0.45 | | 3.49E-01 | -0.63 | 1.18E-01 | |
| TNFAIP8 | 0.52 | | 3.90E-03 | -0.43 | 7.54E-02 | | -0.17 | 7.50E-01 | |  | | SLC39A10 | -1.19 | 2.13E-08 | | 0.49 | | 5.16E-02 | 0.32 | 4.85E-01 | |
| RBM18 | 0.52 | | 1.43E-04 | 0.24 | 4.16E-01 | | 0.16 | 7.40E-01 | |  | | TRIM45 | -1.19 | 1.50E-03 | | -0.42 | | 5.01E-01 | -0.53 | 3.76E-01 | |
| ARHGEF28 | 0.52 | | 7.90E-03 | 0.25 | 4.84E-01 | | 0.39 | 2.73E-01 | |  | | BMP4 | -1.21 | 5.86E-15 | | -0.11 | | 8.23E-01 | -0.17 | 7.50E-01 | |
| ABITRAM | 0.52 | | 3.64E-04 | -0.16 | 7.77E-01 | | -0.14 | 7.92E-01 | |  | | HSPE1 | -1.22 | 2.71E-10 | | 0.09 | | 8.58E-01 | 0.03 | 9.70E-01 | |
| C5orf51 | 0.52 | | 6.38E-03 | -0.13 | 8.24E-01 | | -0.21 | 7.70E-01 | |  | | ADAM19 | -1.24 | 2.25E-14 | | 0.70 | | 5.58E-05 | 0.18 | 6.45E-01 | |
| ZNF770 | 0.52 | | 5.61E-05 | 0.06 | 9.21E-01 | | -0.15 | 7.70E-01 | |  | | WNT7B | -1.25 | 2.19E-08 | | -0.20 | | 7.00E-01 | -0.42 | 3.32E-01 | |
| HR | 0.52 | | 1.24E-04 | -0.02 | 9.76E-01 | | 0.34 | 3.58E-01 | |  | | MEGF8 | -1.25 | 1.22E-03 | | -0.11 | | 9.29E-01 | -0.04 | 9.67E-01 | |
| KBTBD2 | 0.52 | | 1.65E-03 | -0.10 | 8.63E-01 | | -0.02 | 9.75E-01 | |  | | CISH | -1.26 | 4.88E-06 | | 0.08 | | 9.27E-01 | 0.01 | 9.92E-01 | |
| SLC25A36 | 0.52 | | 1.41E-04 | -0.08 | 8.98E-01 | | -0.11 | 8.44E-01 | |  | | LZTS3 | -1.26 | 6.18E-03 | | -0.34 | | 6.72E-01 | -0.41 | 6.09E-01 | |
| ELF4 | 0.52 | | 7.22E-03 | -0.05 | 9.39E-01 | | 0.06 | 9.28E-01 | |  | | TFRC | -1.27 | 3.33E-13 | | -0.49 | | 1.02E-01 | -0.46 | 2.21E-01 | |
| PPM1B | 0.52 | | 1.47E-03 | -0.35 | 2.72E-01 | | -0.45 | 1.99E-01 | |  | | TMPRSS13 | -1.28 | 4.98E-04 | | -0.32 | | 6.27E-01 | -0.38 | 5.26E-01 | |
| S100PBP | 0.52 | | 4.94E-03 | -0.18 | 7.52E-01 | | -0.17 | 7.51E-01 | |  | | PCDHGB4 | -1.28 | 3.07E-05 | | -0.47 | | 3.01E-01 | -0.56 | 2.04E-01 | |
| SLC39A4 | 0.52 | | 3.15E-03 | 0.48 | 1.49E-01 | | 0.51 | 2.08E-01 | |  | | PHTF1 | -1.29 | 1.92E-13 | | -0.32 | | 3.62E-01 | -0.16 | 7.44E-01 | |
| STAT3 | 0.52 | | 9.16E-05 | -0.21 | 4.25E-01 | | 0.21 | 5.35E-01 | |  | | HEXIM1 | -1.29 | 2.08E-09 | | -0.06 | | 9.33E-01 | 0.03 | 9.62E-01 | |
| BFAR | 0.52 | | 5.94E-04 | -0.04 | 9.45E-01 | | -0.09 | 8.59E-01 | |  | | CNTN1 | -1.30 | 5.80E-18 | | 0.33 | | 3.38E-01 | -0.04 | 9.51E-01 | |
| ZNF496 | 0.48 | | 1.34E-03 | 0.14 | 7.77E-01 | | 0.20 | 6.55E-01 | |  | | SKP2 | -1.31 | 3.27E-17 | | -0.33 | | 3.72E-01 | -0.40 | 2.42E-01 | |
| CBLL1 | 0.48 | | 1.47E-03 | -0.10 | 8.11E-01 | | -0.09 | 8.73E-01 | |  | | ASIC2 | -1.32 | 6.93E-03 | | -0.01 | | 9.97E-01 | -0.86 | 1.32E-01 | |
| N4BP1 | 0.48 | | 2.06E-03 | -0.28 | 3.29E-01 | | -0.27 | 4.65E-01 | |  | | ZMAT3 | -1.33 | 2.48E-08 | | 0.19 | | 7.13E-01 | -0.18 | 7.63E-01 | |
| PIK3C3 | 0.48 | | 3.96E-03 | 0.05 | 9.44E-01 | | 0.23 | 6.57E-01 | |  | | HSPG2 | -1.33 | 5.47E-10 | | 0.12 | | 8.37E-01 | -0.03 | 9.69E-01 | |
| CNBP | 0.48 | | 4.06E-05 | 0.08 | 8.20E-01 | | 0.04 | 9.41E-01 | |  | | UQCRHL | -1.36 | 2.10E-03 | | 0.03 | | 9.81E-01 | -0.05 | 9.61E-01 | |
| SIK3 | 0.48 | | 5.96E-03 | -0.07 | 8.96E-01 | | 0.17 | 6.89E-01 | |  | | DGCR5 | -1.37 | 2.37E-03 | | -0.27 | | 7.73E-01 | -0.83 | 1.50E-01 | |
| RNF24 | 0.48 | | 1.29E-03 | 0.18 | 5.85E-01 | | 0.21 | 5.65E-01 | |  | | SNHG4 | -1.38 | 3.90E-04 | | -0.11 | | 9.20E-01 | -0.55 | 3.13E-01 | |
| SLC7A5 | 0.48 | | 5.95E-03 | -0.13 | 7.57E-01 | | -0.05 | 9.39E-01 | |  | | IL17RE | -1.39 | 5.20E-03 | | -0.01 | | 9.93E-01 | -0.21 | 8.21E-01 | |
| USP1 | 0.48 | | 4.19E-04 | -0.13 | 7.62E-01 | | -0.20 | 7.25E-01 | |  | | CLDN4 | -1.39 | 2.83E-26 | | -0.01 | | 9.93E-01 | -0.02 | 9.79E-01 | |
| FBXL5 | 0.48 | | 2.79E-03 | -0.37 | 1.74E-01 | | -0.12 | 8.23E-01 | |  | | KCNQ1OT1 | -1.40 | 7.24E-06 | | -0.16 | | 8.29E-01 | -0.29 | 6.47E-01 | |
| PSPH | 0.48 | | 1.14E-03 | -0.04 | 9.49E-01 | | -0.09 | 8.51E-01 | |  | | SV2A | -1.40 | 5.69E-03 | | -0.41 | | 5.17E-01 | -0.68 | 2.54E-01 | |
| DTX2 | 0.48 | | 1.90E-03 | -0.13 | 7.81E-01 | | 0.18 | 7.15E-01 | |  | | KRT87P | -1.41 | 1.43E-04 | | -0.74 | | 8.77E-02 | -0.76 | 1.00E-01 | |
| CNOT10 | 0.48 | | 8.53E-03 | -0.21 | 6.28E-01 | | -0.10 | 8.43E-01 | |  | | CCL26 | -1.41 | 7.31E-03 | | -0.09 | | 9.47E-01 | -0.77 | 2.06E-01 | |
| SIN3A | 0.48 | | 1.29E-03 | -0.16 | 6.66E-01 | | -0.06 | 9.00E-01 | |  | | CHST12 | -1.41 | 8.08E-10 | | 0.18 | | 7.35E-01 | 0.03 | 9.68E-01 | |
| BCL2L13 | 0.48 | | 3.93E-04 | -0.24 | 3.45E-01 | | -0.05 | 9.25E-01 | |  | | SYNDIG1 | -1.44 | 1.02E-03 | | 0.28 | | 6.95E-01 | -0.41 | 5.28E-01 | |
| MTIF3 | 0.48 | | 8.06E-03 | 0.24 | 5.09E-01 | | 0.04 | 9.52E-01 | |  | | CASTOR3 | -1.44 | 3.15E-04 | | -0.49 | | 4.10E-01 | -0.74 | 1.91E-01 | |
| TBRG1 | 0.48 | | 1.91E-03 | 0.22 | 4.75E-01 | | 0.31 | 3.27E-01 | |  | | CCNE2 | -1.45 | 2.61E-11 | | -0.23 | | 6.49E-01 | -0.35 | 4.81E-01 | |
| RNF111 | 0.47 | | 4.83E-03 | -0.04 | 9.59E-01 | | 0.10 | 8.82E-01 | |  | | B4GAT1 | -1.46 | 8.57E-10 | | -0.29 | | 3.58E-01 | -0.46 | 2.10E-01 | |
| TMEM167B | 0.47 | | 6.83E-03 | -0.04 | 9.55E-01 | | -0.03 | 9.60E-01 | |  | | TNFSF9 | -1.47 | 1.13E-17 | | -0.57 | | 1.24E-02 | -0.37 | 3.26E-01 | |
| BTBD10 | 0.47 | | 7.32E-04 | 0.17 | 7.07E-01 | | 0.38 | 1.90E-01 | |  | | CAVIN2 | -1.48 | 5.28E-04 | | -0.06 | | 9.60E-01 | -0.76 | 1.33E-01 | |
| MLH3 | 0.47 | | 9.44E-03 | 0.27 | 4.84E-01 | | 0.37 | 3.25E-01 | |  | | IL1R1 | -1.48 | 2.31E-11 | | -0.40 | | 3.55E-01 | -0.49 | 1.68E-01 | |
| USP12 | 0.47 | | 6.32E-03 | 0.05 | 9.45E-01 | | -0.01 | 9.90E-01 | |  | | PLA2G7 | -1.53 | 4.87E-04 | | 1.21 | | 8.10E-05 | 0.22 | 7.82E-01 | |
| ABRAXAS2 | 0.47 | | 2.17E-03 | 0.06 | 9.18E-01 | | -0.02 | 9.68E-01 | |  | | NT5DC2 | -1.54 | 9.45E-20 | | -0.22 | | 5.61E-01 | -0.51 | 1.99E-01 | |
| FNTA | 0.47 | | 1.17E-03 | 0.03 | 9.63E-01 | | -0.15 | 7.72E-01 | |  | | GPER1 | -1.55 | 9.30E-03 | | -1.09 | | 1.24E-01 | -0.93 | 2.22E-01 | |
| SDE2 | 0.47 | | 3.47E-03 | -0.07 | 9.17E-01 | | 0.09 | 8.72E-01 | |  | | HPDL | -1.58 | 2.50E-10 | | -0.23 | | 6.63E-01 | -0.17 | 8.05E-01 | |
| EIF2AK2 | 0.47 | | 7.59E-04 | -0.16 | 6.95E-01 | | 0.46 | 2.77E-01 | |  | | MAP1A | -1.60 | 2.05E-07 | | -0.65 | | 8.39E-02 | -0.67 | 1.14E-01 | |
| GOLGB1 | 0.47 | | 3.80E-03 | -0.01 | 9.86E-01 | | -0.23 | 5.17E-01 | |  | | LRG1 | -1.60 | 4.52E-08 | | 0.26 | | 6.77E-01 | 0.37 | 4.77E-01 | |
| RBM26 | 0.47 | | 5.40E-03 | 0.32 | 2.83E-01 | | 0.37 | 3.31E-01 | |  | | GVQW3 | -1.75 | 1.97E-03 | | -0.51 | | 5.96E-01 | -0.48 | 5.85E-01 | |
| POGK | 0.47 | | 4.03E-03 | -0.12 | 7.89E-01 | | -0.04 | 9.40E-01 | |  | | MAGEE1 | -1.76 | 6.61E-06 | | -0.19 | | 8.06E-01 | 0.13 | 8.62E-01 | |
| SOD2 | 0.47 | | 7.54E-04 | -0.36 | 1.27E-01 | | 0.14 | 7.48E-01 | |  | | TMPRSS11E | -1.81 | 3.35E-04 | | 0.32 | | 6.87E-01 | -0.15 | 8.82E-01 | |
| NQO1 | 0.47 | | 1.25E-03 | -0.27 | 2.46E-01 | | 0.04 | 9.43E-01 | |  | | JAKMIP3 | -1.84 | 2.98E-03 | | -0.61 | | 4.86E-01 | -0.65 | 4.85E-01 | |
| NOB1 | 0.46 | | 1.58E-03 | 0.23 | 5.22E-01 | | 0.26 | 4.79E-01 | |  | | CDON | -1.88 | 3.28E-03 | | 0.15 | | 9.19E-01 | -0.31 | 7.69E-01 | |
| KHNYN | 0.46 | | 5.18E-03 | 0.14 | 7.10E-01 | | 0.33 | 2.34E-01 | |  | | MISP3 | -1.89 | 2.06E-03 | | -0.83 | | 2.92E-01 | -1.03 | 1.60E-01 | |
| DSG3 | 0.46 | | 5.21E-03 | -0.45 | 4.94E-02 | | -0.26 | 4.81E-01 | |  | | MATR3 | -1.91 | 1.85E-03 | | -0.63 | | 4.61E-01 | -0.94 | 1.92E-01 | |
| CDC42SE1 | 0.46 | | 5.08E-03 | 0.19 | 5.81E-01 | | 0.20 | 6.07E-01 | |  | | UPK2 | -1.96 | 8.28E-19 | | 0.52 | | 7.45E-02 | 0.03 | 9.62E-01 | |
| TCEA1 | 0.46 | | 5.78E-03 | 0.13 | 8.06E-01 | | 0.05 | 9.46E-01 | |  | | TCF7 | -2.09 | 3.24E-03 | | -0.40 | | 7.66E-01 | -1.16 | 1.72E-01 | |
| RSF1 | 0.46 | | 6.35E-03 | 0.04 | 9.45E-01 | | 0.00 | 9.98E-01 | |  | | LCE1E | -2.24 | 2.27E-04 | | 0.56 | | 4.41E-01 | 0.42 | 5.87E-01 | |
| RBM22 | 0.46 | | 6.87E-04 | 0.19 | 4.82E-01 | | 0.24 | 4.45E-01 | |  | | MIR600HG | -2.28 | 8.28E-03 | | -0.26 | | 8.75E-01 | -1.33 | 1.54E-01 | |
| AKIP1 | 0.46 | | 6.11E-04 | -0.01 | 9.83E-01 | | 0.06 | 9.02E-01 | |  | | METTL7B | -2.36 | 9.46E-03 | | -0.32 | | 8.55E-01 | -1.00 | 3.32E-01 | |
| TMEM192 | 0.46 | | 7.22E-03 | 0.38 | 1.10E-01 | | 0.19 | 6.99E-01 | |  | | GAS6-AS1 | -2.54 | 7.76E-04 | | -0.12 | | 9.47E-01 | -1.30 | 1.22E-01 | |
| TRIM47 | 0.46 | | 8.05E-04 | 0.00 | 9.97E-01 | | 0.19 | 6.59E-01 | |  | | PLCXD3 | -2.56 | 6.66E-03 | | -1.79 | | 7.33E-02 | -1.29 | 2.00E-01 | |
| ITPK1 | 0.46 | | 4.95E-04 | 0.16 | 6.23E-01 | | 0.44 | 1.83E-01 | |  | | HSPA8 | -2.59 | 1.72E-68 | | -0.10 | | 9.04E-01 | 0.13 | 7.91E-01 | |
| FSTL3 | 0.46 | | 6.50E-04 | -0.19 | 6.20E-01 | | -0.17 | 7.28E-01 | |  | | POU3F1 | -2.62 | 3.81E-05 | | -0.05 | | 9.75E-01 | -0.61 | 4.86E-01 | |
| NOL8 | 0.46 | | 1.18E-03 | 0.11 | 7.72E-01 | | -0.05 | 9.31E-01 | |  | | HSPA1B | -2.85 | 8.95E-51 | | 0.03 | | 9.64E-01 | 0.13 | 8.18E-01 | |
| GPBP1L1 | 0.46 | | 5.61E-04 | 0.09 | 8.40E-01 | | 0.23 | 5.25E-01 | |  | | S100A7 | -3.02 | 4.97E-05 | | 0.20 | | 8.72E-01 | -1.10 | 1.59E-01 | |
| DNAJA3 | 0.45 | | 8.67E-04 | -0.05 | 9.20E-01 | | 0.06 | 8.97E-01 | |  | | HSPA1A | -3.51 | 1.61E-65 | | 0.10 | | 8.38E-01 | 0.14 | 7.50E-01 | |
| CTTNBP2NL | 0.45 | | 8.27E-03 | -0.04 | 9.60E-01 | | 0.09 | 8.90E-01 | |  | | ALPK3 | -3.60 | 1.38E-08 | | -0.69 | | 2.38E-01 | -0.80 | 1.42E-01 | |
| **Supplementary Table 3.** IFN-γ induced genes significantly modified by EGF-treatment (adjusted *P_interaction_* < 0.05) | | | | | | | | | | | | | | | | | | | | | |
| **ENSGENE** | | **Gene** | | | | **l2fold_EGFvsneith** | | | **padj_1** | | **l2fold_IFNgammavsneither** | | | | **padj_3** | | **l2fold_EGF_IFN_interaction** | | | | **padj_5** |
| ENSG00000169245 | | CXCL10 | | | | 0.42 | | | 8.47E-01 | | 10.52 | | | | 7.24E-118 | | -2.24 | | | | 1.06E-02 |
| ENSG00000154451 | | GBP5 | | | | 0.81 | | | 4.29E-01 | | 9.13 | | | | 1.65E-118 | | -2.27 | | | | 6.63E-04 |
| ENSG00000204257 | | HLA-DMA | | | | 0.17 | | | 8.97E-01 | | 5.73 | | | | 5.49E-114 | | -1.49 | | | | 1.42E-03 |
| ENSG00000130513 | | GDF15 | | | | 1.28 | | | 1.24E-01 | | 4.17 | | | | 3.52E-16 | | -5.53 | | | | 1.26E-11 |
| ENSG00000139269 | | INHBE | | | | -0.40 | | | 9.28E-01 | | 4.13 | | | | 1.58E-07 | | -5.73 | | | | 3.86E-03 |
| ENSG00000258581 | | RP11-638I2.10 | | | | 0.92 | | | 5.88E-01 | | 4.11 | | | | 2.12E-11 | | -2.35 | | | | 4.04E-02 |
| ENSG00000128165 | | ADM2 | | | | 0.93 | | | 1.07E-01 | | 3.88 | | | | 7.40E-30 | | -4.17 | | | | 3.01E-15 |
| ENSG00000128965 | | CHAC1 | | | | 0.00 | | | 9.96E-01 | | 3.83 | | | | 1.08E-107 | | -3.33 | | | | 2.56E-36 |
| ENSG00000265972 | | TXNIP | | | | -0.16 | | | 8.45E-01 | | 3.77 | | | | 2.76E-73 | | -2.23 | | | | 9.78E-12 |
| ENSG00000136244 | | IL6 | | | | -0.40 | | | 6.00E-01 | | 3.60 | | | | 1.76E-40 | | -2.54 | | | | 2.27E-08 |
| ENSG00000115008 | | IL1A | | | | 1.60 | | | 1.15E-10 | | 3.57 | | | | 4.88E-57 | | -3.38 | | | | 2.56E-25 |
| ENSG00000101280 | | ANGPT4 | | | | 1.40 | | | 9.60E-02 | | 3.39 | | | | 7.78E-10 | | -3.89 | | | | 7.33E-06 |
| ENSG00000116852 | | KIF21B | | | | 1.68 | | | 1.16E-02 | | 3.37 | | | | 4.06E-11 | | -3.95 | | | | 3.86E-07 |
| ENSG00000020181 | | ADGRA2 | | | | 0.62 | | | 8.41E-01 | | 3.25 | | | | 5.47E-05 | | -3.38 | | | | 2.69E-02 |
| ENSG00000162772 | | ATF3 | | | | -0.71 | | | 2.73E-01 | | 3.22 | | | | 2.59E-21 | | -2.19 | | | | 3.24E-04 |
| ENSG00000111981 | | ULBP1 | | | | -0.50 | | | 7.27E-01 | | 3.06 | | | | 2.97E-14 | | -3.40 | | | | 1.43E-05 |
| ENSG00000130766 | | SESN2 | | | | -0.06 | | | 9.60E-01 | | 3.02 | | | | 1.54E-41 | | -2.70 | | | | 3.01E-15 |
| ENSG00000113739 | | STC2 | | | | 0.48 | | | 2.51E-02 | | 2.87 | | | | 6.51E-81 | | -2.96 | | | | 1.79E-40 |
| ENSG00000133101 | | CCNA1 | | | | 1.14 | | | 1.49E-01 | | 2.72 | | | | 6.12E-08 | | -2.29 | | | | 9.69E-03 |
| ENSG00000106823 | | ECM2 | | | | -0.80 | | | 1.34E-01 | | 2.66 | | | | 2.45E-17 | | -3.15 | | | | 5.67E-09 |
| ENSG00000112715 | | VEGFA | | | | 1.35 | | | 4.89E-16 | | 2.62 | | | | 4.48E-63 | | -2.42 | | | | 1.08E-25 |
| ENSG00000168209 | | DDIT4 | | | | 0.17 | | | 8.45E-01 | | 2.49 | | | | 5.97E-29 | | -2.51 | | | | 1.61E-13 |
| ENSG00000176046 | | NUPR1 | | | | -3.13 | | | 5.86E-05 | | 2.42 | | | | 9.02E-10 | | -3.45 | | | | 3.61E-02 |
| ENSG00000116761 | | CTH | | | | -0.36 | | | 2.82E-01 | | 2.32 | | | | 1.13E-39 | | -1.81 | | | | 1.46E-10 |
| ENSG00000175197 | | DDIT3 | | | | 0.08 | | | 9.38E-01 | | 2.26 | | | | 1.66E-29 | | -2.42 | | | | 2.87E-15 |
| ENSG00000070669 | | ASNS | | | | -0.19 | | | 7.31E-01 | | 2.22 | | | | 7.16E-37 | | -2.26 | | | | 1.21E-17 |
| ENSG00000101255 | | TRIB3 | | | | 0.03 | | | 9.80E-01 | | 2.13 | | | | 4.13E-21 | | -2.06 | | | | 5.67E-09 |
| ENSG00000265778 | | RP11-17M16.2 | | | | 0.51 | | | 6.96E-01 | | 2.11 | | | | 3.83E-06 | | -1.84 | | | | 3.38E-02 |
| ENSG00000171658 | | NMRAL1P1 | | | | -0.13 | | | 8.80E-01 | | 2.11 | | | | 4.62E-26 | | -1.52 | | | | 6.82E-06 |
| ENSG00000116717 | | GADD45A | | | | -0.17 | | | 8.24E-01 | | 2.05 | | | | 4.89E-24 | | -1.85 | | | | 6.86E-09 |
| ENSG00000172216 | | CEBPB | | | | 0.26 | | | 6.68E-01 | | 2.03 | | | | 1.36E-19 | | -1.68 | | | | 4.69E-06 |
| ENSG00000087074 | | PPP1R15A | | | | 0.29 | | | 6.76E-01 | | 1.97 | | | | 1.94E-14 | | -1.66 | | | | 1.19E-04 |
| ENSG00000157514 | | TSC22D3 | | | | -0.94 | | | 2.49E-08 | | 1.94 | | | | 3.06E-40 | | -2.22 | | | | 1.28E-22 |
| ENSG00000151012 | | SLC7A11 | | | | -0.14 | | | 8.87E-01 | | 1.94 | | | | 2.58E-16 | | -2.08 | | | | 2.62E-08 |
| ENSG00000124613 | | ZNF391 | | | | 0.31 | | | 8.23E-01 | | 1.93 | | | | 2.40E-07 | | -2.21 | | | | 6.43E-04 |
| ENSG00000163297 | | ANTXR2 | | | | 2.97 | | | 2.32E-51 | | 1.93 | | | | 1.47E-21 | | -1.43 | | | | 1.07E-05 |
| ENSG00000100889 | | PCK2 | | | | -0.03 | | | 9.79E-01 | | 1.91 | | | | 4.58E-22 | | -1.77 | | | | 1.17E-08 |
| ENSG00000006459 | | KDM7A | | | | 0.47 | | | 4.00E-01 | | 1.89 | | | | 4.56E-12 | | -1.62 | | | | 6.05E-04 |
| ENSG00000140450 | | ARRDC4 | | | | -0.50 | | | 2.50E-01 | | 1.88 | | | | 1.40E-14 | | -1.21 | | | | 8.89E-03 |
| ENSG00000197863 | | ZNF790 | | | | 0.67 | | | 5.80E-01 | | 1.85 | | | | 4.53E-04 | | -2.35 | | | | 1.21E-02 |
| ENSG00000065809 | | FAM107B | | | | 0.42 | | | 2.45E-01 | | 1.81 | | | | 2.86E-18 | | -2.02 | | | | 2.28E-10 |
| ENSG00000186832 | | KRT16 | | | | -0.86 | | | 6.61E-04 | | 1.78 | | | | 2.42E-17 | | -0.89 | | | | 3.74E-02 |
| ENSG00000146592 | | CREB5 | | | | 0.11 | | | 9.77E-01 | | 1.76 | | | | 1.61E-02 | | -2.83 | | | | 3.74E-02 |
| ENSG00000019549 | | SNAI2 | | | | -0.18 | | | 7.49E-01 | | 1.71 | | | | 5.14E-22 | | -1.81 | | | | 2.86E-11 |
| ENSG00000181894 | | ZNF329 | | | | 0.31 | | | 8.41E-01 | | 1.68 | | | | 8.24E-05 | | -1.63 | | | | 4.47E-02 |
| ENSG00000116285 | | ERRFI1 | | | | 0.01 | | | 9.93E-01 | | 1.67 | | | | 4.43E-13 | | -1.64 | | | | 1.09E-05 |
| ENSG00000157168 | | NRG1 | | | | -0.06 | | | 9.26E-01 | | 1.65 | | | | 4.64E-39 | | -1.42 | | | | 2.11E-13 |
| ENSG00000196517 | | SLC6A9 | | | | -0.41 | | | 8.28E-02 | | 1.65 | | | | 9.77E-27 | | -1.31 | | | | 1.60E-07 |
| ENSG00000172059 | | KLF11 | | | | 0.12 | | | 9.32E-01 | | 1.63 | | | | 8.85E-08 | | -1.55 | | | | 3.88E-03 |
| ENSG00000102804 | | TSC22D1 | | | | 0.38 | | | 2.59E-01 | | 1.62 | | | | 3.87E-17 | | -1.60 | | | | 1.26E-07 |
| ENSG00000132846 | | ZBED3 | | | | -0.22 | | | 8.44E-01 | | 1.62 | | | | 2.54E-08 | | -1.41 | | | | 1.17E-02 |
| ENSG00000243710 | | CFAP57 | | | | -0.09 | | | 9.47E-01 | | 1.61 | | | | 6.43E-12 | | -1.02 | | | | 3.11E-02 |
| ENSG00000179630 | | LACC1 | | | | 0.42 | | | 5.70E-01 | | 1.59 | | | | 7.14E-07 | | -1.43 | | | | 1.47E-02 |
| ENSG00000270882 | | HIST2H4A | | | | 0.23 | | | 8.66E-01 | | 1.58 | | | | 3.62E-06 | | -1.42 | | | | 3.12E-02 |
| ENSG00000204524 | | ZNF805 | | | | 0.02 | | | 9.88E-01 | | 1.57 | | | | 4.94E-06 | | -1.36 | | | | 3.68E-02 |
| ENSG00000133639 | | BTG1 | | | | 0.07 | | | 9.28E-01 | | 1.57 | | | | 2.30E-21 | | -1.21 | | | | 1.03E-05 |
| ENSG00000074935 | | TUBE1 | | | | 0.15 | | | 8.55E-01 | | 1.54 | | | | 1.88E-13 | | -1.76 | | | | 8.75E-08 |
| ENSG00000059728 | | MXD1 | | | | 0.24 | | | 7.60E-01 | | 1.50 | | | | 1.61E-09 | | -1.31 | | | | 2.88E-03 |
| ENSG00000115602 | | IL1RL1 | | | | 3.59 | | | 2.88E-63 | | 1.49 | | | | 9.10E-11 | | -1.78 | | | | 1.61E-07 |
| ENSG00000174749 | | C4orf32 | | | | 0.52 | | | 3.59E-01 | | 1.49 | | | | 1.78E-06 | | -1.58 | | | | 3.06E-03 |
| ENSG00000115963 | | RND3 | | | | -0.37 | | | 3.08E-01 | | 1.49 | | | | 9.03E-14 | | -1.44 | | | | 9.81E-06 |
| ENSG00000155158 | | TTC39B | | | | 0.20 | | | 8.41E-01 | | 1.47 | | | | 2.29E-08 | | -1.29 | | | | 7.03E-03 |
| ENSG00000135069 | | PSAT1 | | | | -0.26 | | | 4.52E-01 | | 1.43 | | | | 3.84E-18 | | -1.50 | | | | 3.69E-09 |
| ENSG00000198431 | | TXNRD1 | | | | -0.67 | | | 8.18E-03 | | 1.43 | | | | 2.63E-12 | | -0.98 | | | | 9.29E-03 |
| ENSG00000205189 | | ZBTB10 | | | | -0.14 | | | 9.64E-01 | | 1.41 | | | | 1.72E-02 | | -2.34 | | | | 3.17E-02 |
| ENSG00000023608 | | SNAPC1 | | | | 0.37 | | | 4.89E-01 | | 1.41 | | | | 1.91E-08 | | -1.51 | | | | 2.88E-04 |
| ENSG00000197044 | | ZNF441 | | | | -0.03 | | | 9.88E-01 | | 1.39 | | | | 4.61E-05 | | -1.54 | | | | 1.39E-02 |
| ENSG00000140830 | | TXNL4B | | | | 0.04 | | | 9.64E-01 | | 1.39 | | | | 4.09E-18 | | -1.28 | | | | 7.73E-07 |
| ENSG00000115902 | | SLC1A4 | | | | -0.18 | | | 6.20E-01 | | 1.39 | | | | 4.55E-22 | | -1.25 | | | | 4.54E-08 |
| ENSG00000278970 | | HEIH | | | | 0.02 | | | 9.81E-01 | | 1.38 | | | | 2.70E-13 | | -1.26 | | | | 9.48E-05 |
| ENSG00000119986 | | AVPI1 | | | | 0.11 | | | 9.09E-01 | | 1.36 | | | | 1.80E-09 | | -1.42 | | | | 1.17E-04 |
| ENSG00000177426 | | TGIF1 | | | | 0.11 | | | 8.59E-01 | | 1.36 | | | | 8.80E-19 | | -1.27 | | | | 2.41E-07 |
| ENSG00000117525 | | F3 | | | | -0.09 | | | 9.15E-01 | | 1.36 | | | | 1.27E-13 | | -1.13 | | | | 2.88E-04 |
| ENSG00000166123 | | GPT2 | | | | -0.05 | | | 9.57E-01 | | 1.35 | | | | 5.75E-15 | | -1.30 | | | | 3.13E-06 |
| ENSG00000131711 | | MAP1B | | | | -0.04 | | | 9.74E-01 | | 1.34 | | | | 4.60E-10 | | -1.49 | | | | 3.07E-05 |
| ENSG00000065911 | | MTHFD2 | | | | -0.04 | | | 9.69E-01 | | 1.33 | | | | 2.21E-12 | | -1.19 | | | | 1.56E-04 |
| ENSG00000253616 | | RP11-875O11.3 | | | | 0.41 | | | 7.02E-01 | | 1.32 | | | | 1.21E-03 | | -1.62 | | | | 2.59E-02 |
| ENSG00000255717 | | SNHG1 | | | | 0.18 | | | 7.22E-01 | | 1.31 | | | | 7.05E-14 | | -1.40 | | | | 3.39E-07 |
| ENSG00000134294 | | SLC38A2 | | | | -0.12 | | | 9.32E-01 | | 1.31 | | | | 2.98E-05 | | -1.19 | | | | 3.90E-02 |
| ENSG00000153879 | | CEBPG | | | | -0.09 | | | 8.65E-01 | | 1.30 | | | | 1.01E-21 | | -1.35 | | | | 1.24E-10 |
| ENSG00000083817 | | ZNF416 | | | | 0.36 | | | 6.41E-01 | | 1.29 | | | | 4.05E-05 | | -1.54 | | | | 4.34E-03 |
| ENSG00000122644 | | ARL4A | | | | -0.12 | | | 8.73E-01 | | 1.29 | | | | 2.49E-12 | | -1.26 | | | | 3.57E-05 |
| ENSG00000151773 | | CCDC122 | | | | 0.34 | | | 7.54E-01 | | 1.27 | | | | 7.93E-04 | | -1.57 | | | | 1.87E-02 |
| ENSG00000141448 | | GATA6 | | | | 0.32 | | | 4.32E-01 | | 1.27 | | | | 3.41E-10 | | -1.37 | | | | 3.14E-05 |
| ENSG00000183309 | | ZNF623 | | | | -0.26 | | | 7.13E-01 | | 1.27 | | | | 5.18E-07 | | -1.17 | | | | 1.00E-02 |
| ENSG00000140961 | | OSGIN1 | | | | -0.28 | | | 5.49E-01 | | 1.27 | | | | 2.53E-10 | | -1.06 | | | | 3.55E-03 |
| ENSG00000100219 | | XBP1 | | | | -0.12 | | | 7.14E-01 | | 1.26 | | | | 1.93E-30 | | -1.32 | | | | 1.80E-15 |
| ENSG00000109618 | | SEPSECS | | | | 0.12 | | | 9.26E-01 | | 1.26 | | | | 1.86E-05 | | -1.17 | | | | 3.37E-02 |
| ENSG00000163993 | | S100P | | | | -2.31 | | | 3.83E-08 | | 1.25 | | | | 1.30E-04 | | -1.83 | | | | 2.67E-02 |
| ENSG00000079156 | | OSBPL6 | | | | 0.68 | | | 3.20E-01 | | 1.25 | | | | 2.54E-03 | | -1.74 | | | | 1.41E-02 |
| ENSG00000129474 | | AJUBA | | | | -0.28 | | | 7.59E-02 | | 1.25 | | | | 1.77E-31 | | -1.14 | | | | 4.79E-12 |
| ENSG00000227375 | | DLG1-AS1 | | | | 0.01 | | | 9.96E-01 | | 1.23 | | | | 1.56E-05 | | -1.71 | | | | 4.63E-04 |
| ENSG00000196387 | | ZNF140 | | | | -0.12 | | | 9.06E-01 | | 1.23 | | | | 1.45E-07 | | -1.02 | | | | 2.04E-02 |
| ENSG00000163393 | | SLC22A15 | | | | -0.18 | | | 8.67E-01 | | 1.19 | | | | 2.19E-05 | | -1.68 | | | | 3.37E-04 |
| ENSG00000139211 | | AMIGO2 | | | | -0.27 | | | 6.55E-01 | | 1.19 | | | | 5.96E-07 | | -1.44 | | | | 1.79E-04 |
| ENSG00000196653 | | ZNF502 | | | | 0.69 | | | 4.11E-01 | | 1.18 | | | | 1.46E-02 | | -1.91 | | | | 1.92E-02 |
| ENSG00000157429 | | ZNF19 | | | | 0.11 | | | 9.48E-01 | | 1.18 | | | | 1.17E-03 | | -1.39 | | | | 3.73E-02 |
| ENSG00000110619 | | CARS | | | | -0.08 | | | 9.03E-01 | | 1.17 | | | | 4.09E-14 | | -1.08 | | | | 1.98E-05 |
| ENSG00000164484 | | TMEM200A | | | | 2.20 | | | 6.14E-22 | | 1.17 | | | | 2.75E-06 | | -0.96 | | | | 2.97E-02 |
| ENSG00000131791 | | PRKAB2 | | | | -0.26 | | | 6.49E-01 | | 1.16 | | | | 1.41E-07 | | -1.24 | | | | 8.42E-04 |
| ENSG00000065060 | | UHRF1BP1 | | | | -0.54 | | | 6.28E-02 | | 1.15 | | | | 2.42E-08 | | -1.10 | | | | 2.47E-03 |
| ENSG00000174564 | | IL20RB | | | | -0.33 | | | 1.64E-01 | | 1.15 | | | | 9.87E-15 | | -1.01 | | | | 3.79E-05 |
| ENSG00000168944 | | CEP120 | | | | 0.14 | | | 8.99E-01 | | 1.14 | | | | 3.53E-05 | | -1.20 | | | | 1.40E-02 |
| ENSG00000178229 | | ZNF543 | | | | -0.01 | | | 9.92E-01 | | 1.13 | | | | 1.59E-06 | | -1.31 | | | | 1.14E-03 |
| ENSG00000106105 | | GARS | | | | -0.12 | | | 8.18E-01 | | 1.13 | | | | 1.04E-14 | | -1.10 | | | | 2.57E-06 |
| ENSG00000090861 | | AARS | | | | -0.24 | | | 4.38E-01 | | 1.12 | | | | 4.03E-13 | | -1.09 | | | | 1.07E-05 |
| ENSG00000251022 | | THAP9-AS1 | | | | -0.05 | | | 9.67E-01 | | 1.11 | | | | 1.87E-06 | | -1.24 | | | | 1.94E-03 |
| ENSG00000198625 | | MDM4 | | | | -0.11 | | | 8.87E-01 | | 1.11 | | | | 7.83E-09 | | -0.96 | | | | 6.69E-03 |
| ENSG00000198455 | | ZXDB | | | | -0.01 | | | 9.91E-01 | | 1.10 | | | | 6.44E-05 | | -1.06 | | | | 3.95E-02 |
| ENSG00000116044 | | NFE2L2 | | | | -0.29 | | | 2.82E-01 | | 1.10 | | | | 1.54E-12 | | -1.04 | | | | 4.56E-05 |
| ENSG00000108375 | | RNF43 | | | | -0.05 | | | 9.69E-01 | | 1.09 | | | | 3.08E-06 | | -1.18 | | | | 3.70E-03 |
| ENSG00000166024 | | R3HCC1L | | | | -0.02 | | | 9.83E-01 | | 1.09 | | | | 9.89E-09 | | -1.05 | | | | 1.42E-03 |
| ENSG00000164284 | | GRPEL2 | | | | -0.27 | | | 7.23E-01 | | 1.08 | | | | 1.08E-04 | | -1.25 | | | | 9.34E-03 |
| ENSG00000185070 | | FLRT2 | | | | -0.71 | | | 5.40E-02 | | 1.07 | | | | 5.26E-05 | | -1.08 | | | | 4.07E-02 |
| ENSG00000143367 | | TUFT1 | | | | -0.32 | | | 2.85E-01 | | 1.07 | | | | 9.96E-10 | | -0.93 | | | | 2.47E-03 |
| ENSG00000109787 | | KLF3 | | | | -0.24 | | | 6.60E-01 | | 1.06 | | | | 1.37E-06 | | -0.85 | | | | 3.96E-02 |
| ENSG00000122861 | | PLAU | | | | 1.50 | | | 3.10E-49 | | 1.05 | | | | 2.86E-24 | | -0.45 | | | | 2.71E-02 |
| ENSG00000074590 | | NUAK1 | | | | -0.99 | | | 2.29E-05 | | 1.04 | | | | 5.55E-07 | | -1.19 | | | | 1.21E-03 |
| ENSG00000128272 | | ATF4 | | | | -0.05 | | | 9.43E-01 | | 1.04 | | | | 2.44E-14 | | -1.01 | | | | 4.05E-06 |
| ENSG00000161920 | | MED11 | | | | 0.00 | | | 9.99E-01 | | -1.00 | | | | 1.40E-04 | | 1.02 | | | | 2.91E-02 |
| ENSG00000125848 | | FLRT3 | | | | 0.30 | | | 5.04E-01 | | -1.02 | | | | 1.17E-05 | | 1.09 | | | | 4.40E-03 |
| ENSG00000186594 | | MIR22HG | | | | 0.09 | | | 9.31E-01 | | -1.07 | | | | 9.28E-06 | | 1.11 | | | | 5.49E-03 |
| ENSG00000183741 | | CBX6 | | | | 0.42 | | | 3.63E-01 | | -1.08 | | | | 4.64E-05 | | 1.12 | | | | 1.65E-02 |
| ENSG00000163739 | | CXCL1 | | | | -0.13 | | | 8.78E-01 | | -1.12 | | | | 4.15E-07 | | 1.84 | | | | 1.74E-08 |
| ENSG00000198624 | | CCDC69 | | | | 0.35 | | | 3.38E-01 | | -1.17 | | | | 5.35E-07 | | 1.48 | | | | 1.17E-05 |
| ENSG00000196950 | | SLC39A10 | | | | 0.49 | | | 1.58E-01 | | -1.19 | | | | 1.07E-06 | | 1.02 | | | | 1.85E-02 |
| ENSG00000125378 | | BMP4 | | | | -0.12 | | | 8.88E-01 | | -1.20 | | | | 1.60E-08 | | 1.14 | | | | 1.45E-03 |
| ENSG00000115541 | | HSPE1 | | | | 0.08 | | | 9.56E-01 | | -1.22 | | | | 6.37E-06 | | 1.17 | | | | 1.61E-02 |
| ENSG00000170477 | | KRT4 | | | | -3.95 | | | 2.26E-112 | | -1.23 | | | | 1.05E-11 | | 0.85 | | | | 1.66E-02 |
| ENSG00000135074 | | ADAM19 | | | | 0.69 | | | 5.87E-06 | | -1.24 | | | | 1.98E-17 | | 0.73 | | | | 5.14E-03 |
| ENSG00000114737 | | CISH | | | | 0.08 | | | 9.48E-01 | | -1.26 | | | | 1.98E-06 | | 1.18 | | | | 7.52E-03 |
| ENSG00000186834 | | HEXIM1 | | | | -0.07 | | | 9.42E-01 | | -1.29 | | | | 2.26E-10 | | 1.39 | | | | 8.07E-06 |
| ENSG00000116793 | | PHTF1 | | | | -0.33 | | | 4.27E-01 | | -1.29 | | | | 3.87E-09 | | 1.45 | | | | 2.42E-05 |
| ENSG00000018236 | | CNTN1 | | | | 0.32 | | | 4.79E-01 | | -1.30 | | | | 2.68E-09 | | 0.94 | | | | 2.25E-02 |
| ENSG00000172667 | | ZMAT3 | | | | 0.19 | | | 8.04E-01 | | -1.33 | | | | 7.33E-08 | | 0.96 | | | | 3.61E-02 |
| ENSG00000142798 | | HSPG2 | | | | 0.12 | | | 9.16E-01 | | -1.33 | | | | 8.68E-07 | | 1.18 | | | | 1.43E-02 |
| ENSG00000269821 | | KCNQ1OT1 | | | | -0.17 | | | 8.82E-01 | | -1.39 | | | | 2.22E-05 | | 1.26 | | | | 3.11E-02 |
| ENSG00000189143 | | CLDN4 | | | | -0.01 | | | 9.90E-01 | | -1.39 | | | | 1.17E-11 | | 1.38 | | | | 3.14E-05 |
| ENSG00000163283 | | ALPP | | | | -2.41 | | | 6.55E-55 | | -1.40 | | | | 2.28E-19 | | 0.90 | | | | 1.54E-03 |
| ENSG00000136213 | | CHST12 | | | | 0.17 | | | 8.48E-01 | | -1.41 | | | | 2.98E-07 | | 1.27 | | | | 7.13E-03 |
| ENSG00000175305 | | CCNE2 | | | | -0.24 | | | 7.88E-01 | | -1.44 | | | | 9.22E-07 | | 1.33 | | | | 9.75E-03 |
| ENSG00000125657 | | TNFSF9 | | | | -0.58 | | | 3.50E-02 | | -1.47 | | | | 4.62E-12 | | 1.67 | | | | 2.93E-07 |
| ENSG00000168268 | | NT5DC2 | | | | -0.23 | | | 7.67E-01 | | -1.54 | | | | 5.16E-10 | | 1.26 | | | | 3.90E-03 |
| ENSG00000186603 | | HPDL | | | | -0.24 | | | 8.28E-01 | | -1.58 | | | | 2.72E-06 | | 1.64 | | | | 3.70E-03 |
| ENSG00000171236 | | LRG1 | | | | 0.25 | | | 7.85E-01 | | -1.60 | | | | 1.32E-06 | | 1.71 | | | | 1.08E-03 |
| ENSG00000100422 | | CERK | | | | -0.10 | | | 8.95E-01 | | -1.72 | | | | 1.33E-20 | | 1.10 | | | | 4.48E-04 |
| ENSG00000198934 | | MAGEE1 | | | | -0.19 | | | 8.72E-01 | | -1.76 | | | | 1.40E-06 | | 2.08 | | | | 1.54E-04 |
| ENSG00000137501 | | SYTL2 | | | | -0.48 | | | 1.42E-01 | | -1.94 | | | | 8.75E-19 | | 1.80 | | | | 1.19E-07 |
| ENSG00000110375 | | UPK2 | | | | 0.52 | | | 1.10E-01 | | -1.96 | | | | 2.07E-16 | | 1.47 | | | | 1.17E-04 |
| ENSG00000234176 | | HSPA8P1 | | | | 0.32 | | | 8.73E-01 | | -2.15 | | | | 4.29E-03 | | 2.92 | | | | 6.14E-03 |
| ENSG00000126803 | | HSPA2 | | | | -0.69 | | | 6.25E-04 | | -2.22 | | | | 5.22E-36 | | 2.23 | | | | 1.25E-17 |
| ENSG00000186226 | | LCE1E | | | | 0.55 | | | 5.45E-01 | | -2.23 | | | | 5.41E-05 | | 2.10 | | | | 1.61E-02 |
| ENSG00000137745 | | MMP13 | | | | -4.13 | | | 1.69E-54 | | -2.24 | | | | 2.11E-25 | | 2.67 | | | | 2.10E-10 |
| ENSG00000269399 | | CTD-3222D19.12 | | | | -0.96 | | | 1.99E-01 | | -2.39 | | | | 1.35E-04 | | 2.87 | | | | 2.42E-03 |
| ENSG00000109971 | | HSPA8 | | | | -0.10 | | | 9.28E-01 | | -2.59 | | | | 6.04E-28 | | 2.82 | | | | 1.99E-15 |
| ENSG00000204388 | | HSPA1B | | | | 0.03 | | | 9.82E-01 | | -2.84 | | | | 7.20E-36 | | 2.94 | | | | 7.70E-19 |
| ENSG00000102243 | | VGLL1 | | | | -1.08 | | | 6.99E-02 | | -3.05 | | | | 1.57E-08 | | 2.64 | | | | 2.60E-03 |
| ENSG00000145198 | | VWA5B2 | | | | -1.49 | | | 7.49E-03 | | -3.19 | | | | 2.86E-08 | | 3.07 | | | | 6.95E-04 |
| ENSG00000204389 | | HSPA1A | | | | 0.09 | | | 9.31E-01 | | -3.51 | | | | 5.06E-57 | | 3.55 | | | | 1.38E-29 |
| ENSG00000136383 | | ALPK3 | | | | -0.70 | | | 3.15E-01 | | -3.59 | | | | 4.78E-09 | | 3.49 | | | | 3.30E-05 |

| **Supplementary Table 4.** Gene set enrichment analysis (GSEA) results for IFN-γ-induced genes that were significantly modified by EGF (*P_interaction_* < 0.05) using the KEGG pathway database | | | | | | | | |
| --- | --- | --- | --- | --- | --- | --- | --- | --- |
| **Gene Set** | **Description** | **Size** | **Leading Edge Number** | | **ES** | **NES** | **P Value** | **FDR** |
| hsa04060 | Cytokine-cytokine receptor interaction | 10 | 5 | | 0.76694 | 2.1077 | <0.001 | 0.003 |
| hsa05169 | Epstein-Barr virus infection | 6 | 5 | | 0.79609 | 1.8943 | <0.001 | 0.012 |
| hsa05323 | Rheumatoid arthritis | 5 | 4 | | 0.82531 | 1.8293 | 0.003 | 0.025 |
| hsa04621 | NOD-like receptor signaling pathway | 5 | 3 | | 0.80372 | 1.7905 | 0.005 | 0.029 |
| hsa04151 | PI3K-Akt signaling pathway | 8 | 6 | | 0.63123 | 1.6447 | 0.036 | 0.057 |
| hsa04010 | MAPK signaling pathway | 10 | 4 | -0.39303 | | -1.3991 | 0.085 | 0.109 |

ES, enrichment score; FDR, false-discovery rate; KEGG, Kyoto Encyclopedia of Genes and Genomes; NES, normalized enrichment score.

**Supplementary Table 5.** Top enriched GO Biologic Processes among all EGF-induced DEGs (n=938) and the subset of EGF-induced DEGs with 2-fold higher expression in the presence of IFN-γ (n=45)^1^

| **GO ID** | **GO Name** | **C** | **O** | **P-value** | **FDR** |
| --- | --- | --- | --- | --- | --- |
| *Among EGF-induced DEGs (n=938)* | | | | | |
| GO:0009888 | tissue development | 1422 | 155 | 0 | 0 |
| GO:0016477 | cell migration | 1087 | 122 | 0 | 0 |
| GO:0050896 | response to stimulus | 5237 | 370 | 0 | 0 |
| GO:0060429 | epithelium development | 866 | 102 | 3.33E-16 | 7.93E-13 |
| GO:0009605 | response to external stimulus | 1718 | 161 | 4.44E-16 | 7.93E-13 |
| GO:0040011 | locomotion | 884 | 103 | 4.44E-16 | 7.93E-13 |
| GO:0048513 | animal organ development | 2012 | 180 | 4.44E-16 | 7.93E-13 |
| GO:0048870 | cell motility | 1182 | 125 | 4.44E-16 | 7.93E-13 |
| GO:0009653 | anatomical structure morphogenesis | 1865 | 168 | 3.66E-15 | 5.82E-12 |
| GO:0051716 | cellular response to stimulus | 4458 | 314 | 8.44E-15 | 1.21E-11 |
| *Among EGF-induced DEGs with >2-fold higher expression in presence IFN-γ (n=45)* | | | | | |
| GO:0044419 | interspecies interaction between organisms | 1073 | 16 | 4.83E-09 | 0 |
| GO:0002376 | immune system process | 1671 | 19 | 5.72E-09 | 0 |
| GO:0009605 | response to external stimulus | 1718 | 19 | 9.21E-09 | 0 |
| GO:0006955 | immune response | 1137 | 16 | 1.13E-08 | 0 |
| GO:0006952 | defense response | 1149 | 18 | 1.07E-10 | 1.5289E-06 |
| GO:0051707 | response to other organism | 964 | 16 | 9.96E-10 | 4.8904E-06 |
| GO:0043207 | response to external biotic stimulus | 966 | 16 | 1.03E-09 | 4.8904E-06 |
| GO:0009607 | response to biotic stimulus | 989 | 16 | 1.46E-09 | 5.1973E-06 |
| GO:0098542 | defense response to other organism | 764 | 13 | 5.85E-08 | 0.0001 |
| GO:0006950 | response to stress | 2538 | 21 | 1.39E-07 | 0.0002 |

C: Number of genes in the category; O: Number of overlapped genes in the subnetwork and the category

^1^Using Network Topology Analysis (NTA) analogous to that presented for IFN-γ induced DEGs in Figure 2. No GO Biologic Processes were enriched among the subset of EGF-induced DEGs with 2-fold lower expression in the presence of IFN-γ (n=26)


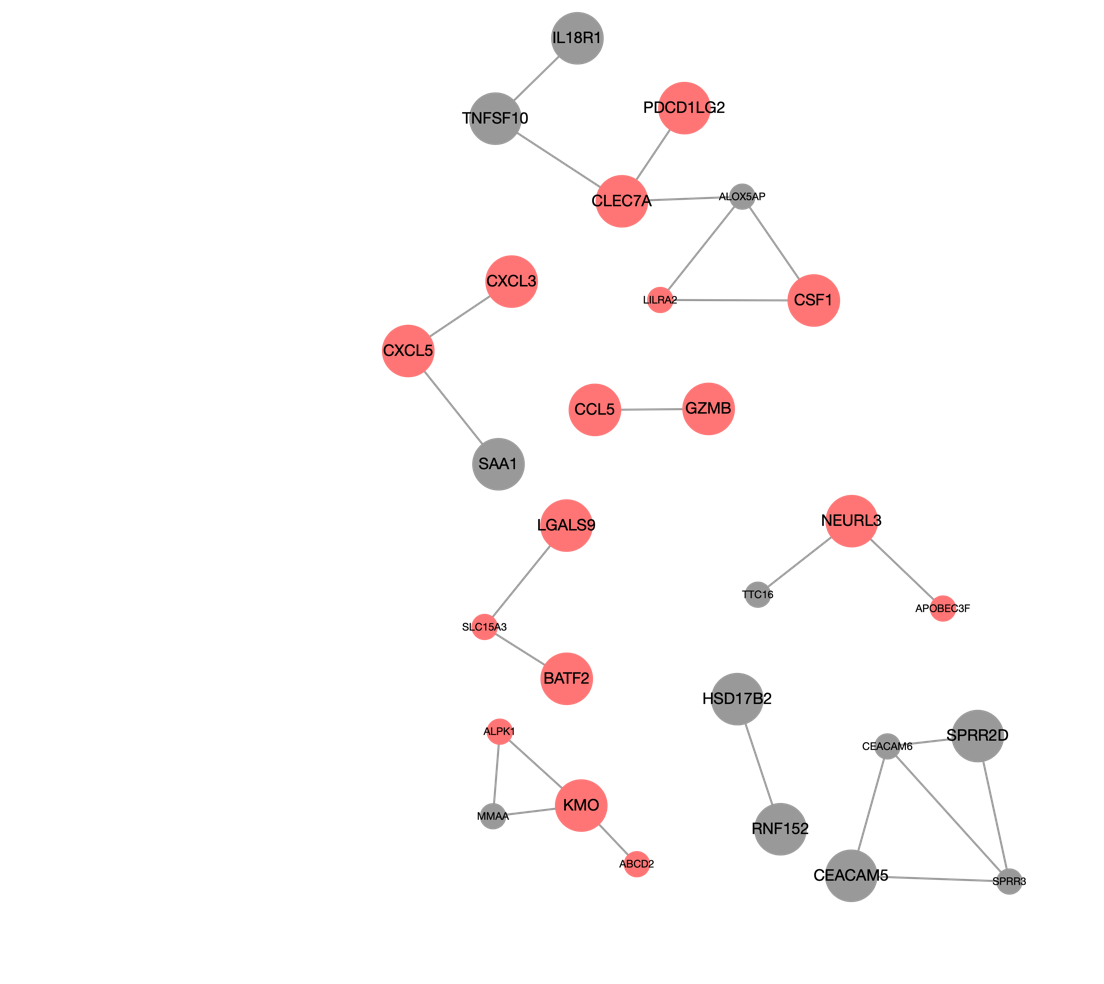


**Supplementary Figure 5.** Gene network from Network Topology Analysis (NTA) of EGF-induced genes whose expression was 2-fold higher in the presence of IFN-γ. Genes in the top enriched GO Biological Process category are shaded in red (GO:0044419, biologic processes involved in interspecies interaction between organisms).
